# Long-term outcomes of surgical repair versus replacement for tricuspid valve endocarditis − A *meta*-analysis of reconstructed time-to-event data

**DOI:** 10.1016/j.ijcha.2025.101782

**Published:** 2025-08-28

**Authors:** Eric Katsuyama, Christian Fukunaga, Felipe S. Passos, Nicole Lee, Ana Carolina Ventura de Santana de Jesus, Camila M. Ydy, Sofia Junqueira Franco Massuda, Hristo Kirov, Torsten Doenst, Tulio Caldonazo

**Affiliations:** a Department of Medicine, Centro Universitário Faculdade de Medicina do ABC, Santo André, São Paulo, Brazil; b Department of Thoracic Surgery, MaterDei Hospital, Salvador, Bahia, Brazil; c Department of Medicine, Federal University of São Paulo, São Paulo, Brazil; d Department of Medicine, Escola Bahiana de Medicina e Saúde Pública, Salvador, Bahia, Brazil; e Department of Medicine, Universidade de Ribeirão Preto, Ribeirão Preto, São Paulo, Brazil; f Department of Cardiothoracic Surgery, Jena University Hospital, Friedrich Schiller University of Jena, Jena, Germany; g Department of Cardiothoracic Surgery, Well Cornell Medicine, NY, United States of America

**Keywords:** Tricuspid valve, Repair And replacement, Endocarditis

## Abstract

**Introduction:**

Tricuspid valve infective endocarditis (TVIE) is surgically managed by tricuspid valve repair (TVr) or replacement (TVR). However, the differences in long-term endpoints and perioperative complications between the two strategies remain unclear. Therefore, this updated *meta*-analysis aimed to evaluate the efficacy and safety of TVr compared with TVR.

**Methods:**

MEDLINE, EMBASE, Cochrane Library, LILACS, and ClinicalTrials.gov were searched. The endpoints of interest were long-term all-cause mortality (primary), any reoperation, reinfection, postoperative stroke, and postoperative acute kidney injury (AKI). Data are reported as hazard ratios (HR) and odds ratios (OR) with their respective 95% confidence intervals (CI).

**Results:**

We included 19 retrospective cohorts comprising 9,734 patients, of which 59.7 % received TVr and 74.3 % were intravenous drug users. One study was risk-adjusted. The median age and follow-up were 35.9 years and 3.9 years, respectively. Compared with TVR, TVr was associated with lower long-term mortality (HR: 0.77; 95 %CI: 0.60 to 0.98; P = 0.04) and lower odds of any reoperation (OR: 0.73; 95 %CI: 0.60 to 0.89; P < 0.01), reinfection (OR: 0.40; 95 %CI: 0.19 to 0.86; P = 0.02), and postoperative AKI (OR: 0.79; 95 %CI: 0.68 to 0.92; P < 0.01). No differences were found in postoperative stroke (OR: 1.17; 95 %CI: 0.83 to 1.65; P = 0.41).

**Conclusion:**

In this *meta*-analysis, TVr improved overall survival and reduced postoperative complications in patients with TVIE. A possible treatment allocation bias needs to be considered as a potential concern of series with observational nature.

## Highlights

• This updated systematic review and *meta*-analysis of 19 studies with 9,734 tricuspid valve infective endocarditis (TVIE) patients, we compared the tricuspid valve repair (TVr) and tricuspid valve replacement (TVR).
• TVr has long-term benefits, including lower long-term mortality, any reoperation and reinfection rates compared to TVR.
• Additionally, TVr has a better safety profile, with reduced rates of postoperative acute kidney injury and deep wound infection. However, no differences were observed in postoperative stroke and early mortality.
• In isolated TVIE, long-term mortality did not differ significantly between TVr and TVR.

## Graphical abstract

**Figure:**
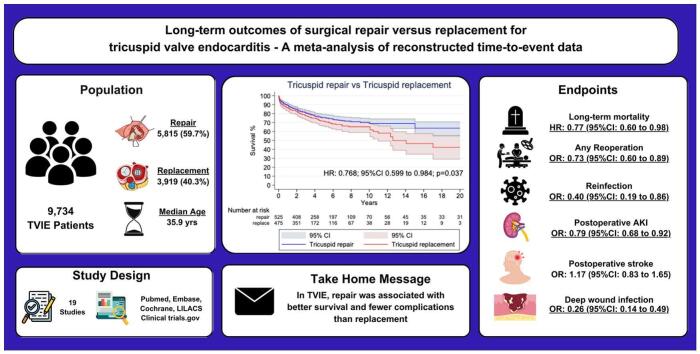

## 1 Introduction

Approximately 10 % [1] of patients with infective endocarditis (IE) develop right-sided IE, a condition predominantly associated with intravenous drug users (IVDU) [2,3]. Most cases are resolved by antibiotic therapy alone [4], and surgical management is reserved for cases of persistent bacteremia, severe right ventricular dysfunction caused by tricuspid regurgitation, concomitant left-sided involvement, and large vegetation [4]. However, in the past decade, an increasing trend in right-sided IE surgeries, especially tricuspid valve (TV) procedures, has been observed, which is likely caused by the increasing incidence of IVDUs [5].

IE surgery primarily focuses on removing vegetation and the remaining infected tissues while minimizing the use of prosthetic materials [4], and most guidelines recommend TV repair (TVr) as the preferred approach. TV replacement (TVR) is reserved in cases in which lesion extension makes repair impossible [6]. Nevertheless, TVr is not widely implemented in most healthcare services, leaving TVR as the only available option [7]. Furthermore, the efficacy and safety of these surgical approaches in patients undergoing isolated TVIE surgery remain unknown.

A previous *meta*-analysis [8] reported that TVr is associated with a lower risk of reoperation, pacemaker implantation, and reinfection. However, most of the analyses used a small sample size to analyze long-term survival, and other complications, such as postoperative stroke, deep sternal wound infection, and acute kidney injury (AKI), were not included as endpoints. In addition, recent studies [[9], [10], [11]] involving a larger number of patients in contemporary settings have been published. Therefore, this updated systematic review and *meta*-analysis aimed to compare the effectiveness and safety of TVr with those of TVR in patients with tricuspid IE.

## 2 Methods

### 2.1 Study design

The present systematic review and *meta*-analysis were designed according to the Cochrane Handbook and Preferred Reporting Items for Systematic Reviews and Meta-Analysis (PRISMA) [12,13]. The protocol was registered and approved by the International Prospective Register of Systematic Reviews, using the identification code CRD420251015463. The complete PRISMA checklist is available in Supplementary Method 1.

### 2.2 Search strategy and screening process

MEDLINE/Pubmed, EMBASE, CENTRAL/Cochrane Library, LILACS, and Clinical Trials.gov were systematically searched from inception to the final search date (February 3rd). Four independent authors (C.K., A.V., C.M. and S.J.) searched for the following keyword: “Tricuspid Valve,” “TV,” “repair,” “reconstruction,” “replacement,” “surgery,” and “endocarditis” in a double-blinded approach. A comprehensive list of all the search strategies used for each database is provided in Supplementary Methods 2. After removing duplicates, citations were screened by title and abstract, followed by full-text assessment following our eligibility criteria. Disagreements were resolved through a panel discussion with a third author (E. K.).

### 2.3 Eligibility criteria

Our eligibility criteria were as follows: (1) reported IE and TV involvement; (2) compared TVr with TVR; (3) reported any endpoint of interest; and (4) included patients undergoing either isolated TV surgery or concomitant procedures involving other valves. We restricted our exclusion criteria to: (1) case series and reports; (2) did not stratify the endpoints into TVr and TVR groups; (3) studies with TV surgery without IE subgroup; and (3) abstract from conferences. No restrictions on the publication date, language, or follow-up period were applied. The detailed eligibility criteria for each included study are available in Supplementary Methods 3.

### 2.4 Endpoints and subanalyses

We defined our primary endpoint as long-term all-cause mortality. We collected data on the following secondary endpoints: postoperative stroke, AKI, deep wound infection, reoperation, reinfection, permanent pacemaker implantation, and early all-cause mortality. Early all-cause mortality was defined as in-hospital, 30-day, or operative mortality [14,15]. The endpoint definitions for each eligible study are presented in Supplementary Method 4. We also performed a prespecified subanalysis of patients undergoing isolated TV surgery. Data extraction was performed independently by two authors, (C.K. and N.L.) and discrepancies were resolved by a third author (E.K).

### 2.5 Quality assessment

We performed a quality assessment using Cochrane’s risk of bias in non-randomized studies of interventions (ROBINS-I) [16] for all the included observational studies. This tool classifies the risk of bias into “low,” “moderate,” “serious” and ”critical” risk of bias based on the following domains: (1) risk of bias due to confounding; (2) risk of bias in classification of interventions; (3) Risk of bias in selection of participants into the study (or into the analysis); (4): risk of bias due to deviations from intended interventions; (5) risk of bias due to missing data; (6) risk of bias arising from the measurement of the outcome; and (7) risk of bias in selection of the reported result. In addition, we used the Grading of Recommendations, Assessment, Development, and Evaluation (GRADE) to evaluate the certainty of our results [17].

Two independent authors (C.K. and E.K.) and any disagreements were resolved through panel discussion between the authors. Additionally, the small study effect and publication bias were visually inspected using funnel plots [18], and if the endpoint presented more than ten studies, the Egger test [19].

### 2.6 Statistical analysis

We performed a pairwise *meta*-analysis pooling the estimated effect sizes using odds ratios (OR) for binary data and hazard ratios (HR) for time-to-event data with their corresponding 95 % confidence intervals (CI). The study weights were calculated using the Mantel-Haenszel (MH) approach. A two-sided p-value below 0.05 was set as statistically significant for the estimated effect sizes. Between-study heterogeneity was deemed relevant; therefore, we adopted a random-effects model using the restricted maximum likelihood method (REML) to estimate tau^2^. Heterogeneity was assessed using the Cochrane Q test and I^2^ statistics, with the thresholds of p-value > 0.1 and I^2^ > 25 %, respectively, considered indicative of substantial heterogeneity. If a study reported an adjusted effect size, we applied a generic inverse-of-variance approach to pool the overall effect size. Zero events were addressed by using the MH zero-cell correction approach [20]. Sensitivity analyses were conducted using the leave-one-out method [21] to explore the impact of individual studies on heterogeneity. Additionally, post hoc subgroup analyses were performed to evaluate: (1) the impact of high and moderate risk of bias studies for long-term all-cause mortality; and (2) bioprosthetic versus mechanical TVR for early all-cause mortality.

### 2.7 One-Stage survival analysis

For the long-term all-cause mortality endpoint, we reconstructed individual patient data (IPD) from the reported Kaplan-Meier (KM) curves of the included studies, following the methods described by Wei [22] and Guyot [23]. First, the raster and vector images were processed and digitized to extract the survival/mortality values at specific time points. When available, number-at-risk tables or total event counts were used to calibrate the time-to-event data estimations. To ensure the robustness of our findings, we compared our estimated survival with data reported in the original publications. Finally, we generated our overall survival curves, and a one-stage *meta*-analysis was performed using the Cox proportional regression model, reported in HR and 95 %CI. The proportional hazards assumption was verified by plotting scaled Schoenfeld residuals. As sensitivity analyses, a landmark analysis and a subgroup analysis involving just isolated TV surgery were performed to check the robustness of the findings.

All statistical analyses were performed under R Studio 4.4.3(R Foundation for Statistical Computing, Vienna, Austria) using packages “meta” [24] and STATA version 17 (Stata Corp, College Station, TX, USA).

## 3 Results

After screening 4,815 potential citations, 123 were deemed eligible for the full-text review. Of these, 104 were excluded based on our eligibility criteria, resulting in the inclusion of 19 studies [[9], [10], [11],[25], [26], [27], [28], [29], [30], [31], [32], [33], [34], [35], [36], [37], [38], [39], [40]]. The PRISMA flowcharts and reasons for exclusion during the full-text review are shown in Supplementary Fig. 1 and Supplementary Table 1, respectively. All included studies were retrospective cohorts encompassing 9,734 patients with TVIE, of whom 5,815 (59.7 %) underwent the TVr procedure. One study [9] was risk-adjusted. The median age and follow-up were 35.9 years and 3.9 years, respectively. At baseline, our cohort included 53.8 % female and 74.3 % IVDU, based on studies reporting these covariates. The characteristics of the included studies are shown in Table 1 and the demographics of the target cohort are available in Supplementary Table 2*.* Other information, such as indications for surgery, operation description, and causative microorganisms, are available in Supplementary Tables 3,4 and 5, respectively.

**Table 1:** Study characteristics of the included studies.

**Study, Year**	**Country**	**Sample Size**			**Study Design**	**Endocarditis Criteria**	**Inclusion Criteria**	**Exclusion Criteria**
**Baraki, 2013**[25]	Japan	33	TVr	15 (45.5)	Retrospective cohort Single center	Duke criteria	Isolated TV repair or replacement due to endocarditis	NR
			TVR	18 (54.5)				
**Brescia, 2022**[26]	United States Of America	71	TVr	37 (52.1)	Retrospective cohort Single center	Society of Thoracic Surgeons Adult Cardiac Surgery database criteria	Patients with endocarditis undergoing TVr and TVR. Concomitant CABG, anti-arrhythmia procedure, and with previous cardiac surgery unrelated to mitral or TV were also included	Aortic, pulmonic, or both left right sided endocarditis
			TVR	34 (47.9)				
**Dawood, 2015**[27]	United States	56	TVr	32 (57.1)	Retrospective cohort Single center	Duke Criteria	Patients who underwent TVIE repair or replacement operations	Rheumatic valve and functional tricuspid disease
			TVR	24 (42.9)				
**Di Mauro, 2022**[28]	Italy	149	TVr	77 (51.7)	Retrospective cohort Multicentered	NR	Isolated acute TVIE with native tricuspid regurgitation or stenosis and regurgitation undergoing repair or replacement	Tricuspid prothesis endocarditis
			TVR	72 (48.3)				
**Dzilic,** **2022**[29]	Germany	32	TVr	16 (50)	Retrospective cohort Single center	NR	Isolated TVIE undergoing repair or replacement	Concomitant procedures
			TVR	16 (50)				
**Gaca, 2013**[30]	United States Of America	844	TVr	354 (41.9)	Retrospective cohort Single center	Society of Thoracic Surgeons Adult Cardiac Surgery database criteria	Patients between 18 to 90 years old with TVIE undergoing TVr or TVR Concomitant procedures for ventricular septal defect and arrhythmia were also included	Missing data on sex, surgery status, cardiogenic shock, endocarditis’s type, and prior valve surgery
			TVR	490 (58.1)				
**Gottardi,** **2007**[31]	Austria	22	TVr	18 (81.8)	Retrospective cohort Single center	TTE and Duke Criteria	Patients with endocarditis undergoing TVr and TVR	NR
			TVR	4 (18,2)				
**Jawad,** **2020**[32]	Egypt	223	TVr	95 (42.6)	Retrospective cohort Single center	NR	IVDU undergoing isolated TVIE repair or replacement	Under 18 years old and over 60 years old. Patients referred from correctional institutions or with preoperative hemoglobin < 6, white blood cells > 16,000 cell/ml, and platelets < 90,000/ml were also excluded
			TVR	128 (57.4)				
**Lee,** **2020**[9]	Taiwan	704	TVr	412 (58.5)	Retrospective cohort Multicentered	NR	Patients with a TVIE before or during index hospitalization	Younger than 20 years old or undergoing both TVr and TVR
			TVR	292 (41.5)				
**Musci,** **2007**[33]	Germany	73	TVr	42 (57.5)	Retrospective cohort Single center	NR	Patients with endocarditis undergoing TVr and TVR	NR
			TVR	31 (42.5)				
**Pfannmueller, 2015**[34]	Germany	56	TVr	34 (60.7)	Retrospective cohort Single center	NR	Patients with endocarditis undergoing TVr and TVR	NR
			TVR	22 (39.3)				
**Protos,** **2018**[35]	United States of America	38	TVr	12 (31.6)	Retrospective cohort Single center	NR	Patients with endocarditis undergoing TVr and TVR	Patients under 18 years old
			TVR	26 (68.4)				
**Renzulli,** **1999**[36]	Italy	21	TVr	11 (52.4)	Retrospective cohort Single center	Duke criteria and echocardiography	Patients with endocarditis undergoing TVr and TVR	NR
			TVR	10 (47.6)				
**Siddiqui,** **2022**[37]	United States of America	894	TVr	353 (39.5)	Retrospective cohort Single center	NR	Patients with endocarditis undergoing TVr and TVR. Also included patients with pulmonary embolism on admission	NR
			TVR	541 (60.5)				
**Shetty,** **2015**[38]	Canada	7	TVr	5 (71.4)	Retrospective cohort Single center	TTE or TEE confirmation	Native TVIE undergoing TVr or TVR	Under 18 years old, non-infective cause of endocarditis
			TVR	2 (28.6)				
**Slaughter, 2019**[39]	Multinational	1,494	TVr	532 (35.6)	Retrospective cohort Multicentered	NR	Patients with endocarditis undergoing TVr and TVR	Concomitant surgeries, reoperations or severe aortic or mitral insufficiency were excluded. Patients without IVDA and history of aortic mitral valve IE or TV endocarditis were also excluded
			TVR	962 (64.4)				
**Thourani,** **2021**[40]	Multinational	4,831	TVr	3,654 (75.6)	Retrospective cohort Multicentered	NR	Patients aged 18 years old who underwent isolated TVr or TVR.	Valvectomy, previous prosthetic valve procedures or pannus thrombus removal procedures, transcatheter concomitant surgeries with mitral repairs or replacements
			TVR	1,177 (24.4)				
**Witten,** **2018**[10]	United States of America	130	TVr	93 (71.5)	Retrospective cohort Single center	Modified Duke criteria	Patients with endocarditis undergoing TVr and TVR	NR
			TVR	37 (28.5)				
**Xie,** **2023**[11]	China	56	TVr	23 (41.1)	Retrospective cohort Single center	TTE and Duke Criteria or postoperative pathology	Patients with endocarditis undergoing TVr and TVR	NR
			TVR	33 (58.9)				

All binary data are reported as counts and frequencies n (%). Society of Thoracic Surgeons Adult Cardiac Surgery database criteria: IE is defined as an operated valve diagnosed through reoperation findings, autopsy evidence, or fulfillment of the Duke Criteria[51]. Abbreviations: CABG: Coronary Artery Bypass Graft Surgery; IE: Infective endocarditis; IVDU: Intravenous drug use; NR: not reported; TEE, transesophageal echocardiography; TTE: transthoracic echocardiogram; TVr: Tricuspid Valve repair; TVR: Tricuspid Valve Replacement; TVIE: Tricuspid Valve Infective Endocarditis.

### 3.1 One-Stage survival analysis

**Fig. 1:**
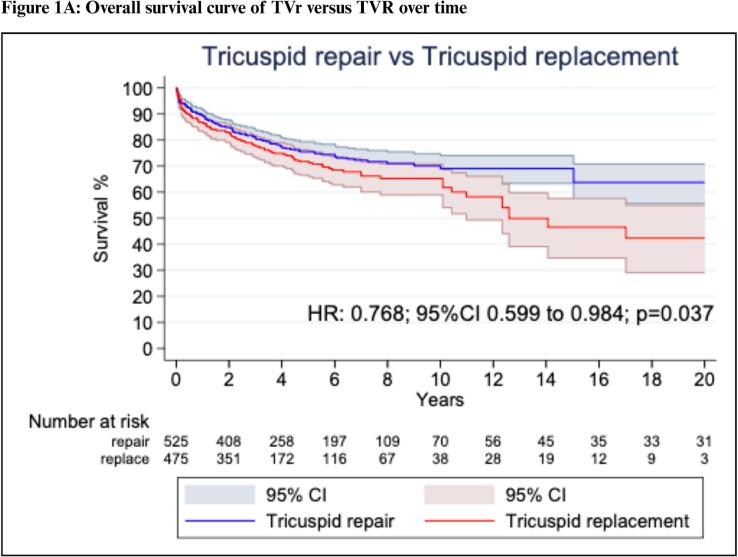

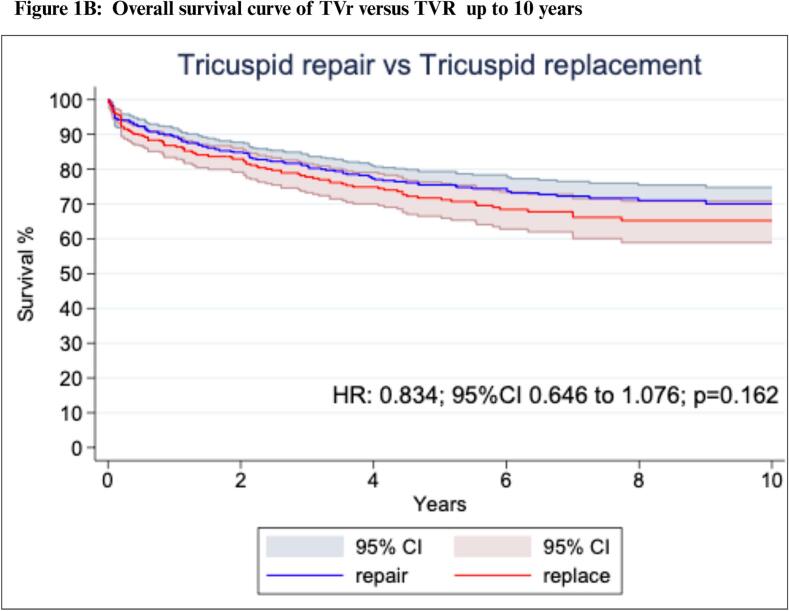

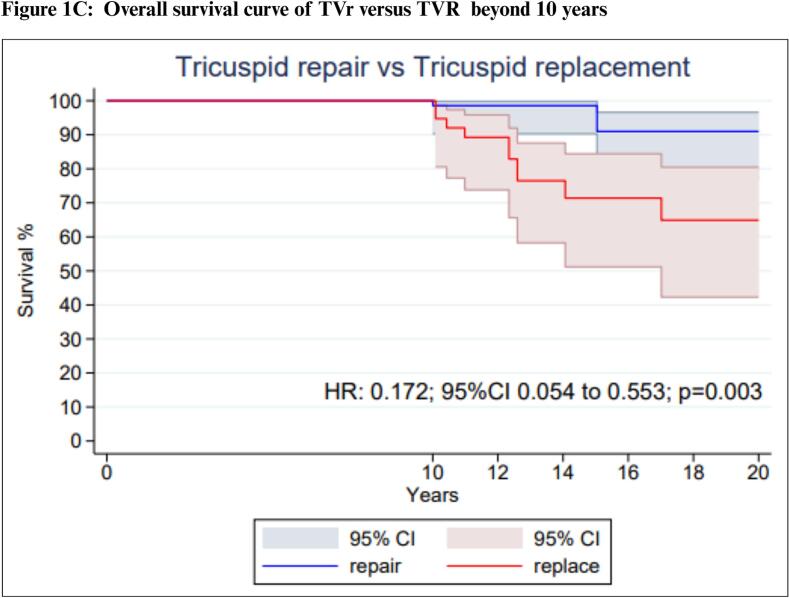
**Reconstructed One-Stage Survival Analysis.** Survival curves TVR vs. TVr over time: overall (A) up to 10 years. (B) and beyond 10 years (C). Abbreviations: CI: Confidence Interval; HR: Hazard Ratio; TVr: Tricuspid Valve repair; TVR: Tricuspid Valve Replacement.

Our one-stage survival analysis for the long-term all-cause mortality endpoint included 8 studies with 1,000 patients. A comparison between the extracted and reconstructed KM curves is presented in Supplementary Results 1*.* Patients with TVIE who underwent TVr had a significantly lower long-term mortality than those who underwent TVR (HR: 0.77; 95 %CI: 0.60 to 0.98; P = 0.04; Fig. 1*A*).

Violation of the proportional hazard assumption was observed between scaled Schoenfeld residuals and follow-up time which indicated that the HR was not constant over time (P = 0.03; Supplementary Fig. 2). Since we observed that the proportional hazards assumption was violated, we proceeded with landmark analysis, designating 10 years as the landmark time point according to the oscillation of HR over time (Fig. 1*B*).

In the 10-years landmark analysis, no significant difference was observed between the TVr and TVR groups (HR: 0.83; 95 %CI: 0.65 to 1.08; P = 0.16; Fig. 1*B*). However, after 10 years, TVr was again associated with significantly reduced mortality rates compared to TVR (HR: 0.17; 95 %CI: 0.05 to 0.55; P = 0.03, Fig. 1*C*).

### 3.2 Subanalysis for isolated tricuspid valve cohort

In this sub-analysis of long-term all-cause mortality, we included three trials [10,27,32] with 434 patients who underwent isolated TVIE surgery, in which TVr and TVR were not significantly different in long-term mortality rates than TVR (HR: 0.62; 95 % CI: 0.37 to 1.06; P = 0.08, Supplementary Fig. 3).

### 3.3 Two-stage *meta*-analysis

**Fig. 2:**
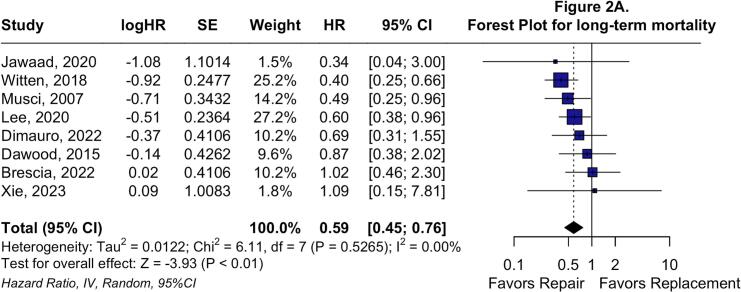

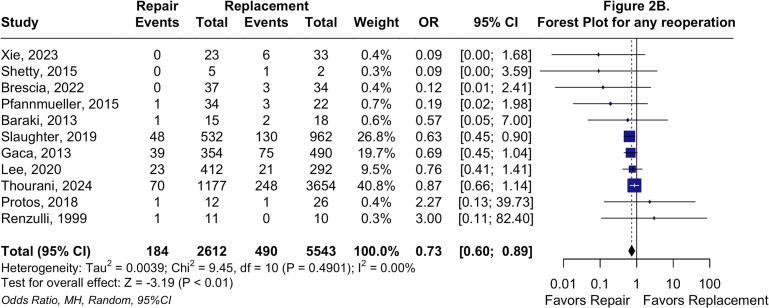

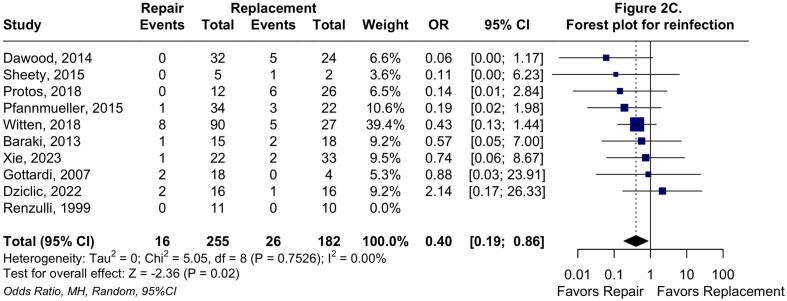
**Forest plots for long-term endpoints.** Forest plot summarizing results for long-term all-cause mortality (A), forest plot summarizing results for any reoperation (B), and forest plot summarizing results for reinfection. Abbreviations: CI: Confidence Interval; HR: Hazard Ratio; IV: Inverse variance; MH: Mantel-Haenszel; OR: Odds Ratio; TVr: Tricuspid Valve repair; TVR: Tricuspid Valve Replacement.

In the two-stage *meta*-analysis using the random effects model, TVr was associated with a significantly decreased long-term all-cause mortality rate compared with TVR (HR: 0.59; 95 %CI: 0.45 to 0.76; P < 0.01; I^2^ = 0 %; Fig. 2*A*), consistent with the one-stage analysis. TVr was also associated with significantly lower odds of reoperation (OR: 0.73; 95 %CI: 0.60 to 0.89; P < 0.01; I^2^ = 0 %; Fig. 2*B*) and reinfection (OR: 0.40; 95 %CI: 0.19 to 0.86; P = 0.02; I^2^ = 0 %; Fig. 2*C*).

**Fig. 3:**
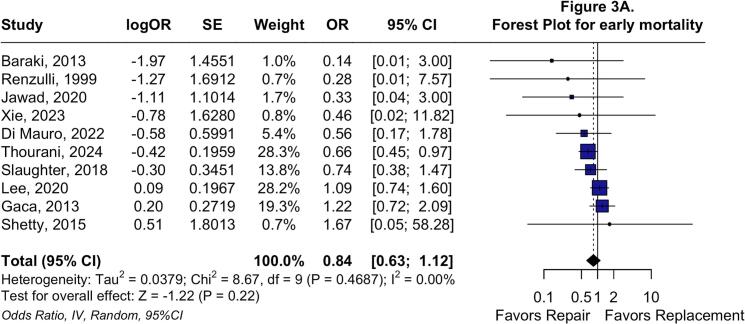

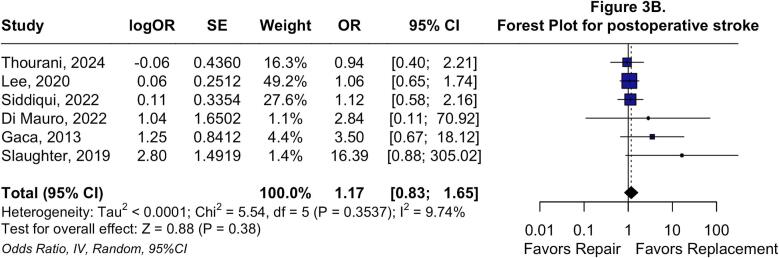

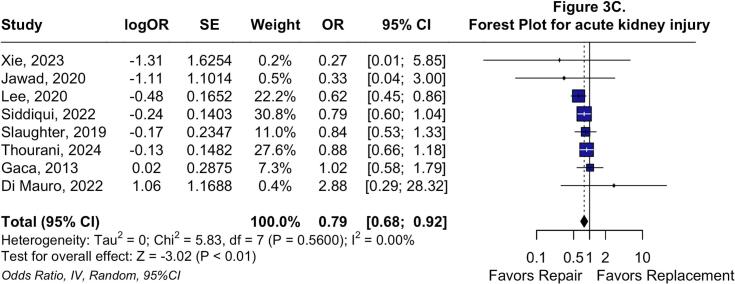
**Forest plots for Early endpoints.** Forest plot summarizing results for early cause mortality (A), forest plot summarizing results for postoperative stroke (B), and forest plot summarizing results for acute kidney injury. *Abbreviations:* CI: Confidence Interval; MH: Mantel-Haenszel; OR: Odds Ratio; TVr: Tricuspid Valve repair; TVR: Tricuspid Valve Replacement.

Regarding early endpoints, no differences were observed in early mortality (OR: 0.84; 95 %CI: 0.63 to 1.12; P = 0.22; I^2^ = 0 %; Fig. 3*A*) or postoperative stroke (OR: 1.17; 95 %CI: 0.83 to 1.65; P = 0.41; I^2^ = 9.7 %; Fig. 3*B*). Nevertheless, TVr was associated with lower odds of postoperative AKI (OR: 0.79; 95 %CI: 0.68 to 0.92; P < 0.01; I^2^ = 0 %; Fig. 3*C*), deep sternal wound infection (OR: 0.26; 95 %CI: 0.14 to 0.49; P < 0.01; I^2^ = 0 %; Supplementary Fig. 4*A*), and permanent pacemaker implantation (OR: 0.15; 95 %CI: 0.06 to 0.38; P < 0.01; I^2^ = 63.6 %; Supplementary Fig. 4*B*).

### 3.4 Sensitivity and post hoc subgroup analyses

Our leave-one-out analysis did not identify any study that affected the effect size or heterogeneity across most of our included endpoints (Supplementary Fig. 5*A–5H*). Nevertheless, after omitting one trial [39], heterogeneity in the postoperative stroke endpoint decreased substantially (OR: 1.13; 95 %CI: 0.80 to 1.59; I^2^ = 0 %; Supplementary Fig. 5*E*).

A post hoc subgroup analysis for high and moderate risk of bias studies for the long-term all-cause mortality endpoint did not detect a significant subgroup interaction (P for interaction = 0.86, Supplementary Fig. 6*A*). Additionally, the type of valve (bioprosthetic or mechanical) in TVR was not associated with a statistically significant interaction in our post hoc subgroup analysis of early all-cause mortality (P for interaction = 0.43; Supplementary Fig. 6*B*).

### 3.5 Quality assessment

Our assessment using ROBINS-I evaluated the included studies with a serious [10,[25], [26], [27], [28], [29], [30], [31], [32], [33], [34], [35], [36],^38^,^39^] or moderate [9,11,37,40] risk of bias (Supplementary Fig. 7). Most of the studies had a high risk of bias in the confounding and selection of participants domains.

In most of the outcomes, no funnel plot presented visual asymmetry with studies equally distributed towards the pooled effect size, suggesting no signs of a small study effect or publication bias (Supplementary Figs. 8*A–H*). In addition, the Egger test analysis, which was conducted with more than ten studies, was consistent with this analysis; no endpoint presented quantitative evidence of publication bias. The early mortality endpoint (Supplementary Fig. 8D) presented with visual asymmetry; however, Egger’s test did not detect any publication bias (P = 0.19).

Our GRADE assessment evaluated all presented evidence with a very low level of certainty, primarily because of the high risk of bias (Supplementary Table 6).

## 4 Discussion

In the present updated systematic review and *meta*-analysis of 19 studies with 9,734 IE patients, we compared the TVr and TVR strategies. Our main findings were as follows: (1) the TVr group had higher long-term survival; (2) it was also associated with reduced odds of any reoperation, reinfection, AKI, postoperative deep wound infection, and permanent pacemaker implantation; (3) there was no difference in early mortality and postoperative stroke between the two surgical strategies; and (4) in the isolated TVIE subanalysis, overall survival was not different between the TVr and TVR groups.

Advancements in the TVr design and techniques have progressively enhanced the prognosis of patients with TVIE. The addition of ring annuloplasty, leaflet repair, and commissural procedures [41,42] has made TVr the first-line recommendation of most guidelines. However, this surgical technique relies on the expertise of the surgical team and, in some cases, the infected tissue is so large that TVR is the only available option [7]. Furthermore, periprocedural complications and differences in late survival between the two surgical approaches remain unclear. While previous studies in non-IE populations have also favored repair [43,44], infection-related challenges such as extensive tissue damage, increased risk of reinfection, and difficulty in achieving complete eradication make the comparison particularly relevant in the context of IE [6]. Given these limitations and the variability in clinical practice, the present study provides a more comprehensive comparison between TVr and TVR in patients with IE in a contemporary context.

The results of this study extend the findings of a previous *meta*-analysis [8] by using a larger sample size and statistical power to demonstrate an association between TVr and a reduction of 13 % in late mortality. Interestingly, these benefits are aligned with the survival rates observed in mitral valve repair [45], suggesting that surgical repair is an efficient approach for both valves. This can be attributed to structural valve deterioration and embolic complications associated with replacement surgery [46].

Nevertheless, our landmark analysis indicated that this disparity emerged after 10 years, with no differences in mortality rates between the two surgeries in the postoperative period or during the first decade. Notably, the reduced long-term survival observed in the TVR cohort may partly reflect treatment allocation bias. [47] In the context of infective endocarditis, patients with fewer comorbidities and less severe clinical profiles are more likely to be selected for valve repair [6]. Consequently, this group may demonstrate inherently better long-term outcomes, independent of the surgical technique. Additionally, the late survival decline observed in the TVr cohort may result from its progressive structural deterioration beyond 10 years, including worsening valve regurgitation, which may ultimately increase long-term mortality [48].

After TVR, patients with IE are more susceptible to bacterial colonization, owing to the presence of a newly implanted prosthetic valve [7,49]. Conversely, TVr preserves a greater proportion of native tissue, providing better protection against reinfection and, consequently, reoperation [50]. These biological advantages are reflected in the findings of the present study, which reported lower rates of reoperation and reinfection in the TVr group, thus confirming the results of a previous *meta*-analysis. Additionally, with the increase in IVDU users in the past decade [5], choosing an optimal surgical approach to avoid any chance of reinfection makes TVr a much more interesting choice for treating TVIE than TVR. Therefore, beyond the long-term survival benefits, patients with TVIE undergoing TVr also experience a 27 % reduction in the chance of any reoperation and a 60 % reduction in reinfection, supporting the long-term efficacy of this surgical approach.

IE patients undergoing valve surgery have the potential for embolic events such as stroke due to vegetation dislocation during manipulation [9]. In the present study, we did not observe any differences between TVr and TVR in the patients with postoperative stroke. Of note, one study was responsible [39] for the small heterogeneity in this endpoint, probably due to differences in stroke definition; however, omitting this cohort did not affect the overall results. In contrast, AKI, deep-wound sternal infection, and permanent pacemaker implantation had lower rates in the TVr group, indicating a much better safety profile for this surgical management compared to TVR.

Compared to the previous *meta*-analysis, the present *meta*-analysis of 19 studies and more than 9,000 patients not only confirmed their results but also provided adequate statistical power to demonstrate a significant association between TVr and reduced long-term mortality, as well as perioperative complications, such as AKI and deep wound infection. Moreover, the minimal between-study heterogeneity in most outcomes underscores the generalizability of our findings to other populations and study designs. In contrast, in our subanalysis restricted to isolated TVIE, no difference in survival was observed between TVr and TVR. This highlights the need for larger, dedicated studies on isolated tricuspid valve surgery to confirm these findings.

### 4.1 Study limitations

This *meta*-analysis has several limitations. First, the overall certainty of the evidence is low, primarily because of the high risk of bias related to confounders, which limits confidence in our results. The lack of adjustment for confounders in most studies, except that by Lee et al. [9], is a serious limitation that should be addressed in future investigations. Second, the TVR group might have been influenced by treatment allocation bias because patients with a worse prognosis or those who had already undergone TVr may have been more likely to undergo valve replacement. Anatomical aspects of the lesion, such as the presence of an abscess or lesion threading, are associated with higher morbidity and are factors not accounted for in classical cardiac surgery risk scores (e.g., STS Score/EuroSCORE). Third, as inherent in our study design, the included studies likely varied in patient characteristics, surgeon expertise, publication year, and endpoint definition. Although between-study heterogeneity was minimal, these differences were still concealed. Finally, a median of 74.3 %, of patients were identified as IVDU based only on studies that reported this information. Notably, the largest trial [40] included in this review did not provide data on IVDU status, which may have affected the estimated proportion. Given the importance of increasing IVDUs in the current context of TVIE, routinely reporting this variable is important to improve the applicability of future studies to different patient groups, particularly as the clinical characteristics and outcomes may differ significantly between IVDU and non-IVDU populations.

## 5 Conclusion

The present work reported that surgical management of TVIE with TVr was associated with improved overall survival and fewer perioperative complications than TVR. A possible treatment allocation bias needs to be considered as a potential concern in a series with an observational nature.

## Fundings

TC was funded by the Deutsche Forschungsgemeinschaft (DFG, German Research Foundation) Advanced Clinician Scientist Program OrganAge funding number 413668513, by the Deutsche Herzstiftung (DHS, German Heart Foundation) funding number S/03/23, and by the Interdisciplinary Center of Clinical Research of the Medical Faculty Jena. The authors declare no financial support or funding for the research, authorship, or publication of this study.

## Data availability

The data analyzed in this meta-analysis were extracted from previously published studies. All data sources have been referenced in the manuscript.

## CRediT authorship contribution statement

**Eric Katsuyama:** Writing – review & editing, Writing – original draft, Visualization, Project administration, Investigation, Formal analysis, Data curation, Conceptualization. **Christian Fukunaga:** Writing – review & editing, Writing – original draft, Project administration, Investigation, Data curation. **Felipe S. Passos:** Writing – review & editing, Writing – original draft, Project administration, Investigation, Formal analysis. **Nicole Lee:** Writing – review & editing, Writing – original draft, Investigation, Data curation. **Ana Carolina Ventura de Santana de Jesus:** Writing – review & editing, Writing – original draft, Investigation, Data curation. **Camila M. Ydy:** Writing – review & editing, Writing – original draft, Investigation, Data curation. **Sofia Junqueira Franco Massuda:** Writing – review & editing, Writing – original draft, Investigation, Data curation. **Hristo Kirov:** Writing – review & editing, Writing – original draft, Supervision. **Torsten Doenst:** Writing – review & editing, Writing – original draft, Supervision. **Tulio Caldonazo:** Writing – review & editing, Writing – original draft, Supervision, Methodology.

## Declaration of competing interest

The authors declare that they have no known competing financial interests or personal relationships that could have appeared to influence the work reported in this paper.

## References

[1] Akinosoglou K., Apostolakis E., Marangos M., Pasvol G. (2013 Sep). Native valve right sided infective endocarditis. Eur. J. Intern. Med..

[2] Delgado V., Ajmone Marsan N., de Waha S., Bonaros N., Brida M., Burri H. (2023 Oct 14). 2023 ESC guidelines for the management of endocarditis. Eur. Heart J..

[3] Akinosoglou K., Apostolakis E., Koutsogiannis N., Leivaditis V., Gogos C.A. (2012 Sep). Right-sided infective endocarditis: surgical management. Eur. J. Cardiothorac. Surg..

[4] Vervoort D., An K.R., Elbatarny M., Tam D.Y., Quastel A., Verma S. (2022 Sep). Dealing with the Epidemic of Endocarditis in people who inject drugs. Can. J. Cardiol..

[5] Geirsson A., Schranz A., Jawitz O., Mori M., Feng L., Zwischenberger B.A. (2020 Oct). The Evolving Burden of Drug Use Associated Infective Endocarditis in the United States. Ann. Thorac. Surg..

[6] Iaccarino A., Barbone A., Basciu A., Cuko E., Droandi G., Galbiati D. (2023 Sep 11). Surgical challenges in Infective Endocarditis: State of the Art. J. Clin. Med..

[7] Galeone A., Gardellini J., Perrone F., Francica A., Mazzeo G., Lucchetti M.R. (2024 May). Tricuspid valve repair and replacement for infective endocarditis. Indian J Thorac Cardiovasc Surg..

[8] Yanagawa B., Elbatarny M., Verma S., Hill S., Mazine A., Puskas J.D. (2018 Sep). Surgical Management of Tricuspid Valve Infective Endocarditis: a Systematic Review and Meta-Analysis. Ann. Thorac. Surg..

[9] Lee H.A., Chou A.H., Wu V.C.C., Chan Y.S., Cheng Y.T., Chang C.H. (2021 Apr 29). Nationwide cohort study of tricuspid valve repair versus replacement for infective endocarditis. Eur. J. Cardiothorac. Surg..

[10] Witten J.C., Hussain S.T., Shrestha N.K., Gordon S.M., Houghtaling P.L., Bakaeen F.G. (2019 Apr). Surgical treatment of right-sided infective endocarditis. J. Thorac. Cardiovasc. Surg..

[11] Xie L., Chen X., He J., Lin S., Chen X., Wu Q. (2023 Apr 28). Comparison of valvuloplasty and replacement for surgical treatment of tricuspid infective endocarditis. BMC Cardiovasc. Disord..

[12] Page M.J., McKenzie J.E., Bossuyt P.M., Boutron I., Hoffmann T.C., Mulrow C.D. (2021 Mar). The PRISMA 2020 statement: an updated guideline for reporting systematic reviews. BMJ.

[13] Higgins, Julian P. T., Thomas, James, Chandler, Jackie, Cumpston, Miranda, Li, Tianjing, Page, Matthew J., et al. Cochrane Handbook for Systematic Reviews of Interventions version 6.3 (updated February 2022) [Internet]. Cochrane; 2022. Available from: https://www.training.cochrane.org/handbook.

[14] Edmunds L.H., Clark R.E., Cohn L.H., Grunkemeier G.L., Miller D.C., Weisel R.D. (1996). Guidelines for reporting morbidity and mortality after cardiac valvular operations. Eur. J. Cardiothorac. Surg..

[15] Bowdish M.E., D’Agostino R.S., Thourani V.H., Schwann T.A., Krohn C., Desai N. (2021 Jun). STS Adult Cardiac Surgery Database: 2021 Update on Outcomes, Quality, and Research. Ann. Thorac. Surg..

[16] Sterne J.A., Hernán M.A., Reeves B.C., Savović J., Berkman N.D., Viswanathan M. (2016 Oct). ROBINS-I: a tool for assessing risk of bias in non-randomised studies of interventions. BMJ.

[17] Guyatt G.H., Oxman A.D., Vist G.E., Kunz R., Falck-Ytter Y., Alonso-Coello P. (2008 Apr 26). GRADE: an emerging consensus on rating quality of evidence and strength of recommendations. BMJ.

[18] Sterne J.A.C., Sutton A.J., Ioannidis J.P.A., Terrin N., Jones D.R., Lau J. (2011 Jul). Recommendations for examining and interpreting funnel plot asymmetry in meta-analyses of randomised controlled trials. BMJ.

[19] Egger M., Davey Smith G., Schneider M., Minder C. (1997 Sep 13). Bias in meta-analysis detected by a simple, graphical test. BMJ.

[20] Xu C., Furuya-Kanamori L., Zorzela L., Lin L., Vohra S. (2021 Jul). A proposed framework to guide evidence synthesis practice for meta-analysis with zero-events studies. J. Clin. Epidemiol..

[21] Gewehr D., Carvalho P.E.P. (2023). How to perform meta-analysis with R - Exploring Heterogeneity: Leave-one-out Analysis [internet]. Unpublished.

[22] Wei Y., Royston P. (2017 Oct). Reconstructing time-to-event data from published Kaplan-Meier curves. Stata J..

[23] Guyot P., Ades A.E., Ouwens M.J.N.M., Welton N.J. (2012 Feb). Enhanced secondary analysis of survival data: reconstructing the data from published Kaplan-Meier survival curves. BMC Med. Res. Methodol..

[24] Balduzzi S., Rücker G., Schwarzer G. (2019 Nov). How to perform a meta-analysis with R: a practical tutorial. Evid. Based Ment. Health.

[25] Baraki H., Saito S., Al Ahmad A., Fleischer B., Schmitto J., Haverich A. (2013). Surgical treatment for isolated tricuspid valve endocarditis- long-term follow-up at a single institution. Circ J.

[26] Brescia A.A., Watt T.M.F., Rosenbloom L.M., Williams A.M., Bolling S.F., Romano M.A. (2022). Patient and Surgeon Predictors of Mitral and Tricuspid Valve Repair for Infective Endocarditis. Semin. Thorac. Cardiovasc. Surg..

[27] Dawood M.Y., Cheema F.H., Ghoreishi M., Foster N.W., Villanueva R.M., Salenger R. (2015 Feb). Contemporary outcomes of operations for tricuspid valve infective endocarditis. Ann. Thorac. Surg..

[28] Di Mauro M., Bonalumi G., Giambuzzi I., Dato G.M.A., Centofanti P., Corte A.D. (2022 Jun 1). Similar outcome of tricuspid valve repair and replacement for isolated tricuspid infective endocarditis. J. Cardiovasc. Med. (Hagerstown).

[29] Dzilic E., Nöbauer C., Burri M., Voss S., Krane M., Lange R. (2022 Oct). Surgical treatment of isolated tricuspid valve endocarditis: Midterm data. J. Card. Surg..

[30] Gaca J.G., Sheng S., Daneshmand M., Rankin J.S., Williams M.L., O’Brien S.M. (2013 Oct). Current outcomes for tricuspid valve infective endocarditis surgery in North America. Ann. Thorac. Surg..

[31] Gottardi R., Bialy J., Devyatko E., Tschernich H., Czerny M., Wolner E. (2007 Dec). Midterm follow-up of tricuspid valve reconstruction due to active infective endocarditis. Ann. Thorac. Surg..

[32] Abd Al Jawad M., Ammar A., Nahas Y., Ahmed A., Kilany I., El Kerdany A. (2020 Apr). Evaluation of Tricuspid Valve Repair without Annuloplasty Ring in Intravenous Drug Abusers. Ann. Thorac. Surg..

[33] Musci M., Siniawski H., Pasic M., Grauhan O., Weng Y., Meyer R. (2007 Jul). Surgical treatment of right-sided active infective endocarditis with or without involvement of the left heart: 20-year single center experience. Eur. J. Cardiothorac. Surg..

[34] Pfannmueller B., Kahmann M., Davierwala P., Misfeld M., Bakhtiary F., Binner C. (2017 Dec). Tricuspid Valve Surgery in patients with Isolated Tricuspid Valve Endocarditis: Analysis of Perioperative Parameters and Long-Term Outcomes. Thorac. Cardiovasc. Surg..

[35] Protos A.N., Trivedi J.R., Whited W.M., Rogers M.P., Owolabi U., Grubb K.J. (2018 Sep). Valvectomy Versus Replacement for the Surgical Treatment of Tricuspid Endocarditis. Ann. Thorac. Surg..

[36] Renzulli A., De Feo M., Carozza A., Della Corte A., Gregorio R., Ismeno G. (1999). Surgery for tricuspid valve endocarditis: a selective approach. Heart Vessels.

[37] Siddiqui E., Alviar C.L., Ramachandran A., Flattery E., Bernard S., Xia Y. (2022 Dec). Outcomes after Tricuspid Valve Operations in patients with Drug-Use Infective Endocarditis. Am. J. Cardiol..

[38] Shetty N., Nagpal D., Koivu S., Mrkobrada M. (2016 Feb). Surgical and Medical Management of Isolated Tricuspid Valve Infective Endocarditis in Intravenous Drug users. J. Card. Surg..

[39] Slaughter M.S., Badhwar V., Ising M., Ganzel B.L., Sell-Dottin K., Jawitz O.K. (2021 Apr). Optimum surgical treatment for tricuspid valve infective endocarditis: an analysis of the Society of Thoracic Surgeons national database. J. Thorac. Cardiovasc. Surg..

[40] Thourani V.H., Bonnell L., Wyler von Ballmoos M.C., Mehaffey J.H., Bowdish M., Kurlansky P. (2024 Oct). Outcomes of Isolated Tricuspid Valve Surgery: a Society of Thoracic Surgeons Analysis and Risk Model. Ann. Thorac. Surg..

[41] Huang X., Gu C., Men X., Zhang J., You B., Zhang H. (2014 Apr). Repair of functional tricuspid regurgitation: comparison between suture annuloplasty and rings annuloplasty. Ann. Thorac. Surg..

[42] Amedi A., Onohara D., Xu D., Suresh K.S., Padala M. (2022 Jul). Hemodynamic outcomes after undersizing ring annuloplasty and focal suture annuloplasty for surgical repair of functional tricuspid regurgitation. J. Thorac. Cardiovasc. Surg..

[43] Dreyfus J., Juarez-Casso F., Sala A., Carnero-Alcazar M., Eixerés-Esteve A., Bohbot Y. (2024 Nov 8). Benefit of isolated surgical valve repair or replacement for functional tricuspid regurgitation and long-term outcomes stratified by the TRI-SCORE. Eur. Heart J..

[44] Baudo M., Cuko B., Ternacle J., Magrini E., Busuttil O., Dib N. (2025 Jul). Isolated surgical valve replacement for tricuspid regurgitation: an international multicenter study. Surgery.

[45] Comentale G., Ahmadi-Hadad A., Moldon H.J., Carbone A., Manzo R., Macchio C.C. (2025 Mar 17). Comparative Outcomes of Mitral Valve Repair versus Replacement in Infective Endocarditis: a 16-Year Meta-Analysis of Time-to-Event Data from over 4000 patients. Am. J. Cardiol..

[46] Choi J.W., Jang M.J., Kim K.H., Hwang H.Y. (2018 Apr 1). Repair versus replacement for the surgical correction of tricuspid regurgitation: a meta-analysis. Eur. J. Cardiothorac. Surg..

[47] Viele K. (2023 Jul 20). Allocation in platform trials to maintain comparability across time and eligibility. Stat. Med..

[48] Doenst T., Caldonazo T., Mukharyamov M., Tasoudis P., Kirov H. (2024 Sep 13). Survival Correlates with Regurgitation Degree before and after Invasive Atrioventricular Valve Treatment. Thorac. Cardiovasc. Surg..

[49] Pettersson G.B., Hussain S.T. (2019 Nov). Current AATS guidelines on surgical treatment of infective endocarditis. Ann Cardiothorac Surg..

[50] Truong S., Petersen J., Schmiegelow M.D.S., Due H., Havers-Borgersen E., Smerup M. (2025 Jan). Incidence and factors associated with mitral valve reoperation in patients undergoing surgery for mitral regurgitation: a nationwide cohort study. Int. J. Cardiol..

[51] Akins C.W., Miller D.C., Turina M.I., Kouchoukos N.T., Blackstone E.H., Grunkemeier G.L. (2008 Apr). Guidelines for reporting mortality and morbidity after cardiac valve interventions. Ann. Thorac. Surg..

