# Supplementary material for: Long-term outcomes of surgical repair versus replacement for tricuspid valve endocarditis − A meta-analysis of reconstructed time-to-event data
Source: Int J Cardiol Heart Vasc. 2025 Aug 28;60:101782. doi: 10.1016/j.ijcha.2025.101782 (PMC12410490; doi:10.1016/j.ijcha.2025.101782)
Supplement: Supplementary Data 1 [file mmc1.docx]

**SUPPLEMENTARY APPENDIX**

**TABLE OF CONTENT**

[**Supplementary Methods 1. PRISMA 2020 Main Checklist 3**](#_heading=h.50j4nh1s268i)

[**Supplementary Methods 2. Details of Search Strategy 6**](#_heading=h.alkddzw5z51e)

[**Supplementary Methods 3. Eligibility criteria per included study 7**](#_heading=h.80upss1jok53)

[**Supplementary Methods 4. Endpoint definition per included study 11**](#_heading=h.1jwmvfa1mcw1)

[Supplementary Methods 4A. Early mortality, Post-Operative Stroke and Acute Kidney Injury definition across the included cohorts 11](#_heading=h.f3nldu8jaxvp)

[Supplementary Methods 4B. Post-operative deep wound infection, reoperation and reinfection definition across the included cohorts 14](#_heading=h.jz7stquklml)

[**Supplementary Table 1. Reasons for exclusion in the potentially eligible studies 17**](#_heading=h.qcd3mofaxsj7)

[**Supplementary Table 2. Additional characteristics of the included studies 23**](#_heading=h.rrsmw9rgsd6d)

[Supplementary Table 2A. Demographic patient characteristics of the included studies 23](#_heading=h.zaqnpmhcboky)

[Supplementary Table 2B. Additional demographic patient characteristics of the included studies 26](#_heading=h.xa6w0blbf0va)

[Supplementary Table 2C. TV Repair and Replacement characteristics of the included studies 29](#_heading=h.qo5kvd49lcza)

[**Supplementary Table 3. Indication for surgery per included study 31**](#_heading=h.nztdre2452cb)

[**Supplementary Table 4. Surgery description per included study 35**](#_heading=h.viwpu469vpsp)

[**Supplementary Table 5. Causative microorganisms description per included study 41**](#_heading=h.6qugud53ipsq)

[**Supplementary Table 6. Grading of Recommendations, Assessment, Development and Evaluation (GRADE) 43**](#_heading=h.cdkxgonvwj9e)

[**Supplementary Results 1. Reported and reconstructed Kaplan-Meier curves comparison at the study level 44**](#_heading=h.9e0gwl5pxcpx)

[**Supplementary Figure 1. PRISMA Flowchart 48**](#_heading=h.i58xynkjfkmo)

[Supplementary Figure 2. Test of Proportional Hazards Assumption 49](#_heading=)

[**Supplementary Figure 3. Subanalysis for isolated tricuspid valve cohort 50**](#_heading=h.d7fqfwqqvl9q)

[**Supplementary Figure 4. Forest plot for secondary endpoints 51**](#_heading=h.jtqwbfv4z5g4)

[Supplementary Figure 4A. Forest plot for postoperative deep wound infection 51](#_heading=)

[Supplementary Figure 4B. Forest plot for permanent pacemaker implantation 51](#_heading=h.1lbkkxn8la4j)

[**Supplementary Figure 5. Sensitivity Analysis 52**](#_heading=h.eit20x9wxe3u)

[Supplementary Figure 5A. Leave-one-out for long-term all-cause mortality endpoint 52](#_heading=h.i8ro8gs4vpwi)

[Supplementary Figure 5B. Leave-one-out for any reoperation endpoint 52](#_heading=h.50q5m1hi13ob)

[Supplementary Figure 5C. Leave-one-out for reinfection endpoint 53](#_heading=h.n494c972urd4)

[Supplementary Figure 5D. Leave-one-out for early mortality endpoint 53](#_heading=h.4xr7vqbktkol)

[Supplementary Figure 5E. Leave-one-out for postoperative stroke 54](#_heading=h.3m9ixv6aky2c)

[Supplementary Figure 5F. Leave-one-out for acute kidney injury 54](#_heading=h.6wif129hb45h)

[Supplementary Figure 5G. Leave-one-out for postoperative deep wound infection 54](#_heading=h.3nvrlj4bwcje)

[Supplementary Figure 5H. Leave-one-out for permanent pacemaker implantation 56](#_heading=)

[Supplementary Figure 6. Post hoc Subgroup Analyses 57](#_heading=)

[Supplementary Figure 6A. Post hoc subgroup analysis for Risk of Bias 57](#_heading=)

[Supplementary Figure 6B. Post hoc subgroup analysis for bioprosthetic versus mechanical valves 57](#_heading=)

[Supplementary Figure 7. Quality assessment using Risk Of Bias In Non-randomized Studies (ROBINS-I) 58](#_heading=)

[Supplementary Figure 7A. "Traffic light" plot of the domain-level judgments for each study 58](#_heading=h.95aqa2yu19j9)

[Supplementary Figure 7B. Summary of overall weighted bar plot of risk-of-bias judgments within each bias domain. 59](#_heading=)

[**Supplementary Figure 8. Funnel plot and Egger’s test 60**](#_heading=h.gwcwuj43t40b)

[Supplementary Figure 8A. Funnel plot for long-term all-cause mortality endpoint 60](#_heading=h.swl9q03ifn91)

[Supplementary Figure 8B. Funnel plot for any reoperation endpoint 60](#_heading=h.eqtu6ru3ssdr)

[Supplementary Figure 8C. Funnel plot for reinfection endpoint 61](#_heading=h.f6iocactlaem)

[Supplementary Figure 8D. Funnel plot for early mortality endpoint 61](#_heading=h.ioa7ml928b06)

[Supplementary Figure 8E. Funnel plot for postoperative stroke endpoint 62](#_heading=h.fpsm4k9vqy7s)

[Supplementary Figure 8F. Funnel plot for acute kidney injury endpoint 62](#_heading=h.qex486x59m2e)

[Supplementary Figure 8G. Funnel plot for postoperative deep wound infection endpoint 63](#_heading=h.3ot4mn1j0j7f)

[Supplementary Figure 8H. Funnel plot for permanent pacemaker implantation 63](#_heading=h.qehgfdvnb956)

[**References 64**](#_heading=h.j387enx1cyit)

#

# **Supplementary Methods 1. PRISMA 2020 Main Checklist**

| **Topic** | **No.** | **Item** | **Location where item is reported** |
| --- | --- | --- | --- |
| **TITLE** |  |  |  |
| **Title** | 1 | Identify the report as a systematic review. | Pg .1; MS |
| **ABSTRACT** |  |  |  |
| **Abstract** | 2 | See the PRISMA 2020 for Abstracts checklist | Pg. 2; MS |
| **INTRODUCTION** |  |  |  |
| **Rationale** | 3 | Describe the rationale for the review in the context of existing knowledge. | Pg. 4; MS |
| **Objectives** | 4 | Provide an explicit statement of the objective(s) or question(s) the review addresses. | Pg. 4; MS |
| **METHODS** |  |  |  |
| **Eligibility criteria** | 5 | Specify the inclusion and exclusion criteria for the review and how studies were grouped for the syntheses. | Pg. 5; MS and SM3 |
| **Information sources** | 6 | Specify all databases, registers, websites, organizations, reference lists and other sources searched or consulted to identify studies. Specify the date when each source was last searched or consulted. | Pg. 5; MS |
| **Search strategy** | 7 | Present the full search strategies for all databases, registers, and websites, including any filters and limits used. | Pg. 5-6; MS and SM2 |
| **Selection process** | 8 | Specify the methods used to decide whether a study met the inclusion criteria of the review, including how many reviewers screened each record and each report retrieved, whether they worked independently, and if applicable, details of automation tools used in the process. | Pg. 5-6; MS and ST1, ST2, ST3 and ST4 |
| **Data collection process** | 9 | Specify the methods used to collect data from reports, including how many reviewers collected data from each report, whether they worked independently, any processes for obtaining or confirming data from study investigators, and if applicable, details of automation tools used in the process. | Pg. 5-6; MS |
| **Data items** | 10a | List and define all outcomes for which data were sought. Specify whether all results that were compatible with each outcome domain in each study were sought (e.g., for all measures, time points, analyses), and if not, the methods used to decide which results to collect. | Pg. 5-6; MS and SM4 |
|  | 10b | List and define all other variables for which data were sought (e.g., participant and intervention characteristics, funding sources). Describe any assumptions made about any missing or unclear information. | Pg.5-6; MS |
| **Study risk of bias assessment** | 11 | Specify the methods used to assess risk of bias in the included studies, including details of the tool(s) used, how many reviewers assessed each study and whether they worked independently, and if applicable, details of automation tools used in the process. | Pg. 5-7; MS |
| **Effect measures** | 12 | Specify for each outcome the effect measure(s) (e.g., risk ratio, mean difference) used in the synthesis or presentation of results. | Pg. 5-7; MS |
| **Synthesis methods** | 13a | Describe the processes used to decide which studies were eligible for each synthesis (e.g., tabulating the study intervention characteristics and comparing against the planned groups for each synthesis (item 5)). | Table 1 |
|  | 13b | Describe any methods required to prepare the data for presentation or synthesis, such as handling of missing summary statistics, or data conversions. | Pg. 5-7; MS |
|  | 13c | Describe any methods used to tabulate or visually display results of individual studies and syntheses. | Pg. 5-7; MS |
|  | 13d | Describe any methods used to synthesize results and provide a rationale for the choice(s). If meta-analysis was performed, describe the model(s), method(s) to identify the presence and extent of statistical heterogeneity, and software package(s) used. | Pg. 5-7; MS |
|  | 13e | Describe any methods used to explore possible causes of heterogeneity among study results (e.g., subgroup analysis, meta-regression). | Pg. 5-7; MS |
|  | 13f | Describe any sensitivity analyses conducted to assess robustness of the synthesized results. | Pg. 5-7; MS |
| **Reporting bias assessment** | 14 | Describe any methods used to assess risk of bias due to missing results in a synthesis (arising from reporting biases). | Pg. 5-7; MS |
| **Certainty assessment** | 15 | Describe any methods used to assess certainty (or confidence) in the body of evidence for an outcome. | Pg. 6; MS |
| **RESULTS** |  |  |  |
| **Study selection** | 16a | Describe the results of the search and selection process, from the number of records identified in the search to the number of studies included in the review, ideally using a flow diagram. | Figure 1 |
|  | 16b | Cite studies that might appear to meet the inclusion criteria, but which were excluded, and explain why they were excluded. | ST1 |
| **Study characteristics** | 17 | Cite each included study and present its characteristics. | Pg. 8-9; MS |
| **Risk of bias in studies** | 18 | Present assessments of risk of bias for each included study. | Pg. 8-9; MS and ST6, SF5 |
| **Results of individual studies** | 19 | For all outcomes, present, for each study: (a) summary statistics for each group (where appropriate) and (b) an effect estimates and its precision (e.g., confidence/credible interval), ideally using structured tables or plots. | Fig. 2, 3, 4; MS |
| **Results of syntheses** | 20a | For each synthesis, briefly summarize the characteristics and risk of bias among contributing studies. | Pg. 8-9; MS |
|  | 20b | Present results of all statistical syntheses conducted. If meta-analysis was done, present for each the summary estimate and its precision (e.g., confidence/credible interval) and measures of statistical heterogeneity. If comparing groups, describe the direction of the effect. | Pg. 8-9; MS and SR1, SF1, SF2, SF3. SF4 |
|  | 20c | Present results of all investigations of possible causes of heterogeneity among study results. | Pg. 8-9; MS |
|  | 20d | Present results of all sensitivity analyses conducted to assess the robustness of the synthesized results. | Pg. 8-9; MS |
| **Reporting biases** | 21 | Present assessments of risk of bias due to missing results (arising from reporting biases) for each synthesis assessed. | Pg. 8-9; MS |
| **Certainty of evidence** | 22 | Present assessments of certainty (or confidence) in the body of evidence for each outcome assessed. | Pg. 9, MS |
| **DISCUSSION** |  |  |  |
| **Discussion** | 23a | Provide a general interpretation of the results in the context of other evidence. | Pg. 10-12; MS |
|  | 23b | Discuss any limitations of the evidence included in the review. | Pg. 10-12; MS |
|  | 23c | Discuss any limitations of the review processes used. | Pg. 10-12; MS |
|  | 23d | Discuss implications of the results for practice, policy, and future research. | Pg. 10-12; MS |
| **OTHER INFORMATION** |  |  |  |
| **Registration and protocol** | 24a | Provide registration information for the review, including register name and registration number, or state that the review was not registered. | CRD420251015463 |
|  | 24b | Indicate where the review protocol can be accessed, or state that a protocol was not prepared. | <https://www.crd.york.ac.uk/PROSPERO/view/CRD420251015463> |
|  | 24c | Describe and explain any amendments to information provided at registration or in the protocol. | NA |
| **Support** | 25 | Describe sources of financial or non-financial support for the review, and the role of the funders or sponsors in the review. | None |
| **Competing interests** | 26 | Declare any competing interests of review authors. | Pg. 1; MS |
| **Availability of data, code and other materials** | 27 | Report which of the following are publicly available and where they can be found template data collection forms; data extracted from included studies; data used for all analyses; analytic code; any other materials used in the review. | NR |

^Abbreviations: MS, manuscript; SR, supplementary results;^

# **Supplementary Methods 2. Details of Search Strategy**

| **Search Strategy for each database** | |
| --- | --- |
| **MEDLINE**  **(Pubmed)** | ("Tricuspid Valve"[Mesh] OR tricuspid OR TV) AND ((Surgical OR surgery) OR ((Repair OR Reconstruction) OR (Replace* OR replacement))) AND "Endocarditis"[Mesh] |
| **EMBASE** | ('tricuspid valve'/exp OR 'tricuspid' OR 'tv') AND (('surgical' OR 'surgery') OR (('repair' OR 'reconstruction') OR ('replace*' OR 'replacement'))) AND 'endocarditis'/exp |
| **CENTRAL**  **(Cochrane)** | ("Tricuspid Valve" OR tricuspid OR TV) AND ((Surgical OR surgery) OR ((Repair OR Reconstruction) OR (Replace* OR replacement))) AND "Endocarditis" |
| **LILACS** | (tricuspid OR "valva tricúspide" OR "válvula tricúspide" OR "tricuspid valve" OR TV) AND (endocarditis OR endocardite) AND (surgical OR surgery OR cirurgia OR repair OR reconstrução OR reconstruir OR replace OR replacement OR substituição OR substituir) |
| **Clinical Trials.gov** | (tricuspid OR "tricuspid valve" OR TV) AND (surgery OR surgical OR repair OR reconstruction OR replace OR replacement) AND endocarditis |

#

# **Supplementary Methods 3. Eligibility criteria per included study**

| **Study and Year** | **Eligibility Criteria** |
| --- | --- |
| **Baraki, 2010[25]** | “From June 1996 to September 2012 a total of 637 patients underwent TV surgery at Hannover Medical School. Out of this cohort, we retrospectively analyzed the data of 33 patients, who underwent isolated TV repair or replacement due to endocarditis. Data were extracted from patient charts recorded in the hospital’s computer database.”  *MS; Page 1, Methods section* |
| **Brescia, 2022[26]** | “The study population included consecutive adults undergoing primary valve repair or replacement for mitral (n = 260) or tricuspid (n = 71) valve endocarditis at a single academic center between July 1992 and December 2018 (n = 331). Patients with aortic, pulmonic, or both left and right sided endocarditis were excluded, while those undergoing concomitant coronary artery bypass grafting (CABG), anti-arrhythmia procedures, and with previous cardiac surgery unrelated to the mitral or tricuspid valve were included.”  *MS; Page 2, Patients Population section* |
| **Dawood, 2015[27]** | “A total of 322 patients underwent an operation for IE at the University of Maryland Medical Center between January 2002 and December 2012. Of these, 63 had TV operations. Three patients with rheumatic valve disease and four with functional tricuspid disease were excluded.  This yielded a cohort of 56 consecutive patients for analysis with active or treated TVIE, as depicted in Figure 1. Patient data were gathered from The Society of Thoracic Surgeons (STS) local database and supplemented with record reviews. All patients included in this study met the modified Duke criteria for IE.”  *MS; Page 1, Material and Methods section* |
| **Dimauro, 2022[28]** | “From 1983 to 2018, isolated acute TVIE was surgically treated in 157 out of 4069 (3.8%) patients, with 142 (90%) cases of native tricuspid regurgitation (TR), 7 (5%) of stenosis and regurgitation (TSR), and 8 (5%) of tricuspid prosthesis endocarditis.11 Excluding the latter group, 149 patients with native TVIE were included, 77 had valve repair (Repair Group) and 72 replacement (Replacement Group). No patients underwent valvectomy in our experience.”  *MS; Page 2; Methods section* |
| **Dzilic, 2022[29]** | “This study presents a retrospective analysis of all consecutive  patients who underwent tricuspid valve (TV) surgery due to isolated  TVE at the German Heart Centre Munich between February 2001  and June 2021. Patients' data were identified from our internal  clinical database. All medical reports, including operative protocols,  in‐hospital and outpatient notes were reviewed. Patients with  concomitant procedures were excluded. The study was approved  by the Institutional Review Board of the Technical University of  Munich (Number: 356/21).”  *MS; Page 2; Methods section* |
| **Gaca, 2013[30]** | “The STS ACSD was established in 1989 and includes data  from more than 4 million cardiac surgical operations, currently from more than 90% of cardiac surgical centers in North America. Patient variables are entered using uniform definitions. Data storage and analysis are performed at the Duke Clinical Research Institute. The population for this study consisted of all patients with the diagnosis of IE who underwent surgery on the TV at STS participating hospitals between 2002 and 2009. This era was selected because it represented a large patient population with the most complete data available in the STS ACSD. Data were collected using the forms provided at the STS website (http://www.sts.org/doc/8428). Inclusion criteria included any isolated TV operation and a diagnosis of IE in patients between the ages of 18 and 90 years of age. Any concomitant operations for arrhythmia or ventricular septal defect also were included. Exclusion  criteria were missing data on sex, status of surgery, cardiogenic shock, endocarditis type, and prior valve operation.”  *MS; Page 1-2, Dataset section* |
| **Gottardi, 2007[31]** | “Between October 1997 and July 2004, 22 consecutive patients with active tricuspid valve endocarditis underwent surgical treatment at our institution. In 1997 we started to aggressively use reconstructive techniques rather than valve replacement in all patients presenting with active infective tricuspid valve endocarditis. The aim of this retrospective study was to analyze our results after tricuspid valve reconstruction for active infective tricuspid endocarditis during at least a midterm follow-up period. The ethics committee approved the study  and waived the need for patient consent.”  *MS; Page 2, Material and Methods section* |
| **Jawad, 2020[32]** | “This retrospective, observational, nonrandomized study included all patients presenting with isolated tricuspid valve endocarditis due to IV drug abuse and who had undergone tricuspid valve repair or replacement between January 2014 and January 2016. The study protocol was reviewed and approved by the Department of Cardiothoracic Surgery Ethical Committee and the Faculty of Medicine Ain Shams Research Institute, by which the patient’s consent was waived, enrolling only those who sought medical attention voluntarily. The exclusion criteria were age younger than 18 years  or older than 60 years, patients referred from correctional facilities, patients who underwent previous cardiac interventions, and patients with preoperative hemoglobin of less than 8 g/dL, white blood cells exceeding 16,000 cells/mL, and platelets count of less than 90,000/mL”  *MS; Page 1-2, Patients and Methods section* |
| **Lee, 2020[9]** | “Patients who received TV surgery between 1 January 2000 and 31 December 2013 were identified using both International Classification of Disease, Ninth Revision, Clinical Modification (ICD-9-CM) procedure codes (i.e. 35.27 and 35.28 for TV replacement and 35.14 for TV repair) and Taiwan NHI reimbursement codes (68015, 68016, 68017 and 68018). Of these, patients diagnosed as having IE before or during the index hospitalization were identified using ICD-9-CM diagnosis codes (421.0, 421.1 and 421.9) and included in our study. Index hospitalization was defined as the hospitalization during which TV surgery was performed.  Patients were excluded if their age was <20 years or both replacement and repair were performed during the same admission.”  *MS; Page 2, Study population section* |
| **Musci, 2007[33]** | Retrospective cohort comparing surgical approach for isolated right-sided versus concomitant left-sided active infective endocarditis. A subgroup for tricuspid repair versus tricuspid replacement is reported in the text. **No specific eligibility criteria reported**. |
| **Pfannmueller, 2015[34]** | Retrospective cohort comparing the causes, perioperative, long term endpoints in patients with tricuspid infective endocarditis undergoing tricuspid valve surgery. A subgroup for tricuspid repair versus tricuspid replacement is reported in the text. **No specific eligibility criteria reported.** |
| **Protos, 2018[35]** | “A single-center cardiac surgery database was evaluated after Institutional Review Board approval to identify patients aged 18 years or older undergoing tricuspid valve endocarditis operations between January 2012 and December 2016. Patients with a concomitant operation were also included in the final data set. The patients were divided into three groups by the type of tricuspid operation they received. Patients who received tricuspid valve repair were classified in the repair group, those receiving a tricuspid valve excision were classified in the valvectomy group, and patients receiving tricuspid valve replacement were classified in the replacement group.”  *MS; Page 2, Patients and Methods section* |
| **Renzulli, 1999[36]** | NR |
| **Siddiqui, 2022[37]** | “We identified all hospitalizations of patients from 2002 to 2019 who were admitted with IE and then selected patients with co-morbid DU using the Elixhauser co-morbidity software tool provided by the Healthcare Cost and Utilization Project (Rockville, Maryland). Patients who were found to have PE on admission were selected for the analysis as this was a guideline indication for TV operation. Procedural codes were used to identify the cohort of patients who underwent TV operations, including both repair and replacement.”  *MS; Page 2; Methods Section* |
| **Shetty, 2015[38]** | “Patients admitted with native tricuspid valve infective endocarditis as proven by either transthoracic or transesophageal echocardiography were included in the study. Patients were excluded if they were under  18 years of age, had a noninfective cause of endocarditis, had suspected endocarditis without echocardiographic evidence of a vegetation, had prosthetic valve endocarditis, or had involvement of the aortic valve, mitral valve or an intracardiac device.”  *MS; Page 2, Eligibility section* |
| **Slaughter, 2021[39]** | “The STS ACSD access and publication committee approved our proposal to access data on adult patients undergoing tricuspid valve surgery for infective endocarditis between years 2011 and 2016. Tricuspid surgery patients undergoing concomitant surgeries, reoperations as well as with severe aortic or mitral insufficiency were excluded. Patients without history of TV endocarditis or IVDA and with history of aortic and mitral valve endocarditis (but not receiving surgery) were also excluded.”  *MS; Page 2, Study population section* |
| **Thourani, 2021[40]** | “All adult patients aged 18 years and older who underwent isolated TVr or TVR between July 2017 and June 2023 were identified from the STS Adult Cardiac Surgery Database (ACSD; data versions 2.9 and 4.20.2). The STS ACSD includes data from more than 96% of adult cardiac surgical operations performed in the United States. TVr cases included patients who underwent annuloplasty only, reconstruction only, or annuloplasty combined with reconstruction. Tricuspid valvectomy, interventions on previous prosthetic valves, pannus thrombus removal procedures, and transcatheter interventions were excluded. The study was limited to isolated TV surgery; TVr procedures performed concomitantly with MV repairs or replacements were also excluded and remain part of current STS MV risk models. Included concomitant procedures were ablation, left atrial appendage obliteration, and atrial septal defect or patent foramen ovale repairs. Finally, cases in patients with unknown 30-da vital status were excluded.”  *MS; Page 2, Data Source and patient population section* |
| **Witten, 2018[10]** | “From January 1, 2002, to January 1, 2015, 1292 consecutive adults underwent surgery for IE at Cleveland Clinic, of whom 134 (10%) had right-sided IE. All met modified Duke criteria for definite IE.”  *MS; Page 2, Data Source and patient population section* |
| **Xie, 2023[11]** | “In this retrospective study, we examined fifty-six patients with TIE who were surgically treated at our institution from January 2006 to August 2019. These patients were divided into the TVP group (n=23) and the TVR group (n=33). All patients were diagnosed according to the modified Duke criteria, or TIE was confirmed by post-operative pathology. Preoperative transthoracic echocardiography showed tricuspid valve vegetation in all cases”  *MS; Page 2, Patient section* |

*Abbrevations: MS: Main manuscript and NR: Not reported*

# **Supplementary Methods 4. Endpoint definition per included study**

## Supplementary Methods 4A. Early mortality, Post-Operative Stroke and Acute Kidney Injury definition across the included cohorts

| **Study**  **and**  **Year** | **Early mortality** | **Post-Operative Stroke** | **Acute Kidney Injury** |
| --- | --- | --- | --- |
| **Baraki,**  **2013[25]** | NR | NR | NR |
| **Brescia,**  **2021[26]** | NR | NR | NR |
| **Dawood,**  **2015[27]** | NR | "Any confirmed neurological deficit of abrupt onset caused by a disturbance in blood supply to the brain that was confirmed on imaging or did not resolve within 24 hours" | NR |
| **Di Mauro,**  **2022[28]** | “Defined as death within 30 days after  surgery due to any cause”**^15^** | NR | NR |
| **Dzallic,**  **2020[29]** | NR | NR | NR |
| **Gaca,**  **2013[30]** | "(1) all deaths during the  hospitalization in which the operation was performed, even if after 30 days (including patients transferred to other acute care facilities)  (2) all deaths, regardless of cause, occurring after discharge from the hospital, but before the end of the thirtieth postoperative day." | "Any confirmed neurological deficit of abrupt onset caused by a disturbance in blood supply to the brain that was confirmed on imaging or did not resolve within 24 hours" | "Acute renal failure or worsening renal function resulting in  ONE OR BOTH of the following:  1. Increase in serum creatinine level 3.0 x  greater than baseline, or serum creatinine level ≥4 mg/dL , Acute rise must be at least 0.5 mg/dl  2. A new requirement for dialysis postoperatively" |
| **Gottardi,**  **2007[31]** | "Intraoperative death" | NR | NR |
| **Jawad,**  **2020[32]** | "Death at the same hospital stay" | NR | "New transient renal impairment" |
| **Lee*,**  **2020[9]** | NR | NR | "De novo dialysis" |
| **Musci,**  **2007[33]** | NR | NR | NR |
| **Pfannmueller,**  **2015[34]** | NR | NR | NR |
| **Protos,**  **2018[35]** | "All deaths, regardless of cause, occurring after  discharge from the hospital, but before the end of the  thirtieth postoperative day" | NR | NR |
| **Renzulli,**  **1999[36]** | NR | NR | NR |
| **Siddiqui,**  **2022[37]** | NR | NR | NR |
| **Shetty,**  **2017[38]** | NR | NR | NR |
| **Slaughter,**  **2019[39]** | "In-hospital or 30-day post-operative mortality" | NR | NR |
| **Thourani,**  **2024[40]** | "Defined as a death during the index hospitalization for surgery or within 30 days of the procedure" | "Defined as an acute episode of focal or global neurologic dysfunction caused by brain, spinal cord, or retinal vascular injury as a result of hemorrhage or infarction in which the neurologic dysfunction lasts for more than 24 hours" | "Defined as new requirement for dialysis or meeting the  Risk, Injury, Failure, Loss of kidney function,  and End-stage kidney disease (RIFLE)  criteria based on creatinine levels or glomerular filtration rate" |
| **Witten,**  **2018[10]** | NR | NR | NR |
| **Xie,**  **2023[11]** | NR | NR | NR |

##

## Supplementary Methods 4B. Post-operative deep wound infection, reoperation and reinfection definition across the included cohorts

| **Study**  **and**  **Year** | **Post-operative**  **deep wound infection** | **Reoperation** | **Reinfection** |
| --- | --- | --- | --- |
| **Baraki,**  **2013[25]** | NR | "Any operation that repairs, alters or  replaces a previously operated valve" [] | NR |
| **Brescia,**  **2021[26]** | NR | "Any subsequent intervention on  the endocarditis-affected valve" | NR |
| **Dawood,**  **2015[27]** | "Diagnosed within 30 days of  the OR date or at any time  during the initial hospitalization" | NR | "Reinfection of the endocardial surface  of the heart, which may involve one or  more heart valves (native or prosthetic)  or septaldefects or  prosthetic patch material  placed at previous surgery."  Dukes Criteria |
| **Di Mauro,**  **2022[28]** | NR | NR | NR |
| **Dzilic,**  **2022[29]** | NR | NR | NR |
| **Gaca,**  **2013[30]** | NR | "Returned to the operating room for prosthetic or  native valve dysfunction. Dysfunction  may be structural and/or non-structural failure.  Dysfunction may be of prosthesis,  a progressive native disease process,  or an acute event process that disrupts valve  function and creates either clinical  compromising insufficiency/regurgitation  or valve orifice narrowing." | "Reinfection of the endocardial surface  of the heart, which may involve one or  more heart valves (native or prosthetic)  or septal defects or  prosthetic patch material  placed at previous surgery."  Dukes Criteria |
| **Gottardi,**  **2007[31]** | NR | NR | NR |
| **Jawad,**  **2020[32]** | NR | NR | NR |
| **Lee*,**  **2020[9]** | NR | NR | NR |
| **Musci,**  **2007[33]** | NR | NR | NR |
| **Pfannmueller,**  **2015[34]** | NR | NR | NR |
| **Protos,**  **2018[35]** | NR | NR | "Criteria for return to the operating room secondary to  bleeding varied by surgeon, but all included hemody-  namic instability as an important decision-making factor." |
| **Renzulli, 1999[36]** | NR | NR | NR |
| **Siddiqui, 2022[37]** | NR | NR | NR |
| **Shetty,**  **2017[38]** | NR | NR | NR |
| **Slaughter,**  **2019[39]** | NR | NR | NR |
| **Thourani,**  **2024[40]** | NR | "Reoperation for bleeding,  tamponade, or any cardiac reason" | NR |
| **Witten,**  **2018[10]** | NR | NR | "Met the modified Duke criteria for IE subsequent to discharge after the index  hospitalization. Recurrence was categorized as IE  ‘‘relapse’’ or ‘‘reinfection’’ based on timing and microbiology. If microbiology  was consistent between episodes and recurrence occurred within 6 months  after index surgery, it was considered disease relapse; if microbiology was  disparate between episodes, or recurrence occurred  more than 6 months after index operation,  it was classified as reinfection." |
| **Xie,**  **2023[11]** | NR | NR | "The diagnoses of IE relapse and IE reinfection were  based on the modified Duke criteria. Relapse was defined  as IE caused by any microbial infection within 6 months  post-surgery. Reinfection was defined as an infection that  was caused by a different microorganism from the primary  infection or an infection that occurred more than 6  months after the initial infection" |

Abbreviations: NR: Not Reported

# **Supplementary Table 1. Reasons for exclusion in the potentially eligible studies**

| **Study and Year** | **Exclusion Criteria** |
| --- | --- |
| **Tarmiz, A., 2010** | Does not report endpoints stratified by tricuspid repair and replacement |
| **Radmehr, 2009** | Wrong study design: Case Series |
| **Kolek, 2009** | Does not report endpoints stratified by tricuspid repair and replacement |
| **Capoun, 2010** | Wrong study design: Case Series |
| **Morokuma, 2010** | Wrong study design: Case Series |
| **Turley, 1989** | Wrong study design: Case Series |
| **Jiang, 2011** | No outcome of interest |
| **Xu, 1998** | A retrospective study evaluating the outcomes for surgery repair in patients with  endocarditis.  Does not include stratified repair versus replacement subgroups for comparison |
| **Lange, 1996** | Case Series |
| **Lukacs, 1993** | Case series evaluating the outcomes for surgical repair in patients with  endocarditis |
| **Misfeld, 2014** | A retrospective cohort evaluating the surgical outcomes for patients with  embolisms versus those without  Does not include stratified repair versus replacement subgroups for comparison |
| **Toyoda, 2014** | Wrong study design: Case Report |
| **Altaani, 2013** | A retrospective cohort evaluating the surgical outcomes for tricuspid valve replacement  Does not include stratified repair versus replacement subgroups for comparison |
| **Nayak, 2011** | A retrospective cohort evaluating the surgical outcomes for surgery in patients with  endocarditis  Does not include stratified repair versus replacement subgroups for comparison |
| **Yie, 2010** | Wrong study design: Letter to the editor |
| **Kilic, 2020** | A retrospective cohort comparing intravenous drug use versus no use in  patients with endocarditis undergoing surgery  Does not include stratified repair versus replacement subgroups for comparison |
| **Carozza, 2001** | Case Series of patients undergoing tricuspid valve repair for endocarditis.  Does not have the tricuspid replacement subgroup |
| **Allen, 1991** | Case Series of patients undergoing tricuspid valve repair for endocarditis.  Does not have the tricuspid replacement subgroup |
| **Veen, 2019** | A retrospective cohort evaluating male versus female differences in  patients undergoing isolated tricuspid surgery  Does not include stratified repair versus replacement subgroups for comparison |
| **Na, 2019** | A research letter and  retrospective cohort evaluating the predictors of isolated tricuspid valve surgery in endocarditis.  Does not include stratified repair versus replacement subgroups for comparison |
| **Wallen, 2018** | A retrospective cohort evaluating the predictors of isolated tricuspid valve surgery in  the opioid epidemic.  Does not include stratified repair versus replacement subgroups for comparison |
| **Said, 2017** | A retrospective cohort evaluating the predictors of surgery in infective endocarditis  patients with endocarditis  Does not include stratified repair versus replacement subgroups for comparison |
| **Singer, 2017** | A retrospective cohort evaluating the predictors of isolated tricuspid valve surgery in  patients with endocarditis  Does not include stratified repair versus replacement subgroups for comparison |
| **Farag, 2017** | A retrospective cohort comparing the usage of repair versus  replacement in isolated tricuspid surgery  Does not include stratified endorcarditis subgroups for comparison |
| **Abacılar, 2019** | A retrospective cohort comparing the usage of repair versus  replacement in right hearted endocarditis.  Does not include a tricuspid repair and replacement subgroups for comparison |
| **Lorenz, 2024** | Wrong study design: Case Series |
| **Shahmohamadi, 2023** | A retrospective cohort comparing the usage of mechanical valve versus  biological valve in tricuspid valve replacement  Does not include a tricuspid repair subgroup for comparison |
| **Nuzhdin, 2022** | A retrospective cohort evaluating early outcomes of tricuspid valve replacement by  mitral homograft.  Does not include a tricuspid repair subgroup for comparison |
| **Ricci, 2009** | A retrospective study evaluating the Beating-Heart Technique in aortic valve  endocarditis  Does not stratify into tricuspid repair versus replacement with endocarditis subgroups |
| **Aagaard, 2001** | Do not report tricuspid valve repair versus replacement |
| **Lupinetti, 1991** | Retrospective study compared allografts and prosthetic valve in aortic  endocarditis  report tricuspid valve replacement in patients with endocarditis |
| **Gerdisch, 2014** | Evaluates the outcomes of extracellular matrix cylinder reconstruction technique.  Does not compare tricuspid repair versus reconstruction |
| **Vohra, 2014** | Evaluates the outcomes of concomitant aortic and mitral valve repair, but do not  report tricuspid valve replacement in patients with endocarditis |
| **Javadzadegan, 2013** | Has case series design and do not report tricuspid valve replacement |
| **Heinz, 2013** | A retrospective study evaluating the surgical outcomes of Freestyle  aortic root replacement.  Does not provide a tricuspid valve replacement subgroup |
| **Akinosoglou, 2012** | Wrong publication type(review) |
| **Shrestha, 2010** | Maybe, they evaluate the patients with repair versus replacement and we identify which had endocarditis repair versus replacement.  Does not provide a tricuspid valve replacement subgroup |
| **Tarola, 2015** | A retrospective study describing and reporting the outcomes of  tricuspid valve repair in endocarditis.  Does not stratify into tricuspid repair versus replacement with endocarditis subgroups |
| **de Kerchove, 2007** | A retrospective study of the clinical outcome of mitral valve repair  Does not stratify into tricuspid repair versus replacement with endocarditis subgroups |
| **Tamer, 2019** | Abstract from Congress |
| **Poirier, 1998** | A retrospective study of the clinical outcome of the Carpentier-Edwards  Pericardial Bioprosthesis  Does not stratify into tricuspid repair versus replacement with endocarditis subgroups |
| **Chang, 2016** | A retrospective study evaluating the tricuspid valve repair versus replacement in  patients with endocarditis  Does not stratify into tricuspid repair versus replacement with endocarditis subgroups |
| **Oliver, 2022** | A retrospective study evaluating the mitral valve repair versus replacement in  patients with endocarditis  Does not stratify into tricuspid repair versus replacement with endocarditis subgroups |
| **Mohamed, 2021** | A retrospective study evaluating the clinical outcomes to isolated tricuspid valve  reoperation.  Does not stratify into tricuspid repair versus replacement with endocarditis subgroups |
| **Okada, 2021** | A retrospective study evaluating the mitral valve surgery in patients with  active versus endocarditis healed in those who  had previous cardiac surgery versus no surgery  Does not stratify into tricuspid repair versus replacement subgroups |
| **Rodríguez-Capitán, 2020** | Retrospective study evaluating the clinical outcome of tricuspid surgery in those who  had previous cardiac surgery versus no surgery  Does not stratify into tricuspid repair versus replacement subgroups |
| **Mori, 2020** | Retrospective study evaluating the non-opioid  versus opioid use disorder with infective endocarditis in  patients with invasive infective endocarditis with aortomitral involvement.  Does not stratify into tricuspid repair versus replacement subgroups |
| **Brescia, 2019** | Overlapping with Brescia, 2022 |
| **Navia, 2019** | Retrospective study evaluating the Comando versus the Hemicomando technique in  patients with invasive infective endocarditis with aortomitral involvement.  Does not stratify into tricuspid repair versus replacement subgroups |
| **Arafat, 2024** | A retrospective cohort comparing isolated versus concomitant tricuspid surgery.  Does not stratify into patients with infective endocarditis and repair versus replacement  subgroups and their respective endpoints |
| **Rufa, 2023** | A prospective study evaluating the incidence of surgical endpoints in patients  with valve surgery and infective endocarditis using PremiCon suture material.  Does not stratify patients undergoing tricuspid surgical repair versus replacement  and their respective endpoints |
| **Jussli-Melchers, 2022** | Retrospective study evaluating the different clinical endpoints between elderly and  younger patients undergoing valve surgery for infective endocarditis.  Does not report endpoints stratified for repair and replacement |
| **Yousif, 2022** | A retrospective study focusing on the outcome of cryopreserved allografts for aortic  aortic root reconstruction, mentions concomitant tricuspid repair and replacement.  However, does not stratify for tricuspid repair versus replacement endpoints |
| **Mayer, 2012** | A retrospective study focused on aortic repair versus replacement in patients with  Aortic Valve Infective Endocarditis. The cohort with concomitant tricuspid surgery  only had surgical repair; yet this subgroup did not have stratified endpoints |
| **Manne, 2012** | A retrospective study focused on native valves versus prosthetic valves  Does not report endpoints stratified by tricuspid repair and replacement |
| **Hilbig. 2020** | Does not report endpoints stratified by tricuspid repair and replacement |
| **Veve, 2021** | Does not report endpoints stratified by tricuspid repair and replacement |
| **Kassabi, 2022** | Does not report endocarditis group |
| **Di Mauro, 2023** | Does not report endpoints stratified by tricuspid repair and replacement  Overlapping |
| **Hill, 2019** | Does not report endpoints stratified by tricuspid repair and replacement |
| **Nawfor, 2021** | Does not report endpoints stratified by tricuspid repair and replacement |
| **Spiegelstein, 2014** | Abstract from Congress |
| **Ballazhi, 2014** | Abstract from Congress |
| **Feikel. 2014** | Abstract from Congress |
| **Faeber, 2013** | Abstract from Congress |
| **Wang, 2013** | Abstract from Congress |
| **Llagunes, 2012** | Abstract from Congress |
| **Miao, 2012** | Abstract from Congress |
| **Kamiya, 2012** | Abstract from Congress |
| **Baraki, 2011** | Abstract from Congress |
| **Kovalev, 2011** | Abstract from Congress |
| **Jerbi, 2011** | Abstract from Congress |
| **Musci, 2013** | Abstract from Congress |
| **Sharma, 2010** | Abstract from Congress |
| **Di Mauro, 2018** | Abstract from Congress |
| **Alqahtani, 2018** | Abstract from Congress |
| **Nöbauer, 2018** | Abstract from Congress |
| **Wang, 2018** | Abstract from Congress |
| **Na, 2017** | Abstract from Congress |
| **Chandrashekar, 2017** | Abstract from Congress |
| **Zack, 2017** | Abstract from Congress |
| **Bansai, 2021** | Abstract from Congress |
| **Mleyhi, 2021** | Abstract from Congress |
| **Farber, 2021** | Abstract from Congress |
| **Papadopoulos, 2021** | Abstract from Congress |
| **Peerbhai, 2020** | Abstract from Congress |
| **Doǧan, 2020** | Abstract from Congress |
| **Demal, 2023** | Abstract from Congress |
| **Carrel, 1993** | Abstract from Congress |
| **Ranking, 2012** | Abstract from Congress |
| **Heinz, 2013** | Abstract from Congress |
| **Günzinger, 2012** | Abstract from Congress |
| **Alkodami, 2011** | Abstract from Congress |
| **Setina, 2010** | Abstract from Congress |
| **Cresce, 2018** | Abstract from Congress |
| **Wallen, 2018** | Abstract from Congress |
| **Pedro Garcia Bras, 2021** | Abstract from Congress |
| **Arshad, 2021** | Abstract from Congress |
| **Sanchez, 2021** | Abstract from Congress |
| **Park, 2020** | Abstract from Congress |
| **Callegari, 2024** | Abstract from Congress |
| **Giannitsioti E., 2009** | Abstract from Congress |
| **Baraki, 2013** | Abstract from Congress |
| **Taghavi, 2013** | Abstract from Congress |

# **Supplementary Table 2. Additional characteristics of the included studies**

## Supplementary Table 2A. Demographic patient characteristics of the included studies

| **Study, Year** | **Procedure** | **Sample Size** | **Age, yrs** | **Female** | **IVDU** | **Implantable**  **Device** | **LVEF, %** | **Liver**  **Disease** | **Dialysis** | **NYHA**  **III/IV** | **Follow-up,**  **yrs** |
| --- | --- | --- | --- | --- | --- | --- | --- | --- | --- | --- | --- |
| **Baraki,**  **2013[25]** | TVr | 15 (45.5) | 49.0 | 14 (42.4) | 14 (42.4) | 14 (42.4) | NR | NR | 1 (3.0) | 24 (72.7) | 6 |
|  | TVR | 18 (54.5) |  |  |  |  |  |  |  |  |  |
| **Brescia,**  **2022[26]** | TVr | 37 (52.1) | 46.0 | 15 (40.5) | 6 (16.2) | NR | 52 | 3 (8.1) | 5 (13.5) | NR | 3.4 |
|  | TVR | 34 (47.9) | 41.0 | 19 (55.9) | 14 (41.2) |  | 53 | 9 (26.5) | 2 (5.9) |  |  |
| **Dawood,**  **2015[27]** | TVr | 32 (57.1) | 39 | 33 (58.9) | 48 (85.7) | 4* (7.1) | 56 | 35 (62.5) | 2 (3.6) | 10 (17.9) | 4 |
|  | TVR | 24 (42.9) |  |  |  |  |  |  |  |  |  |
| **Di Mauro, 2022[28]** | TVr | 77 (51.7) | 49 | 15 (19.5) | 24 (31.2) | 21 (27.3) | 54 | 2 (2.6) | NR | NR | 19.1 |
|  | TVR | 72 (48.3) | 44 | 21 (29.2) | 34 (47.2) | 15 (20.8) | 55 | 4 (5.6) |  |  |  |
| **Dzilic,**  **2022[29]** | TVr | 16 (50) | 47.8 | 10 (31.3) | 11 (34.4) | 12 (37.5) | NR | 7 (21.9) | 2 (6.3) | 25 (78.1) | 4.6 |
|  | TVR | 16 (50) |  |  |  |  |  |  |  |  |  |
| **Gaca,**  **2013[30]** | TVr | 354 (41.9) | 42 | 161 (45.5) | NR | NR | NR | NR | 53 (15.0) | NR | NR |
|  | TVR | 490 (58.1) | 40.5 | 253 (51.6) |  |  |  |  | 52 (10.6) |  |  |
| **Gottardi,**  **2007[31]** | TVr | 18 (81.8) | 38 | 7 (38.9) | 12 (54.5) | 5 (22.7) | NR | NR | NR | 11 (50.0) | 4.42 |
|  | TVR | 4 (18,2) | 48 | 2 (50.0) |  |  |  |  |  |  |  |
| **Jawad,**  **2020[32]** | TVr | 95 (42.6) | 29.6 | 26 (27.4) | 95 (100) | NR | NR | NR | NR | NR | 2 |
|  | TVR | 128 (57.4) | 31.3 | 25 (19.5) | 128 (100) |  |  |  |  |  |  |
| **Lee,**  **2020[9]** | TVr | 412 (58.5) | 44.8 | 138 (33.5) | 56 (13.6) | NR | NR | 24 (5.8) | 17 (4.1) | NR | 4.43 |
|  | TVR | 292 (41.5) | 47.3 | 99 (33.9) | 87 (29.8) |  |  | 22 (7.5) | 10 (3.4) |  |  |
| **Musci,**  **2007**^†^**[33]** | TVr | 42 (53.2) | 40.7 | 30 (38.0) | 26 (32.9) | NR | NR | NR | NR | NR | 3.59 |
|  | TVR | 31 (39.2) |  |  |  | NR |  |  |  |  |  |
| **Pfannmueller, 2015[34]** | TVr | 34 (60.7) | 53.8 | 17 (30.4) | 11 (19.6) | 15 (26.8) | 60.4 | 8 (14.3) | 8 (14.3) | NR | 4.7 |
|  | TVR | 22 (39.3) |  |  |  |  |  |  |  |  |  |
| **Protos,**  **2018[35]** | TVr | 12 (31.6) | 46 | 8 (66.7) | NR | NR | NR | NR | NR | NR | 1 |
|  | TVR | 26 (68.4) | 31 | 10 (38.5) |  |  |  |  |  |  |  |
| **Renzulli,**  **1999**^†^**[36]** | TVr | 11 (52.4) | 41.9 | 5 (23.8) | 3 (27.3) | 4 (36.4) | NR | NR | NR | 16 (76.2) | 5.72 |
|  | TVR | 10 (47.6) |  |  | 4 (40.0) | 1 (10.0) |  |  |  |  |  |
| **Siddiqui,**  **2022[37]** | TVr | 353 (39.5) | 32.6 | 219 (62.0) | 353 (100) | 3 (0.8) | NR | 66 (18.7) | NR | NR | NR |
|  | TVR | 541 (60.5) | 31.3 | 346 (64.0) | 541 (100) | 4 (0.7) | NR | 105 (19.4) | NR | NR |  |
| **Shetty,**  **2015[38]** | TVr | 5 (71.4) | 33.43 | 3 (42.9) | 7 (100) | NR | NR | NR | NR | NR | 2 |
|  | TVR | 2 (28.6) |  |  |  |  |  |  |  |  |  |
| **Slaughter, 2019[39]** | TVr | 532 (35.6) | 30 | 310 (58.3) | 532 (100) | NR | NR | NR | 32 (6.0) | 85 (16.0) | NR |
|  | TVR | 962 (64.4) | 31 | 593 (61.6) | 962 (100) |  |  |  | 59 (6.1) | 222 (23.1) |  |
| **Thourani,**  **2021[40]** | TVr | 3,654 (75.6) | 34.9 | 2,769 (57.3) | NR | NR | NR | 113 (2.3) | 53 (1.1) | NR | NR |
|  | TVR | 1,177 (24.4) | 34.8 |  |  |  |  |  |  |  |  |
| **Witten,**  **2018**^†^**[10]** | TVr | 93 (69.4) | 49.6 | 57 (42.5) | 40 (29.9) | 35 (26.1) | NR | NR | 18 (13.4) | 46 (34.3) | 5.2 |
|  | TVR | 37 (27.6) |  |  |  |  |  |  |  |  |  |
| **Xie,**  **2023[11]** | TVr | 23 (41.1) | 37.6 | 13 (56.5) | 3 (13.0) | 4* (17.4) | 64.4 | 1 (4.3) | NR | 17 (73.9) | 5.5 |
|  | TVR | 33 (58.9) | 42.2 | 16 (48.5) | 4 (12.1) | 4* (12.1) | 66.3 | 1 (3.0) |  | 23 (69.7) |  |

All binary data are reported as counts and frequencies, n (%).

Continuous data are reported as the mean or median.

All the included studies were retrospective cohort observational studies.

^†^ Whole cohort baseline reported; ^*^ Infected pacemakers.

Abbreviations: IVDU: Intravenous drug user; LVEF: left ventricular ejection fraction; NR: not reported; NYHA: New York Heart Association; TVr: Tricuspid Valve repair; TVR: Tricuspid Valve Replacement; yrs: years

##

## Supplementary Table 2B. Additional demographic patient characteristics of the included studies

| **Study,**  **Year** | **Recruitment**  **Period** | **Procedure** | **Hypertension** | **Prior**  **Cardiac**  **Surgery** | **Urgent or**  **Emergent**  **Surgeries** | **Renal**  **Injury** | **Cross-Clamp**  **time, min** |
| --- | --- | --- | --- | --- | --- | --- | --- |
| **Baraki,**  **2013[25]** | June, 1996 - September, 2012 | TVr | NR | 7 (21.2) | 7 (21.2) | 11 (33.3) | 81 |
|  |  | TVR | NR |  |  |  |  |
| **Brescia,**  **2022[26]** | July, 1992 - December, 2018 | TVr | 14 (37.8) | 2 (5.4) | 21 (56.8) | NR | NR |
|  |  | TVR | 15 (44.1) | 3 (8.8) | 34 (100) |  |  |
| **Dawood, 2015[27]** | January, 2002 - December, 2012 | TVr | NR | 4 (7.1) | NR | NR | NR |
|  |  | TVR |  |  |  |  |  |
| **Di Mauro, 2022[28]** | 1983 - 2018 | TVr | 24 (31.2) | NR | NR | 10 (13.0) | 44 |
|  |  | TVR | 15 (20.8) |  |  | 5 (6.9) | 48 |
| **Dzilic,**  **2022[29]** | February, 2001 - June, 2021 | TVr | NR | NR | 24 (75.0) | NR | 49.5 |
|  |  | TVR |  |  |  |  |  |
| **Gaca, 2013[30]** | 2002 - 2009 | TVr | 148 (41.8) | 18 (5.1) | 226 (63.8) | 80 (22.6) | NR |
|  |  | TVR | 172 (35.1) | 22 (4.5) | 297 (60.6) | 112 (22.9) |  |
| **Gottardi, 2007[31]** | October 1997 - July 2004 | TVr | NR | 3 (13.6) | NR | NR | NR |
|  |  | TVR |  |  |  |  |  |
| **Jawad,**  **2020[32]** | January, 2014 - January, 2016 | TVr | NR | NR | NR | 0 (0.0) | NR |
|  |  | TVR |  |  |  | 2 (1.6) |  |
| **Lee,**  **2020[9]** | January, 2000 - December, 2013 | TVr | 87 (21.1) | 30 (7.3) | 50 (12.1) | NR | NR |
|  |  | TVR | 37 (12.7) | 30 (10.3) | 52 (17.8) |  |  |
| **Musci,**  **2007[33]** | April, 1986 - April, 2006 | TVr | NR | NR | 54 (74.0) | NR | NR |
|  |  | TVR |  |  |  |  |  |
| **Pfannmueller, 2015[34]** | June, 1995 - February, 2012 | TVr | 27 (48.2) | 5 (8.9) | NR | 21 (37.5) | 46.9 |
|  |  | TVR |  |  |  |  |  |
| **Protos,**  **2018[35]** | January, 2012 - December, 2016 | TVr | 5 (41.7) | NR | NR | NR | 50 |
|  |  | TVR | 7 (26.9) |  |  |  | 56 |
| **Renzulli,**  **1999[36]** | January, 1981 - January, 1999 | TVr | NR | NR | NR | NR | NR |
|  |  | TVR |  |  |  |  |  |
| **Siddiqui,**  **2022[37]** | 2002 - 2019 | TVr | 123 (34.8) | 3 (0.8) | NR | 16 (4.5) | NR |
|  |  | TVR | 154 (28.5) | 12 (2.2) | NR | 28 (5.2) | NR |
| **Shetty,**  **2015[38]** | March, 2008 - December, 2011 | TVr | 0 (0.0) | NR | NR | 0 (0.0) | NR |
|  |  | TVR |  |  |  |  |  |
| **Slaughter,**  **2019[39]** | 2011 - 2016 | TVr | 99 (18.6) | NR | 399 (75.0) | NR | NR |
|  |  | TVR | 172 (17.9) |  | 798 (83.0) |  |  |
| **Thourani,**  **2021[40]** | July, 2017 - June, 2023 | TVr | NR | 754 (15.6) | 4473 (92.6) | NR | NR |
|  |  | TVR |  |  |  |  |  |
| **Witten,**  **2018[10]** | January, 2002 - January, 2015 | TVr | 79 (60.8) | 54 (41.5) | 7 (5.4) | NR | NR |
|  |  | TVR |  |  |  |  |  |
| **Xie,**  **2023[11]** | January, 2006 - August, 2019 | TVr | 0 (0.0) | 3 (13.0) | NR | NR | 79.7 |
|  |  | TVR | 2 (6.1) | 3 (9.1) |  |  | 107.4 |

All binary data reported in counts and frequencies, n (%). Continuous data is reported in mean or median. All included studies are retrospective cohort observational studies.

^†^: The whole cohort baseline reported; ^*^:Abbreviations: min: minutes, NR: Not reported, n: number; NYHA: New York Heart Association; TVr: Tricuspid Valve repair; TVR: Tricuspid Valve Replacement

## Supplementary Table 2C. TV Repair and Replacement characteristics of the included studies

| **Study,**  **Year** | **Repair** | | | **Replacement** | |
| --- | --- | --- | --- | --- | --- |
|  | **Leaflet**  **Repair** | **De Vega** | **Ring**  **annuloplasty** | **Bioprosthesis** | **Mechanical** |
| **Baraki,**  **2013[25]** | 6 (40.0) | 6 (40.0) | 3 (20.0) | 14 (77.8) | 4 (22.2) |
|  |  |  |  |  |  |
| **Brescia,**  **2022[26]** | NR | NR | NR | NR | NR |
|  |  |  |  |  |  |
| **Dawood, 2015[27]** | 5 (15.6) | 1 (3.1) | 20 (62.5) | 24 (100) | 0 (0) |
|  |  |  |  |  |  |
| **Di Mauro, 2022[28]** | 25 (32.5) | NR | NR | 46 (63.9) | 13 (18.1) |
|  |  |  |  |  |  |
| **Dzilic,**  **2022[29]** | 12 (75.0) | 1 (6.3) | 13 (81.3) | 13 (81.3) | 3 (18.7) |
|  |  |  |  |  |  |
| **Gaca, 2013[30]** | NR | NR | 121 (34.2) | 450 (91.8) | 32 (6.5) |
|  |  |  |  |  |  |
| **Gottardi, 2007[31]** | NR | NR | 18 (100) | 3 (75.0) | 1 (25.0) |
|  |  |  |  |  |  |
| **Jawad,**  **2020[32]** | NR | NR | 0 (0.0) | NR | NR |
|  |  |  |  |  |  |
| **Lee,**  **2020[9]** | NR | NR | NR | NR | NR |
|  |  |  |  |  |  |
| **Musci,**  **2007[33]** | NR | 14 (33.3) | 3 (7.1) | 17 (54.8) | 3 (12.9) |
|  |  |  |  |  |  |
| **Pfannmueller, 2015[34]** | 21 (61.8) | 8 (23.5) | 5 (14.7) | 17 (77.3) | 5 (22.7) |
|  |  |  |  |  |  |
| **Protos,**  **2018[35]** | NR | NR | NR | 26 (100) | 0 (0) |
|  |  |  |  |  |  |
| **Renzulli, 1999[36]** | 4 (36.4) | 5 (45.5) | NR | 8 (80.0) | 2 (20.0) |
|  |  |  |  |  |  |
| **Siddiqui, 2022[37]** | NR | NR | NR | NR | NR |
| **Shetty,**  **2015[38]** | NR | NR | NR | NR | NR |
|  |  |  |  |  |  |
| **Slaughter,**  **2019[39]** | NR | NR | NR | NR | NR |
|  |  |  |  |  |  |
| **Thourani,**  **2021[40]** | NR | NR | NR | NR | NR |
|  |  |  |  |  |  |
| **Witten,**  **2018[10]** | NR | NR | 27 (29.0) | 27 (100) | 0 (0) |
|  |  |  |  |  |  |
| **Xie,**  **2023[11]** | NR | 23 (100) | 23 (100) | 22 (66.7) | 11 (33.3) |
|  |  |  |  |  |  |

All binary data reported in counts and frequencies, n (%).

Continuous data is reported in mean or median.

All included studies are retrospective cohort observational studies.

^†^: The whole cohort baseline reported; ^*^: Infected pacemakers:

Abbreviations: IVDU: Intravenous drug user; LVEF: Left Ventricle Ejection Fraction; NR: Not reported, n: number;

NYHA: New York Heart Association; TVr: Tricuspid Valve repair; TVR: Tricuspid Valve Replacement; yrs: years

# **Supplementary Table 3. Indication for surgery per included study**

| **Study and Year** | **Indication for surgery** |
| --- | --- |
| **Baraki,**  **2013[25]** | NR |
| **Brescia,**  **2022[26]** | NR |
| **Dawood,**  **2014[27]** | “Indications for operative intervention were based on clinical evaluation and echocardiographic data, as depicted in Figure 2. Patients underwent early operation if there was concomitant left-sided endocarditis with indications for operation , atrial septal defect , infected pacemaker lead , or prosthetic TVIE . The mean TV vegetation size was 1.7 0.6 cm. Among the 20 patients with concomitant left-sided endocarditis, there were 10 patients with mitral valve IE and 10 with aortic valve IE. Mitral pathology included anterior), posterior , or bileaflet vegetations), and prosthetic valve endocarditis). Aortic valve pathology included root abscess and vegetations on the noncoronary, left , and right cusps . The remaining 27 patients with isolated, native TVIE were initially treated with intravenous antibiotics for a mean of 4.4 ± 2.5 weeks. Five patients completed a 6-week course of intravenous antibiotics before undergoing operation. Indications in this group included symptomatic severe TR , persistent vegetation with bacteremia , and persistent fevers with symptomatic severe TR. Twenty-two patients did not complete the course of antibiotic therapy and underwent operation at a mean interval of 36 ± 136.4 days (median,24 days) after diagnosis for persistent severe TR , persistent fevers/bacteremia, patient-specific factors; noncompliance, immunosuppression), and vegetation embolization into a main pulmonary  artery branch .”  *MS; page 3* *Indications for Operation section* |
| **Di Mauro,**  **2022[28]** | NR |
| **Dzilic,**  **2022[29]** | “TV surgery was indicated due to vegetation size greater than 20 mm  in 16 patients (50%), right heart failure due to severe TR in 9 patients  (28.1%), and persisting infection defined as bacteremia over 7 days despite antibiotic treatment in 7 patients (21.9%).”  *MS; Page 3; Indications for surgery section* |
| **Gaca,**  **2013[30]** | NR |
| **Gottardi,**  **2007[31]** | “Indication for surgery was largely based on the echocardiographic findings such as significant tricuspid valve insufficiency (grade III or IV) or vegetation size (larger than 1.5 cm) as well as vegetation morphologic features with additional hemodynamic compromise. If the clinical situation allowed, we tried to postpone surgery until the infection decreased.”  *MS; Page 3; Indication for Surgery section* |
| **Jawad,**  **2020[32]** | “From our limited experience; the main indication for surgery was the failure of medical treatment:  1. Optimum medical therapy is implemented, but the patient still cannot obtain two successive negative blood cultures, denoting a virulent organism (very risky patient; depends on the individual surgeon's decision).  2. Optimum medical therapy is implemented, but the patient still has sizable vegetations that are not regressing in size.  3. Optimum medical treatment is implemented, but the patient still has severe TR even without sizable or detectable masses by TTE.  4. Vegetations or valve destruction with negative culture develop.  5. The patient still has episodes of fever that regress with medical treatment and recur when stopped, regardless of negative cultures.  6. Local valve invasions such as abscess formation or heart block develop (depends on the surgeon's individual decision).  The main contraindications for surgery are relative and from our experience they were:  1.Failure to enroll in certified rehabilitation programs.  2.Failure to pass the final psychiatric evaluation confirming dependence-free condition.  3. Unfavorable metabolic profile, denoting toxemia or septicemia, for instance, liver profile of double average values of serum glutamic pyruvic transaminase (SGPT) or serum glutamic pyruvic transaminase (SGOT), international normalized ratio (INR) >1.7, abnormal gamma-glutamyl transferase (GGT), preoperative hemoglobin <7.5 gm/dl, white blood cell count >17,000 cells/mcl, platelets count <80,000 per microliter (relative contraindication depending on surgeon).  4.Patients with preoperative O2 saturation <90% by pulse oximetry.  5.Persistence of high-grade fever three days before surgery.”  *Supp; Page 1, Timing,indications and contraindications for surgery section* |
| **Lee,**  **2020[9]** | NR |
| **Musci,**  **2007[33]** | “Indications for surgical treatment are better defined for left than for right-sided endocarditis. Uncontrolled sepsis, fever persisting for more than 3 weeks of adequate antibiotic  treatment and intractable right heart failure despite appropriate medical treatment, are the most important indications for surgical intervention. Following the EuropeanSociety for Cardiology (ESC) which 2 years ago published guidelines on the treatment of AIE surgery for RSE is also necessary if tricuspid vegetations are larger than 20 mm after recurrent pulmonary embolism.”  *MS; Page 2; Introduction Section* |
| **Pfannmueller,**  **2015[34]** | NR |
| **Protos,**  **2018[35]** | NR |
| **Renzulli,**  **1999[36]** | “Indications to valve repair were vegetation and signs of infection on a single leaflet or on the posterior leaflet with minimal involvement of anterior leaflet (not more than one third of its surface); multileaflet involvement was considered a contraindication to reparative procedures and was treated by valve replacement.”  *MS; Page 2; Tricuspid valve repair section* |
| **Siddiqui,**  **2022[37]** | NR |
| **Shetty,**  **2015[38]** | NR |
| **Slaughter,**  **2019[39]** | NR |
| **Thourani,**  **2024[40]** | NR |
| **Witten,**  **2018[10]** | “Factors persuasive for surgery (indications) were identified by review of hospital records and operative reports. They included severe valvular regurgitation from valve damage or destruction or prosthetic valve dehiscence; right heart failure with peripheral edema, hepatic congestion, right ventricular systolic dysfunction, and right ventricular dilatation; left heart failure with pulmonary congestion, reduced ejection fraction, left ventricular dilatation, and low cardiac index; septic emboli; large vegetations greater than 15 mm in 1 direction on echocardiogram; failure of medical therapy with sepsis lasting more than 5 to 7 days; fistulas, abscesses, or pseudoaneurysm formation; and a conduction defect such as worsening degree of heart block. In patients with both right- and left-sided IE, the left-sided disease often provided the main indications for surgery.”  *MS; Page 2; Indications for Surgery section* |
| **Xie,**  **2023[11]** | “The indications for surgery were: (1) septicemia that cannot be controlled by drugs; (2) right ventricular insufficiency that cannot be controlled by drugs; (3) formation of perivalvular abscess; (4) right ventricular IE caused by fungi or other refractory pathogens; (5) recurrent pulmonary embolism; (6) combined right- and left-sided IE; (7) persistent fever; and (8) vegetation diameter>20 mm.”  *MS, Page 2; Indications for surgery section* |

*Abbreviations: MS: Main Manuscript; NR: Not reported; and Supp.: Supplementary*

# **Supplementary Table 4. Surgery description per included study**

| **Study and Year** | **Surgical Description** |
| --- | --- |
| **Baraki,**  **2013[25]** | “The standard surgical approach was a median sternotomy and central cannulation (bicaval and ascending aorta). A right anterolateral thoracotomy was performed in 7 cases (21%), exclusively in redo procedures. In these cases cardiopulmonary bypass was established by peripheral cannulation of the femoral artery and the femoral and jugular vein. Depending on surgeon preference and the underlying pathology, the operative procedures were performed on the beating (n=14, 42%) or arrested (n=19, 58%) heart. Cardiac arrest was achieved with antegrade cold blood cardioplegia with a mean cross-clamp time of 46±28min and a mean bypass time of 81±43min. In patients with pacemaker lead infection, complete removal of all foreign material and extensive debridement at the site of the device pocket were done as previously described. In brief, after establishing the cardiopulmonary bypass, the leads were cut in the superior vena cava, and the distal portion was explanted. The device pocket and proximal lead segments were explanted at the end after closure of the sternotomy. Extraction sheaths were used as required to free the lead remnant from adhesions under the clavicle. Temporary pacing was achieved via epicardial temporary pacing wires. Permanent epicardial pacemaker was implanted after 2 weeks of i.v. antibiotic therapy via an anterolateral mini thoracotomy or a subxiphoidal approach. In the case of drug addiction there was no standardized protocol. Valve repair was the major priority in these patients, accepting an insufficiency up to grade II. Of the 14 patients with drug abuse, tricuspid repair was done in 9 patients and replacement with bio- and mechanical prosthesis were done in 2 and 3 patients, respectively. All patients received antibiotics for 6 weeks and anticoagulation with warfarin for at least 3 months postoperatively. Patients with additional indications for long-term anticoagulation such as mechanical prostheses and atrial fibrillation were continued on anticoagulation based on individual decisions.  *MS, Page 2, Surgery Section* |
| **Brescia,**  **2022[26]** | “For tricuspid valve surgery for endocarditis, repair is aggressively pursued over replacement. Tricuspid repair techniques typically included pericardium leaflet patch repair as necessary, bicuspidization, e-PTFE neochords, and prosthetic annuloplasty as described in detail previously. It is our practice to attempt to intervene as early as possible after presentation in an effort to limit tissue destruction and preserve tissue integrity to maximize the likelihood of valve repair. Whenever possible, we scheduled operative intervention in the setting of acute mitral endocarditis, after approximately 72 to 96 hours of presentation and evaluation.”  *MS; Page 2; Operative Techniques and Clinical Management Section* |
| **Dawood,**  **2015[27]** | “All patients underwent the TV operation through a median sternotomy with cardiopulmonary bypass using a bicaval venous drainage technique. Use of a cross-clamp and cardioplegic cardiac arrest was determined by surgeon preference. All efforts were made to repair the valve. Techniques used for valve repair included annuloplasty ring insertion, excision of vegetation and reconstruction using fresh autologous pericardium, leaflet bicuspidization, leaflet sliding-plasty, and expanded polytetrafluoroethylene chordal reconstruction. If a quality repair was not achievable, the valve was replaced with a tissue valve. Early in this experience we used inverted stentless porcine aortic root prostheses in the tricuspid position, but more recently, we have favored stented bioprosthetic valves. When TV replacement was necessary, we routinely placed a permanent epicardial pacing lead.”  *MS; Page 4; Operative Techniques section* |
| **Dimauro,**  **2022[28]** | NR |
| **Dzelic,**  **2022[29]** | “All operations were performed through median sternotomy on cardiopulmonary bypass with bicaval and ascending aortic cannulation. Myocardial protection was achieved using cold (4°C) crystalloid cardioplegia (Bretschneider solution) (n = 24; 75%) or warm blood  cardioplegia (n = 4; 12.5%). Four procedures (12.5%) were performed  on beating heart. In patients with cardiac device infection, the foreign  material was removed.”  *MS; Page 3; Operative Data Section* |
| **Gaca,**  **2013[30]** | “The majority of cases in each subgroup, replacement (79.8%), repair (75.5%), and valvectomy (51.8%), were performed with aortic cross-clamping. In the replacement group, the median implant size was 31 mm, with the median  annuloplasty size of 28 mm in the repair group. Figure 1 demonstrates the yearly rates of repair, replacement, and valvectomy. The type of operation performed is illustrated in Figure 2. In Figure 2A, the majority of patients undergoing valve replacement received a bioprosthetic valve (n 1⁄4 450;  91.8%). Mechanical valves were implanted in 32 patients (6.5%), with the remainder of patients receiving a homograft (n 1⁄4 1), an autograft (n 1⁄4 1), or not recorded (n 1⁄4 6).Figure 2B illustrates the type of valve repair. In this group, 121 patients received an annuloplasty ring only, with the majority (n 1⁄4 212) of patients having had no device implanted.”  *MS; Page 3; Results Section* |
| **Gottardi,**  **2007[31]** | “Several reconstructive techniques were used depending on the site and extent of endocarditic lesions. In most of the patients with infected pacemaker leads (n 4), the endocarditic lesion was limited to the posterior leaflet. In  those patients, bicuspid valve formation of the tricuspid valve was performed as follows: the posterior leaflet was completely excised, the anterior and septal leaflets were partially mobilized, plication sutures were put in place,  and a sliding plasty of the remaining two leaflets was performed (Fig.1). In case of leaflet perforation or endocarditic vegetations within the leaflet, the respective lesion was excised and a patch plasty was performed, using an autologous pericardial patch. This technique was used in case of defects or vegetations involving either the anterior leaflet (n = 2), the septal leaflet (n = 3), or even both leaflets(n = 2; Fig 2).In cases of limited leaflet destruction but involvement of the subvalvular apparatus (n 3), the infected or destroyed leaflet tissue including the involved chordae was completely resected. The defect was closed by performing a quadrangular excision of healthy leaflet tissue of the opposing leaflet including the attached chordae and transposition to the primary defect. Depending on the size of the excised healthy leaflet tissue, the resulting defect was closed by readapting the leaflet with or without a concomitant sliding plasty (Fig 3). In 2 patients the endocarditic lesion was limited to one commissure. In those patients, the infected tissue was excised and a sliding plasty of the adjacent leaflets was performed similar to the technique described for bicuspid valve formation.In 2 patients, more than one of the described techniques were combined. In all patients, a prosthetic annuloplasty ring was implanted to stabilize the valve geometry and to prevent future ring dilatation. In this series the original Carpentier-Edwards tricuspid annuloplasty ring and, more recently, the Edwards Lifescience MC3 annuloplasty system were used. The predominant  size of rings implanted was 30 mm.”  *MS; Page 3; Reconstructive Techniques section* |
| **Jawad,**  **2020[32]** | **“Valve Assessment:** After the establishment of normothermic cardiopulmonary bypass, all procedures were done on the beating heart by right atriotomy. Proper visual examination of the valve was done. If the septal leaflet was affected, the decision was a replacement from the start because the repair technique requires extensive suturing at the annulus itself. That makes the annulus relatively edematous, thus increasing the incidence of heart block in this area, which represents the base of the triangle of Koch. If the damage was limited to 1 leaflet, then that was a more encouraging factor for repair than if 2 leaflets were affected (Figure 1).  **Tricuspid Valve Repair:** An autologous pericardial patch was harvested and  treated with 0.5% glutaraldehyde solution for 10 to 15 minutes. The patch was then fashioned into a half-circle shape with the base toward the annulus. A 5-0 poly-propylene suture was used to attach the patch base to the annulus with continuous running sutures spanning commissure to commissure (Figure 2). Another 5-0 stitch was used to close the commissure between the native spared leaflet and the patch (1-2 mm), and this was used  as an anchor for the patch. This was done from both sides of the patch (Figure 3). The free margin of the patch was trimmed and fashioned with some redundancy to ensure proper coaptation height. The free margin was then fixed with 4-0 or 5-0 Gore-Tex (W. L. Gore & Associates, Flagstaff, AZ) neochord sutures to the base of each corresponding papillary muscle with allowance for billowing of the new leaflet (Figure 4). No annuloplasty ring was used to minimize artificial foreign substances in a potentially infected field. The water test was then applied, and the final length of neochord was fixed (Figure 5). Right atrial closure was done by inverted Lembert sutures and overrunning continuous sutures by 4-0 polypropylene suture (Video 1). Caval de-snaring was done, and the result was evaluated by transesophageal echocardiography for possible correction.Reconstruction of both anterior and posterior leaflets in cardioplegic arrested heart is shown in Video 2.  **Tricuspid Valve Replacement:** Interrupted 2-0 Ti-Cron Braided Polyester Sutures with PTFE Pledget (Covidien, New Haven, CT) with Teflon pledgets (Chemours, Wilmington, DE) was applied to the annulus directly, except at the septal leaflet attachment, where the stitches were applied to the hinge of the partially preserved septal leaflet to avoid possible heart block. Operating on a beating heart allows careful individual suture placement, reflected directly on electrocardiogram. This does not require venting or removing air  from the ascending aorta.”  *MS; Page 3 to 4; Valve Assessment, Tricuspid Valve Repair and Tricuspid Valve Replacement section* |
| **Lee,**  **2020[9]** | NR |
| **Musci,**  **2007[33]** | “Our surgical strategy for active infective endocarditis is based on three principles:  (1) Intensive debridement of the infected area or if the infected process is localized on the valve, vegetectomy followed by intensive irrigation with polyvidon—iodine solution of the infected area. Excision of a vegetation alone is limited to patients with well-circumscribed  vegetation in an otherwise normal valve.  (2) Whenever possible valve repair with homologous or autologous pericardium is attempted avoiding artificial material. Monofilament sutures reinforced with horse pericardium and preserved in polyvidon—iodine solution are used. Fig. 3a—c shows the operative techniques for tricuspid valve reconstruction: after vegetectomy or excision of the perforated leaflet (Fig. 3a) the defect is closed directly or with a pericardial patch (Fig. 3b). To ensure leaflet coaptation, annuloplasty with pericardium is performed (Fig. 3c).  (3) If valve replacement is unavoidable because of extensive endocarditic destruction of the valve a biological substitute without any artificial material on the surface that might become infected is used, the best option being in our opinion either the homograft, which we used  in aortic or pulmonary position or the Shelhigh bioprosthesis, which is our bioprosthesis of choice for endocarditis over the last 6 years.”  *MS; Page 4; Surgical strategy for active infective endocarditis section* |
| **Pfannmueller,**  **2015[34]** | “Surgery was performed either through a median longitudinal sternotomy or a right anterolateral minithoracotomy, the latter having been previously described in detail by our  group. The technique and the decision for or against TV repair/replacement was at the discretion of the surgeon, but predominantly depended on the extent of destruction of the TV.”  *MS; Page 2; Patients and methods section* |
| **Protos,**  **2018[35]** | NR |
| **Renzulli,**  **1999[36]**  **Renzulli,**  **1999**  ***(continued)*** | “The operative approach was through a standard median sternotomy incision. Bicaval cannulas with caval tapes were used for venous drainage in all cases.”  **Tricuspid Repair:**  “Nine patients underwent valve repair under aortic cross-clamp with hypothermic arrest using cold crystalloid cardioplegia infused in the aortic root in 7 cases and directly in the coronary ostia in the other 2. In 2 patients, concomitant aortic valve replacement with bioprostheses was performed. Both of them had aortic vegetations and tear of the noncoronary aortic cusp.”  “Conservative management included wide-margin resection of the vegetation area and no prosthetic tissue, either autologous or artificial, was employed at any time. Whenever the vegetations involved the posterior portion of the tricuspid valve, complete leaflet excision  and Kay annuloplasty was performed. If the vegetations were localized on the anterior leaflet, wide quadrangular resection including the vegetation was performed. In 2 patients, more than three quarters of the anterior leaflet were resected and the leaflet margins were  approximated with a 5/0 prolene suture.”  “A De Vega annuloplasty was performed in all cases of anterior leaflet resection.Intraoperative testing of tricuspid valve continence was performed by injecting cold saline solution into the right ventricle.”  **Tricupisd Replacement:**  “Tricuspid valve replacement was performed in 10 patients, by complete excision of the valvular apparatus apart a rim of lcm of the septal leaflet, and a prosthetic valve was implanted with unpledgeted mattress sutures. In the conduction system area, stitches were driven only through the remnant of the septal leaflet. No prosthesis of less than 31.mm was implanted. The type and size of the implanted prostheses are listed in Table 2. We chose to implant a bioprosthesis in most cases, for fear of thromboembolic complications after tricuspid valve replacement with mechanical prostheses.Resected valvular tissue was sent either for histology or for culture.”  MS; Page 2-3; Material and methods, Tricuspid valve repair and Tricuspid valve replacement section |
| **Siddiqui,**  **2022[37]** | NR |
| **Shetty,**  **2015[38]** | NR |
| **Slaughter,**  **2019[39]** | NR |
| **Thourani,**  **2019[40]** | NR |
| **Witten,**  **2018[10]** | “Once the patient has an indication for operation, surgery is expedited. Our surgical approach is debridement of all infected tissues and foreign material, followed by generous irrigation. Local antiseptics and antibiotics are used sparingly. Tricuspid valve repair or reconstruction is performed whenever possible using a variety of reconstruction techniques, including use of autologous pericardium and artificial chords for leaflet reconstruction (Video 1) with or without suture or ring anuloplasty. Rather than replacing the valve with a prosthetic device, important residual TR was frequently accepted. When replacement is deemed unavoidable, we use a bioprosthesis. Valvectomy without replacement was used once in this series.”  *MS; Page 2; Infective Endocarditis Management and Surgery section* |
| **Xie,**  **2023[11]** | **“Surgical procedure:** All operations were performed under general anesthesia. The sternum was cut in the middle, and a cardiopulmonary bypass was established through the ascending aorta and the superior and inferior vena cava. After cardiopulmonary bypass, the temperature was uniformly cooled to 32°C, and HTK solution was infused from the root of the aorta to protect the myocardium. The size of the tricuspid annulus, the lesion of the tricuspid valve, the location and size of vegetation, the degree of valve lesion, the destruction of the subvalvular structure, and the formation of perivalvular abscess were examined routinely before operation. Based on the exploration results, different tricuspid valve operations were selected. Vegetation was removed in all cases. In patients with left heart valve disease or other intracardiac malformations that required surgical treatment, the left heart valve surgery or intracardiac malformation correction was performed first, followed by the tricuspid valve surgery. After tricuspid valve treatment, opening and closing of the tricuspid valve, conduction block, and other arrhythmias were monitored. Residual tricuspid regurgitation was observed by drawing water. Post-surgery, the effects of the operation on tricuspid valve activity and residual regurgitation were re-evaluated using transesophageal  ultrasonography.”  **“Tricuspid valvuloplasty:** TVP was performed in patients with moderate or severe tricuspid regurgitation; no abnormalities, including calcification, thickening, curl, and shortening, on the tricuspid valve leaflets and subvalvular structures; and a diastolic tricuspid annulus diameter of >40 mm. TVP includes Kay suture valvuloplasty, De Vega annuloplasty, pericar-  dial patch valvuloplasty, and prosthetic ring annuloplasty.”  **“Tricuspid valve replacement:** TVR was performed when the effect of TVP was unsatis-  factory or when severe tricuspid valve insufficiency prevented valvuloplasty during the operation”  *MS; Page 2; Surgical procedure; Tricuspid valvuloplasty; Tricuspid valve replacement section* |

*Abbreviations: MS: Main Manuscript and Supp.: Supplementary*

#

# **Supplementary Table 5. Causative microorganisms description per included study**

| **Study and Year** | **Pathogen** | **Total Cohort** | **Intervention** | **Control** |
| --- | --- | --- | --- | --- |
|  |  | **n(%)** | **n(%)** | **n(%)** |
| **Brescia, 2022[26]** | *Staphylococcus* | 45 (63.4) | 28 (75.7) | 17 (50.0) |
|  | *MSSA* | 34 (47.9) | 17 (45.9) | 17 (50.0) |
|  | *MRSA* | 6 (8.5) | 6 (16.2) | 0 (0.0) |
|  | *Coagulase-negative staphylococcus* | 5 (7.0) | 5 (13.5) | 0 (0.0) |
|  | *Streptococcus* | 7 (9.9) | 1 (2.7) | 6 (17.6) |
|  | *Fungus* | 6 (8.5) | 1 (2.7) | 5 (14.7) |
|  | *Other bacterial* | 9 (12.7) | 4 (10.8) | 5 (14.7) |
| **Dawood, 2015[27]** | *Staphylococcus spp* | 38 (67.9) | NR | NR |
|  | MRSA | 23 (41.1) | NR | NR |
|  | MSSA | 12 (21.4) | NR | NR |
|  | *Streptococcus spp* | 6 (10.7) | NR | NR |
|  | *Enterococcus spp* | 4 (7.1) | NR | NR |
|  | *Pseudomonas spp* | 3 (5.4) | NR | NR |
|  | *Candida spp* | 1 (1.8) | NR | NR |
| **Di Mauro, 2022[28]** | *Staphylococcus aureus* | 74 (49.7) | 36 (46.8) | 38 (52.8) |
|  | *Streptococci* | 21 (14.1) | 9 (11.7) | 12 (16.7) |
|  | *Staphylococcus* other than *S. aureus* | 13 (8.7) | 10 (13.0) | 3 (4.2) |
|  | *Enterococcus* | 2 (1.3) | 1 (1.3) | 1 (1.4) |
|  | *Mycotic* | 2 (1.3) | 2 (2.6) | 0 (0.0) |
|  | *Pseudomonas* | 1 (0.7) | 0 (0.0) | 1 (1.4) |
| **Dzlic, 2022[29]** | *Staphylococcus aureus* | 25 (78.1) | NR | NR |
|  | *MRSA* | 2 (6.3) | NR | NR |
|  | *Staph. epidermidis* | 3 (9.4) | NR | NR |
| **Gottardi, 2007[30]** | *Staphylococcus aureus* | 10 (45.5) | NR | NR |
|  | *Staphylococcus epidermidis* | 2 (9.1) | NR | NR |
|  | *Enterococcus faecalis* | 1 (4.5) | NR | NA |
|  | *Streptococcus milleri* | 1 (4.5) | NR | NR |
| **Jawad, 2020[32]** | *Coagulase-positive Staphylococcus aureus* | 131 (58.7) | NR | NR |
|  | *Coagulase-negative Staphylococci* | 36 (16.1) | NR | NR |
|  | *Coagulase-negative Staphylococcus hominis* | 4 (1.8) | NR | NR |
|  | *Pseudomonas aeruginosa* | 26 (11.7) | NR | NR |
| **Musci, 2007[33]** | *Staphylococcus aureus* | 40 (54.8) | NR | NR |
|  | *Coagulase-negative staphylococcus* | 3 (4.1) | NR | NR |
|  | *Streptococci General* | 9 (12.3) | NR | NR |
|  | *Streptococci Viridans* | 3 (4.1) | NR | NR |
|  | *Streptococci B - Hamoly* | 1 (1.4) | NR | NR |
|  | Enterococcus | 7 (9.6) | NR | NR |
| **Pfannmueller, 2015[34]** | *Staphylococcus aureus* | 24 (42.9) | NR | NR |
|  | *Coagulase-negative staphylococcus* | 10 (17.9) | NR | NR |
|  | *Enterococcus faecalis* | 7 (12.5) | NR | NR |
|  | *Streptococcus spp.* | 7 (12.5) | NR | NR |
| **Protos, 2018[35]** | MRSA | 18 (47.4) | 5 (41.7) | 13 (50.0) |
|  | MSSA | 11 (28.9) | 1 (8.3) | 10 (38.5) |
| **Renzulli, 1999[36]** | *Staphylococcus epidermidis* | 6 (28.6) | NR | NR |
|  | *Staphylococcus aureus* | 3 (14.3) | NR | NR |
|  | *Enterococcus* | 2 (9.5) | NR | NR |
| **Shetty, 2015[38]** | *Staphylococcus aureus* | 6 (85.7) | NR | NR |
|  | *Streptococcus viridans* | 0 (0.0) | NR | NR |
|  | *Enterococcus* | 1 (14.3) | NR | NR |
|  | *HACEK* | 1 (14.3) | NR | NR |
|  | *Fungal* | 3 (42.9) | NR | NR |
| **Slaughter, 2019[39]** | *Staphylococcus aureus* | 1135 (76.0) | 395 (74.2) | 740 (76.9) |
| **Witten, 2018[10]** | *Staphylococcus aureus* | 54 (41.5) | NR | NR |
|  | *Coagulase-negative staphylococcus* | 33 (25.4) | NR | NR |
|  | *Enterococcus* | 8 (6.2) | NR | NR |
|  | *Viridans group streptococcus* | 7 (5.4) | NR | NR |
|  | *Fungus* | 4 (3.1) | NR | NR |
| **Xie, 2023[11]** | *Staphylococcus aureus* | 8 (14.3) | 3 (13.0) | 5 (15.2) |
|  | *Staphylococcus epidermidis* | 3 (5.4) | 2 (8.7) | 1 (3.0) |
|  | *Streptococcus bovis* | 19 (33.9) | 9 (39.1) | 10 (30.3) |
|  | *Acinetobacter baumannii* | 3 (5.4) | 2 (8.7) | 1 (3.0) |
|  | *Enterococcus faecalis* | 3 (5.4) | 0 (0.0) | 3 (9.1) |

#

# **Supplementary Table 6. Grading of Recommendations, Assessment, Development and Evaluation (GRADE)**

| **Endpoint** | **Effect Size** | **Risk of Bias** | **Inconsistency** | **Indirectiness** | **Imprecision** | **Publication Bias** | **Overall Quality of Evidence** |
| --- | --- | --- | --- | --- | --- | --- | --- |
| **Long term**  **all-cause mortality** | HR: 0.77  (95%CI: 0.60 to 0.98; P=0.04) | Serious | Not serious | Serious | Not serious | Undetected | Very Low |
| **Early mortality** | OR: 0.84  (95%CI: 0.63 to 1.12; P=0.22) | Serious | Not serious | Serious | Not serious | Undetected | Very Low |
| **Postoperative stroke** | OR: 1.17  (95%CI: 0.83 to 1.65; P=0.41) | Serious | Not serious | Serious | Not serious | Undetected | Very Low |
| **Postoperative AKI** | OR: 0.79  (95%CI: 0.64 to 0.98; P=0.03) | Serious | Not serious | Serious | Not serious | Undetected | Very Low |
| **Postoperative deep**  **wound infection** | OR: 0.26;  (95%CI: 0.14 to 0.49; P<0.01) | Serious | Not serious | Serious | Not serious | Undetected | Very Low |
| **Postoperative permanent pacemaker implantation** | OR: 0.15  (95% CI: 0.06 to 0.38; P<0.01) | Serious | Serious | Serious | Not Serious | Undetected | Very Low |

*Abbreviations: CI: Confidence interval; HR: Hazard Ratio; OR:Odds ratio*

# **Supplementary Results 1. Reported and reconstructed Kaplan-Meier curves comparison at the study level**

**Brescia, 2022**

**
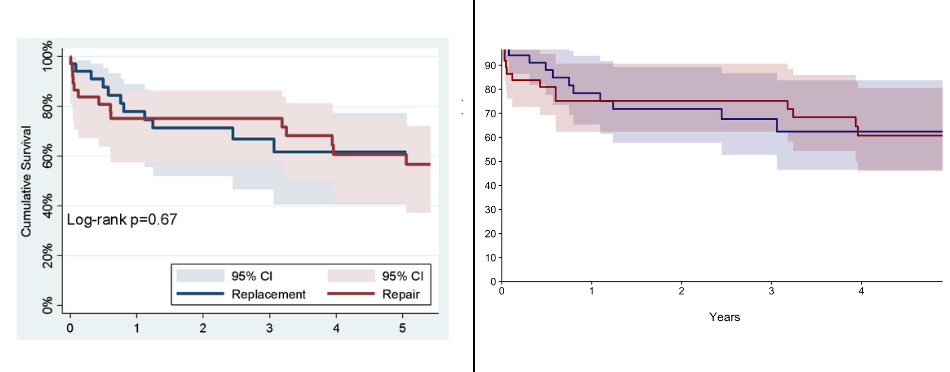
**

**Dawood, 2015**

**
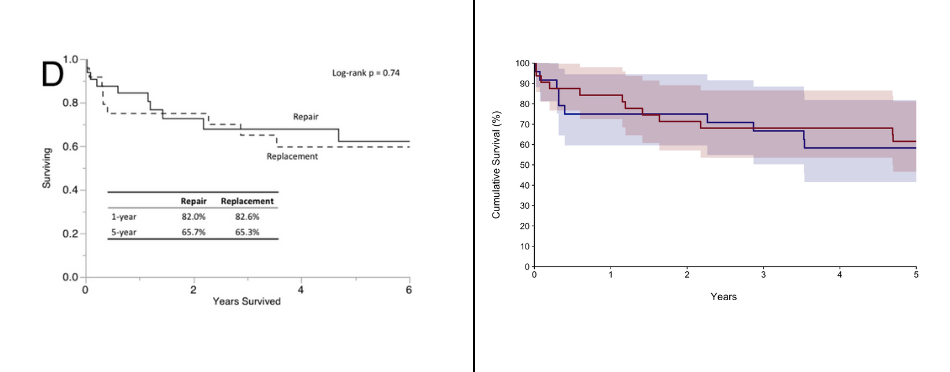
**

**Di Mauro, 2022**

**
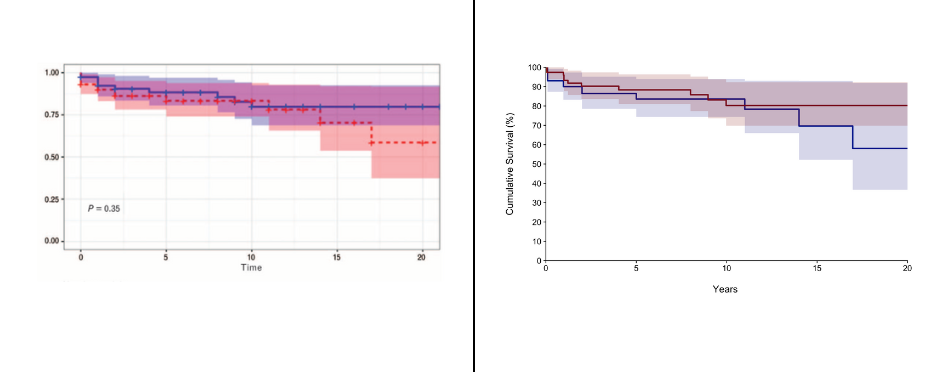
**

**Jawad, 2020**

**
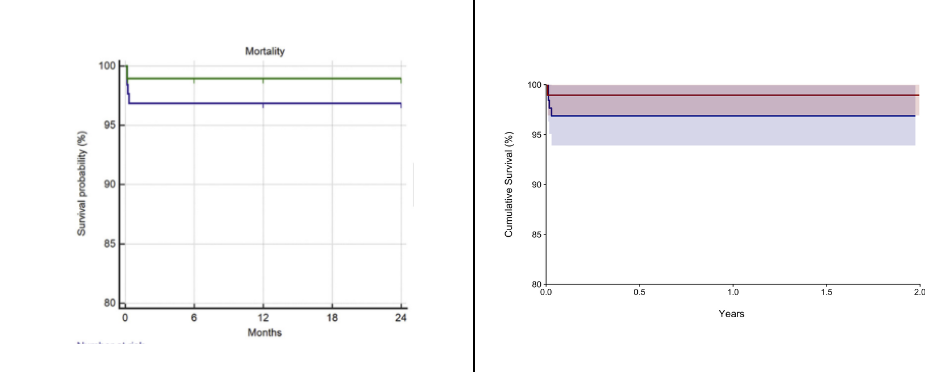
**

**Lee, 2020**

**
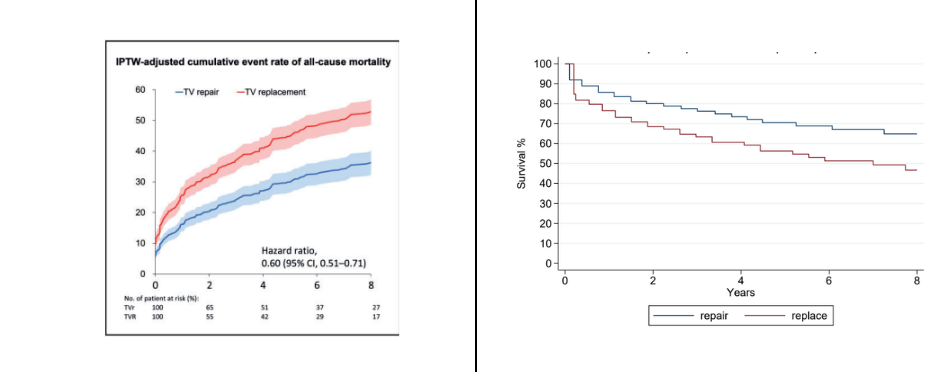
**

**Musci, 2007**

**
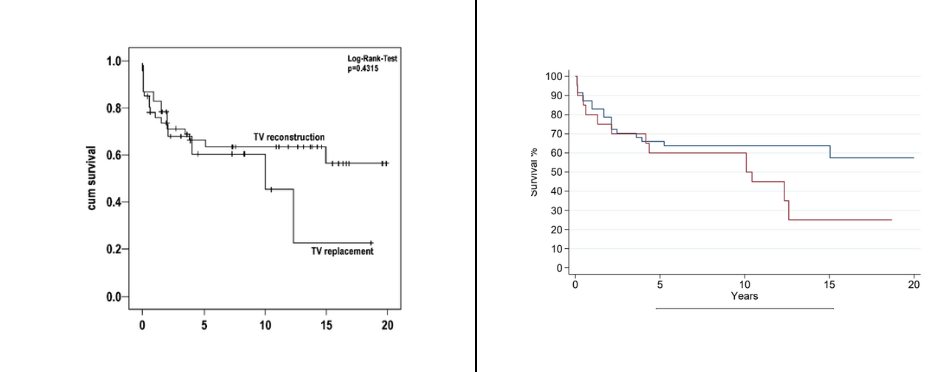
**

**Witten, 2018**

**
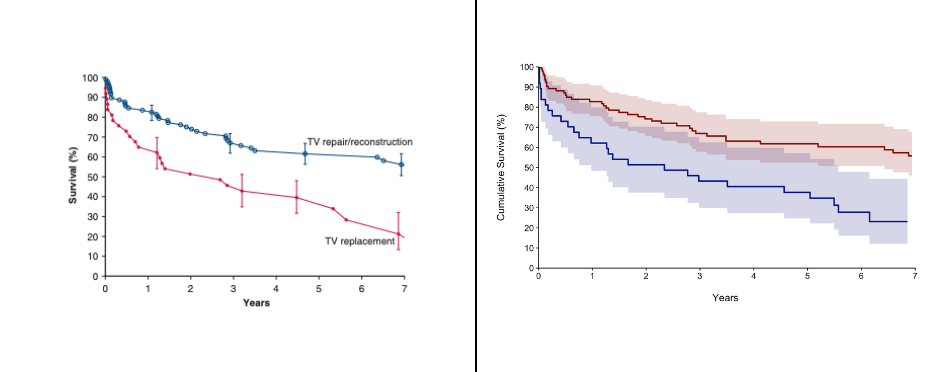
**

**Xie, 2023**

**
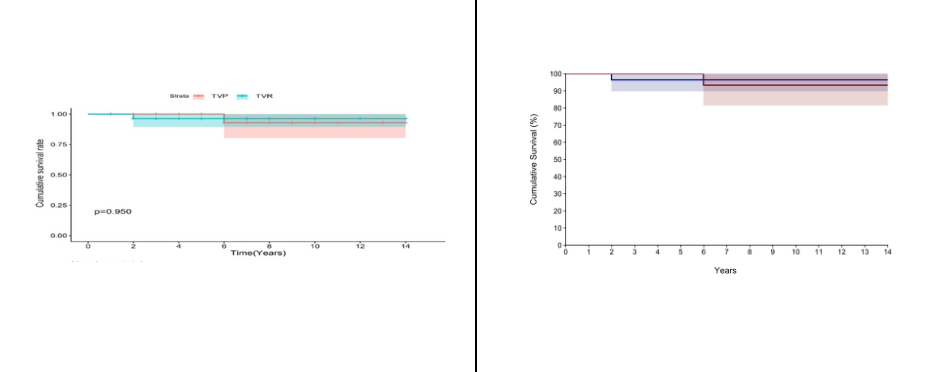
**

#

# **Supplementary Figure 1. PRISMA Flowchart**


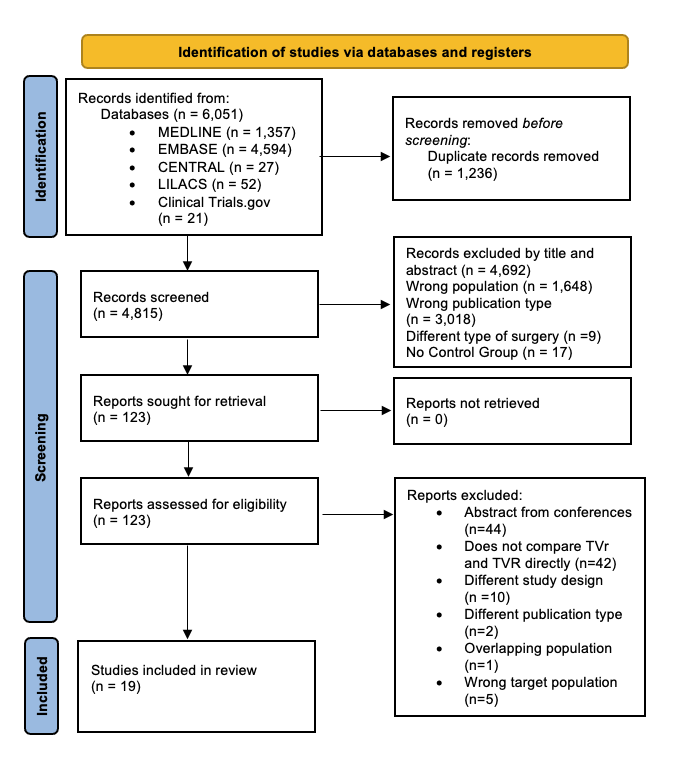


*Legend:* PRISMA 2020 flow diagram for systematic reviews

# **Supplementary Figure 2. Test of Proportional Hazards Assumption**

# **
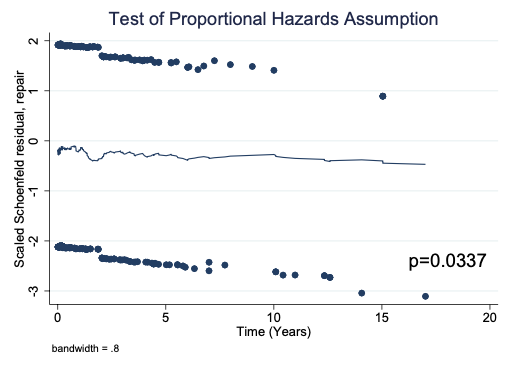
**

# **
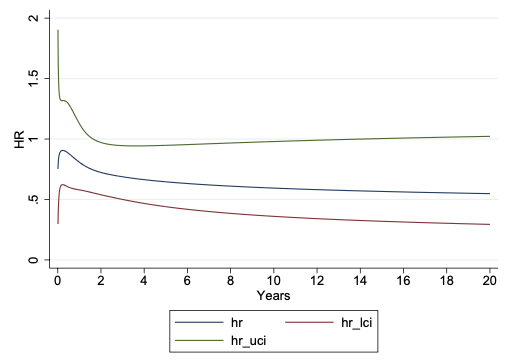
**

# **Supplementary Figure 3. Subanalysis for isolated tricuspid valve cohort**

**
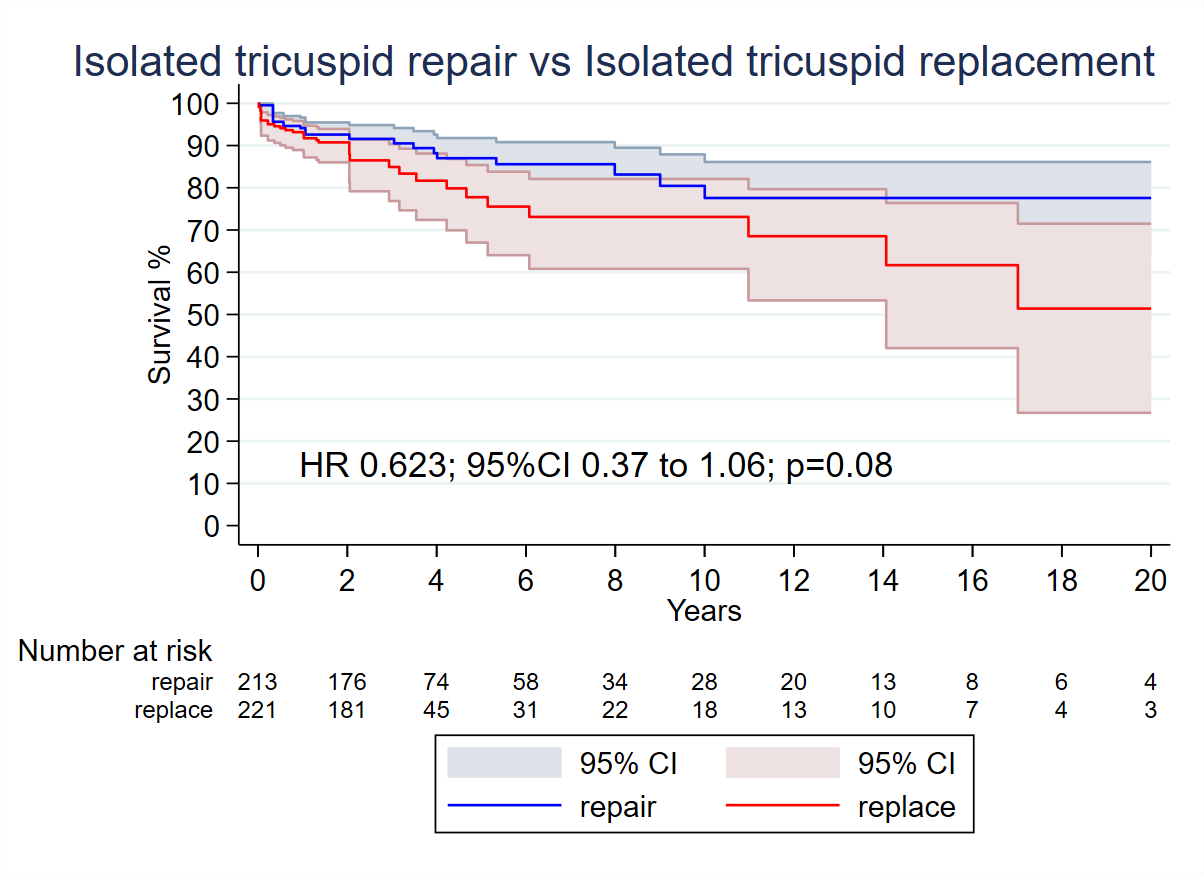
**

Legend: In the isolated TVIE subanalysis, overall survival was not different between the TVr and TVR groups. *Abbreviations* CI: Confidence Interval; HR: Hazard Ratio; TVIE: Tricuspid valve infectious endocarditis; TVr: Tricuspid valve repair; TVR: Tricuspid valve replacement

# **Supplementary Figure 4. Forest plot for secondary endpoints**

## Supplementary Figure 4A. Forest plot for postoperative deep wound infection


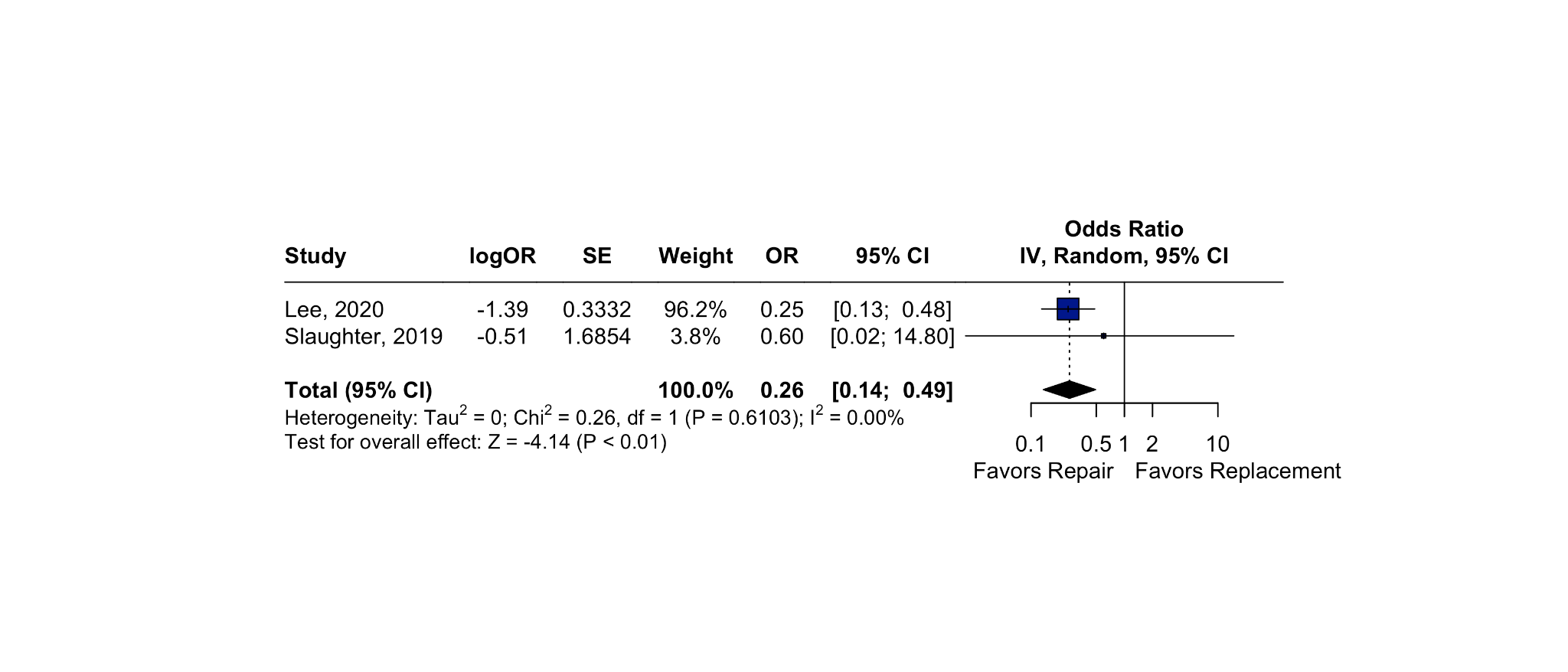


*Legends*: TVr was associated with significantly higher odds of postoperative deep wound infection than TVR. *Abbreviation*: CI: confidence interval; IV: inverse of variance; OR: odds ratio SE: standard error; TVr: Tricuspid valve repair; TVR: Tricuspid valve replacement

## Supplementary Figure 4B. Forest plot for permanent pacemaker implantation


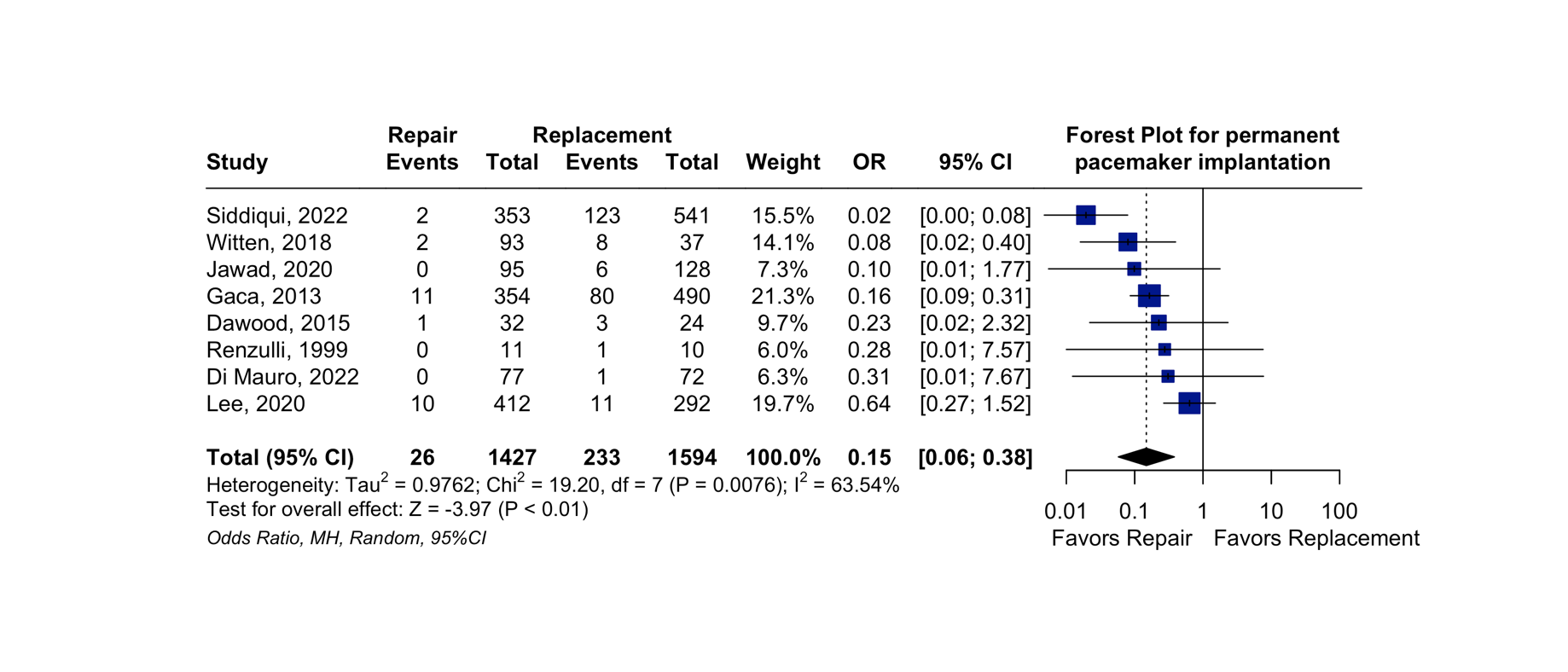


*Legends*: TVr was associated with significantly higher odds of permanent pacemaker implantation than TVR. *Abbreviation*: CI: confidence interval; IV: inverse of variance; OR: odds ratio SE: standard error; TVr: Tricuspid valve repair; TVR: Tricuspid valve replacement

# **Supplementary Figure 5. Sensitivity Analysis**

## Supplementary Figure 5A. Leave-one-out for long-term all-cause mortality endpoint


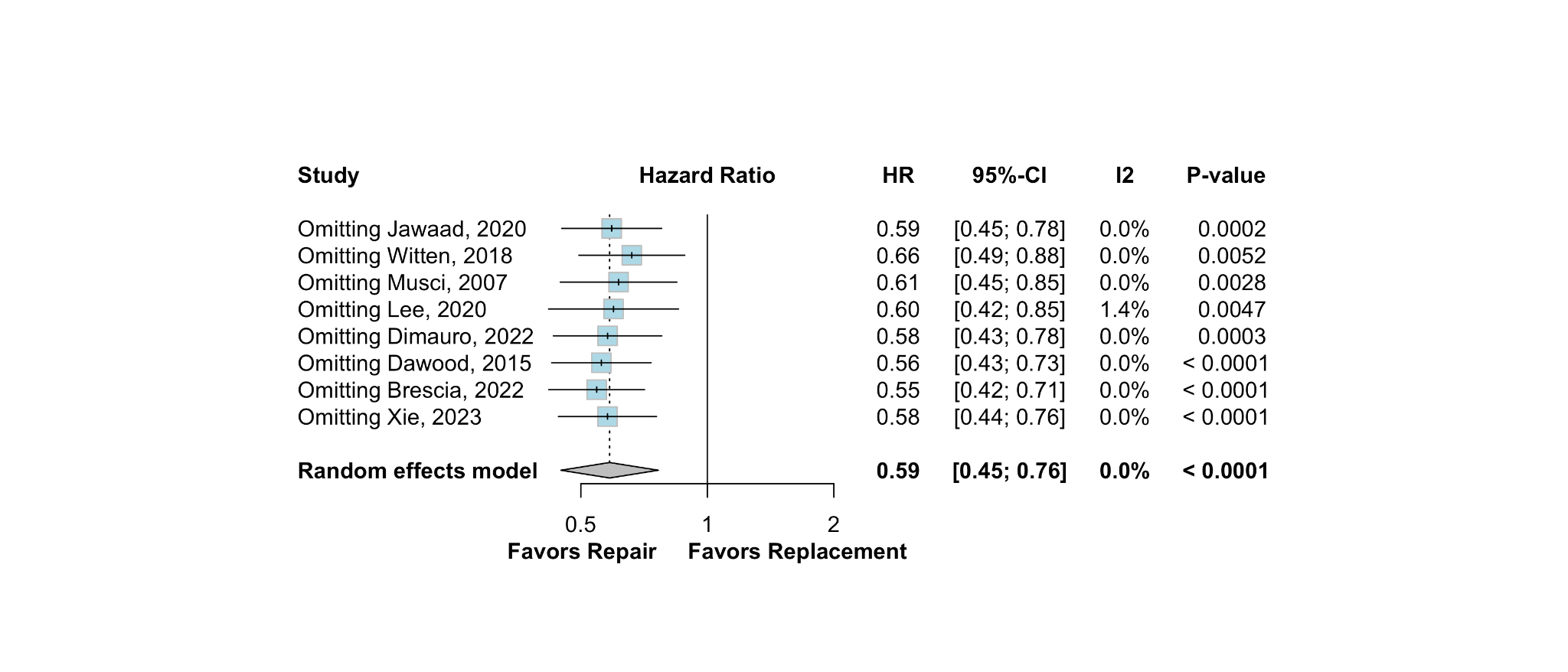


## Supplementary Figure 5B. Leave-one-out for any reoperation endpoint


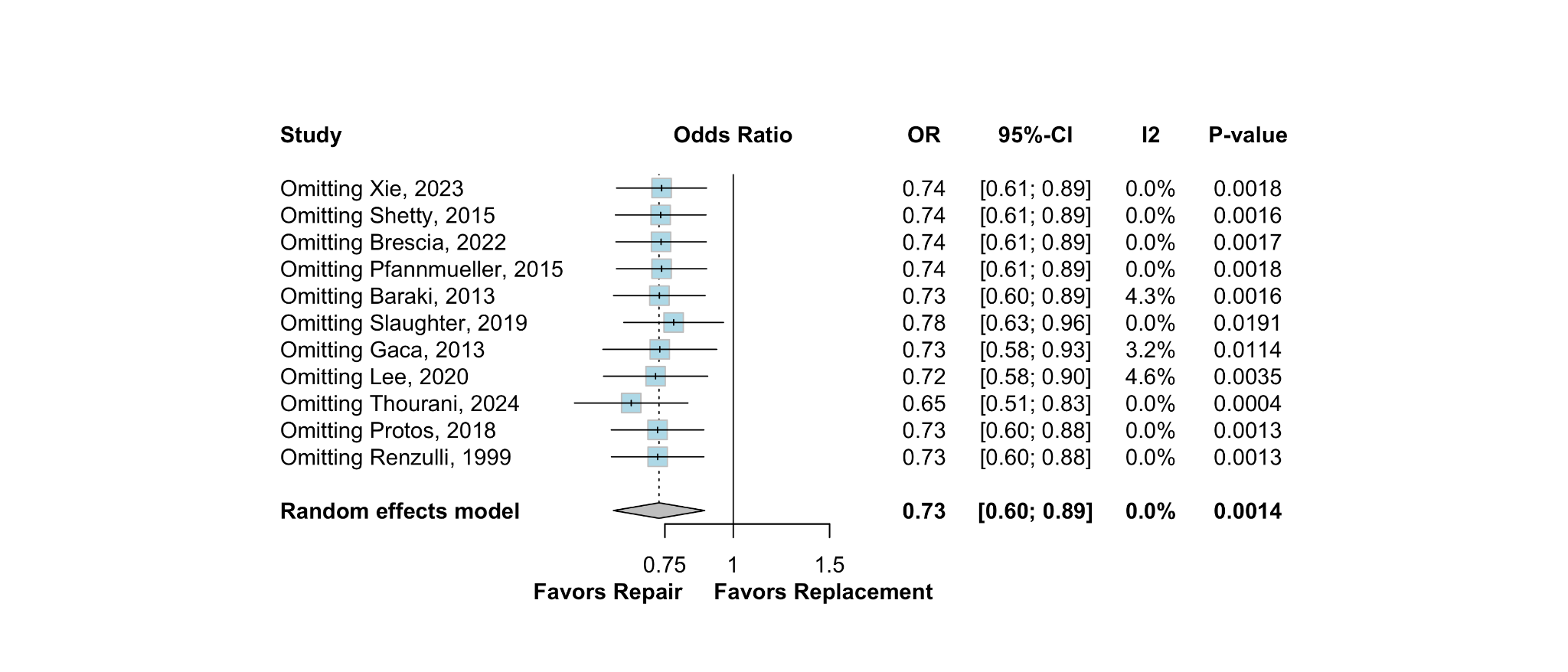


## Supplementary Figure 5C. Leave-one-out for reinfection endpoint


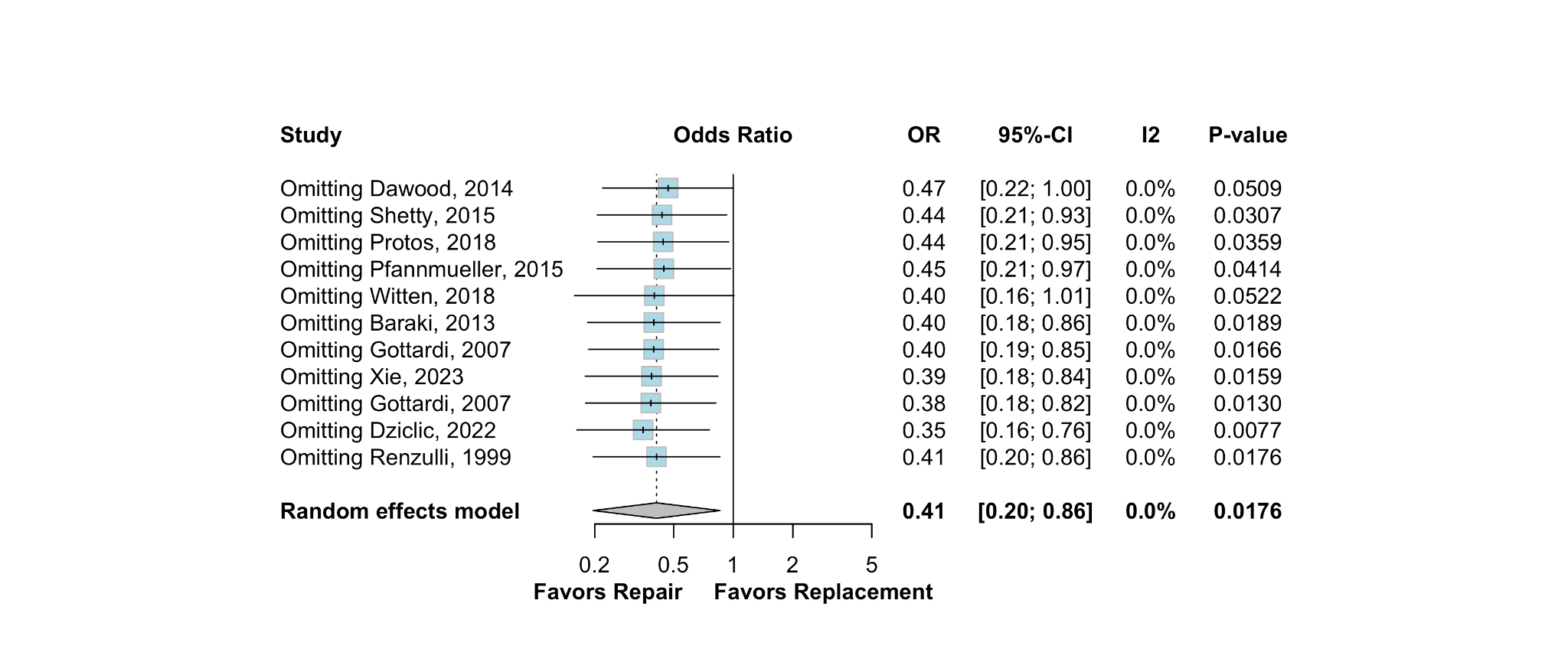


## Supplementary Figure 5D. Leave-one-out for early mortality endpoint


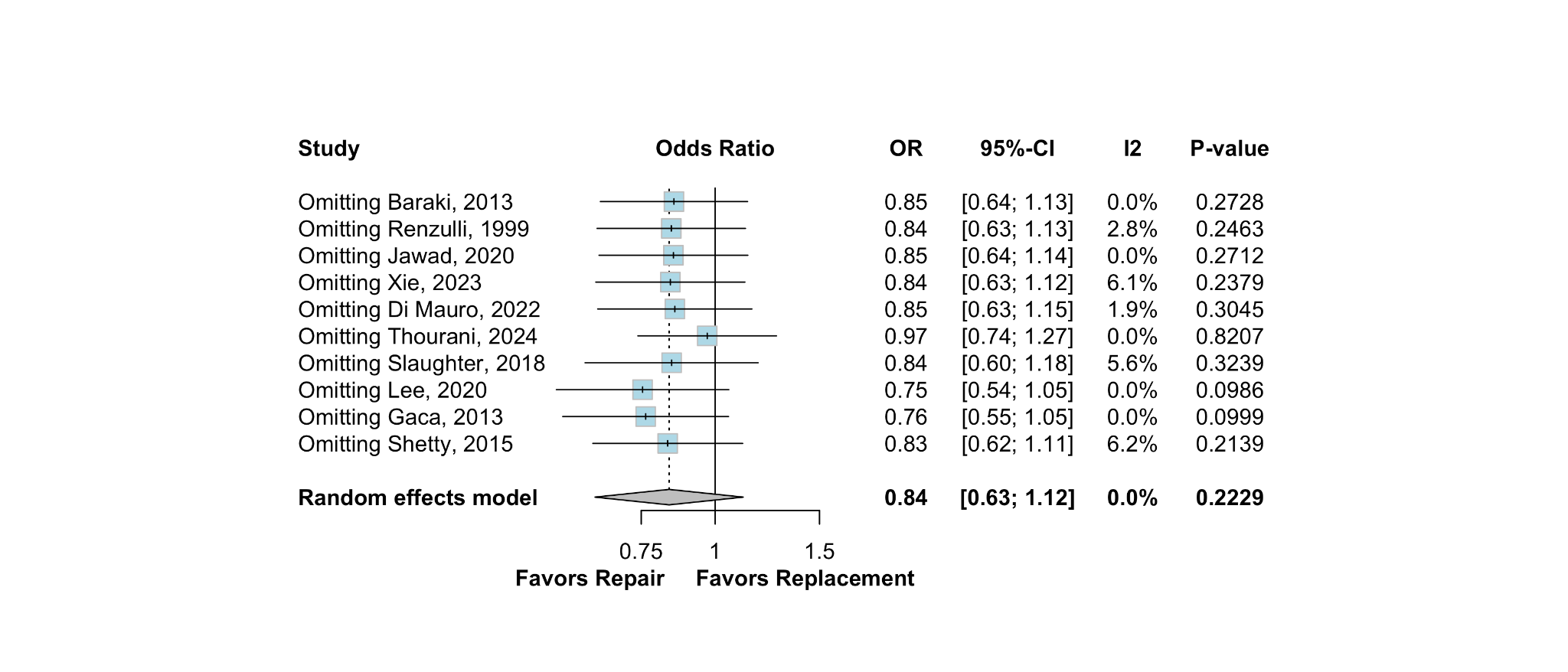


##

## Supplementary Figure 5E. Leave-one-out for postoperative stroke


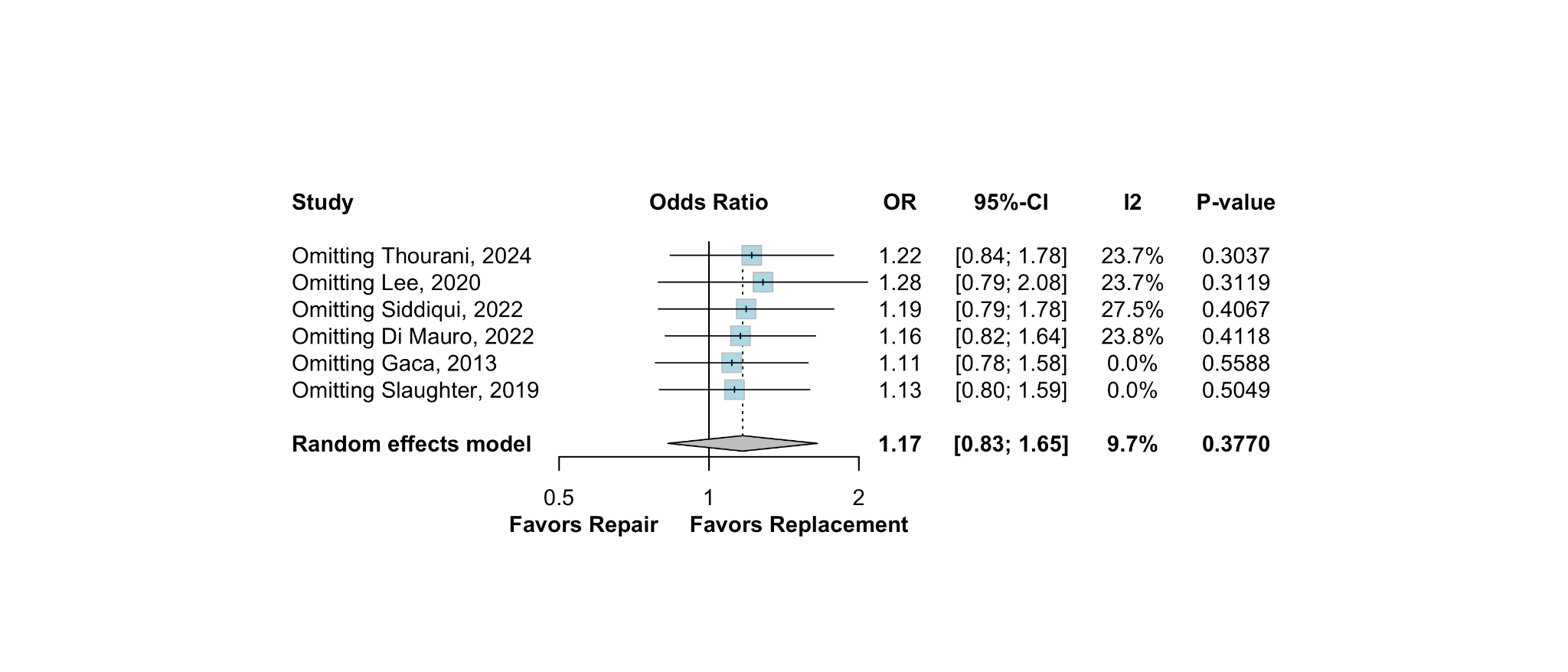


## Supplementary Figure 5F. Leave-one-out for acute kidney injury

##
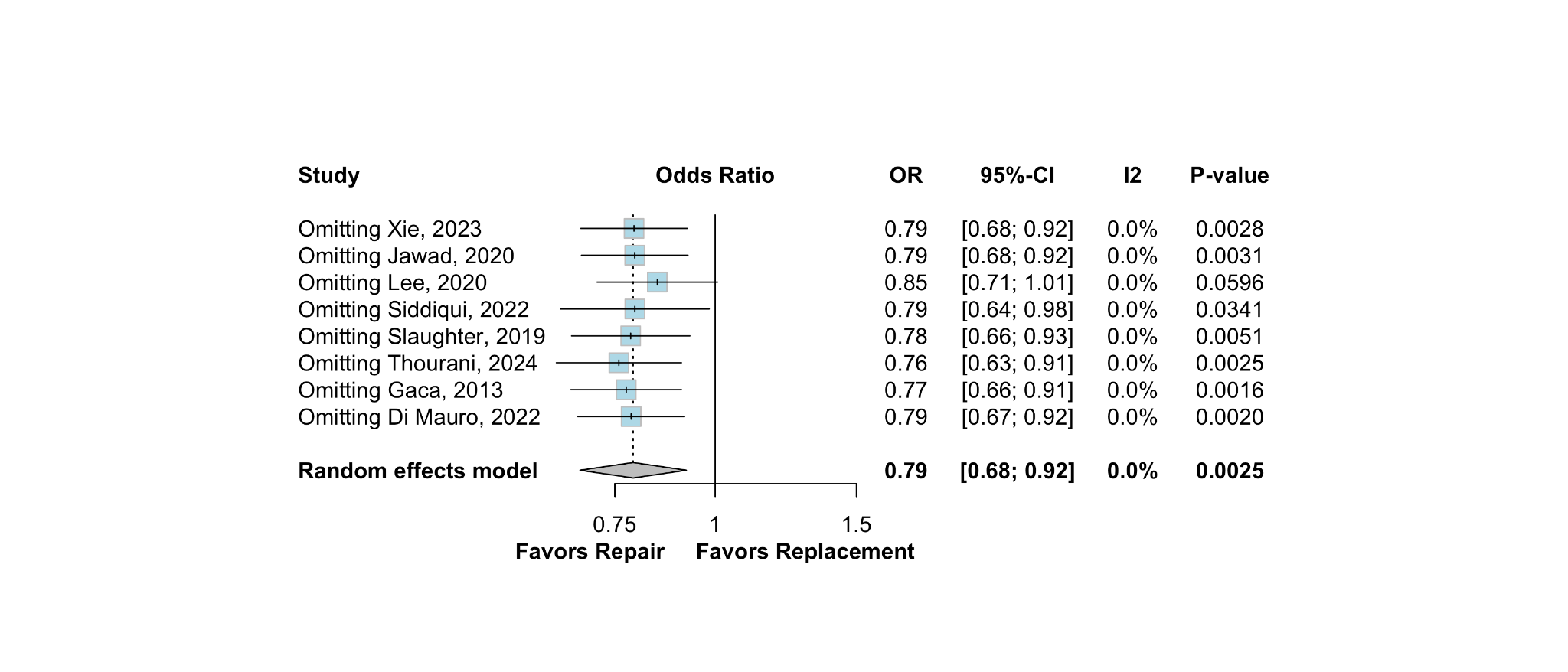


## Supplementary Figure 5G. Leave-one-out for postoperative deep wound infection


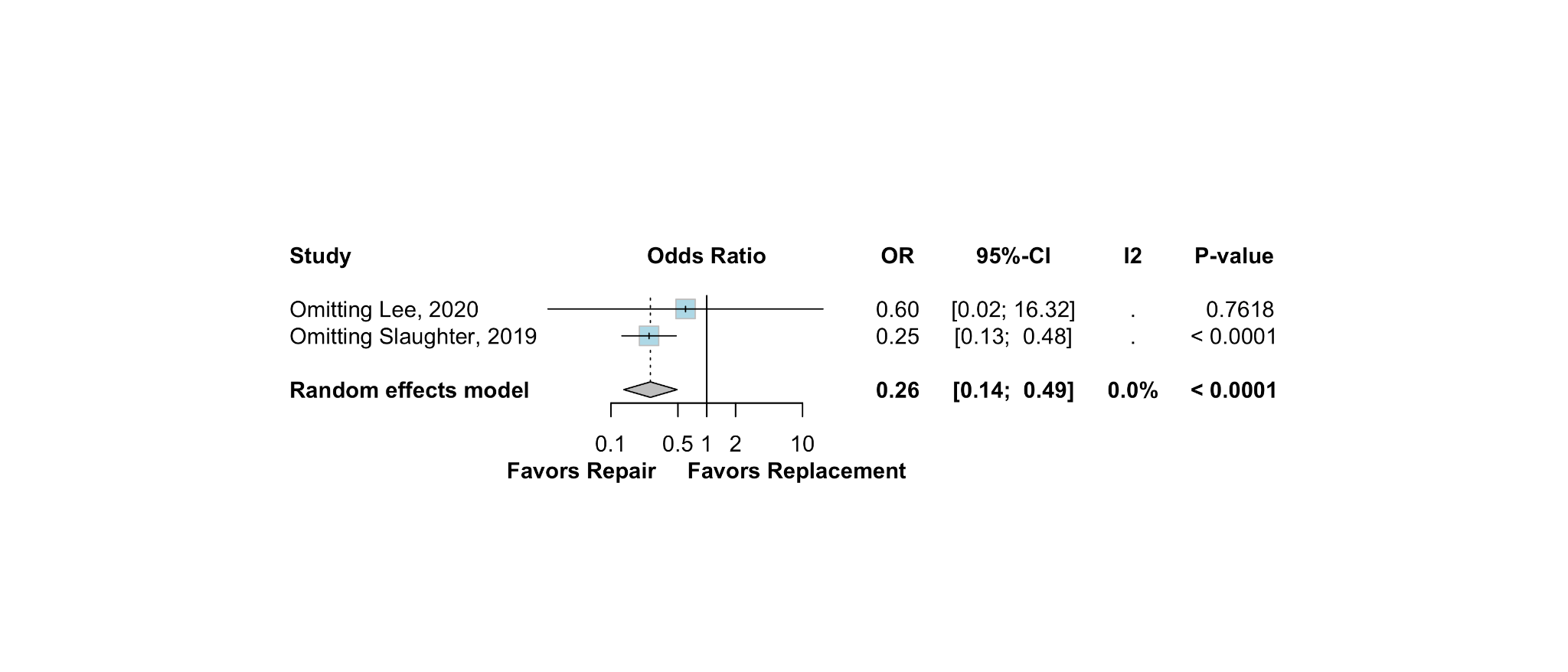


## Supplementary Figure 5H. Leave-one-out for permanent pacemaker implantation


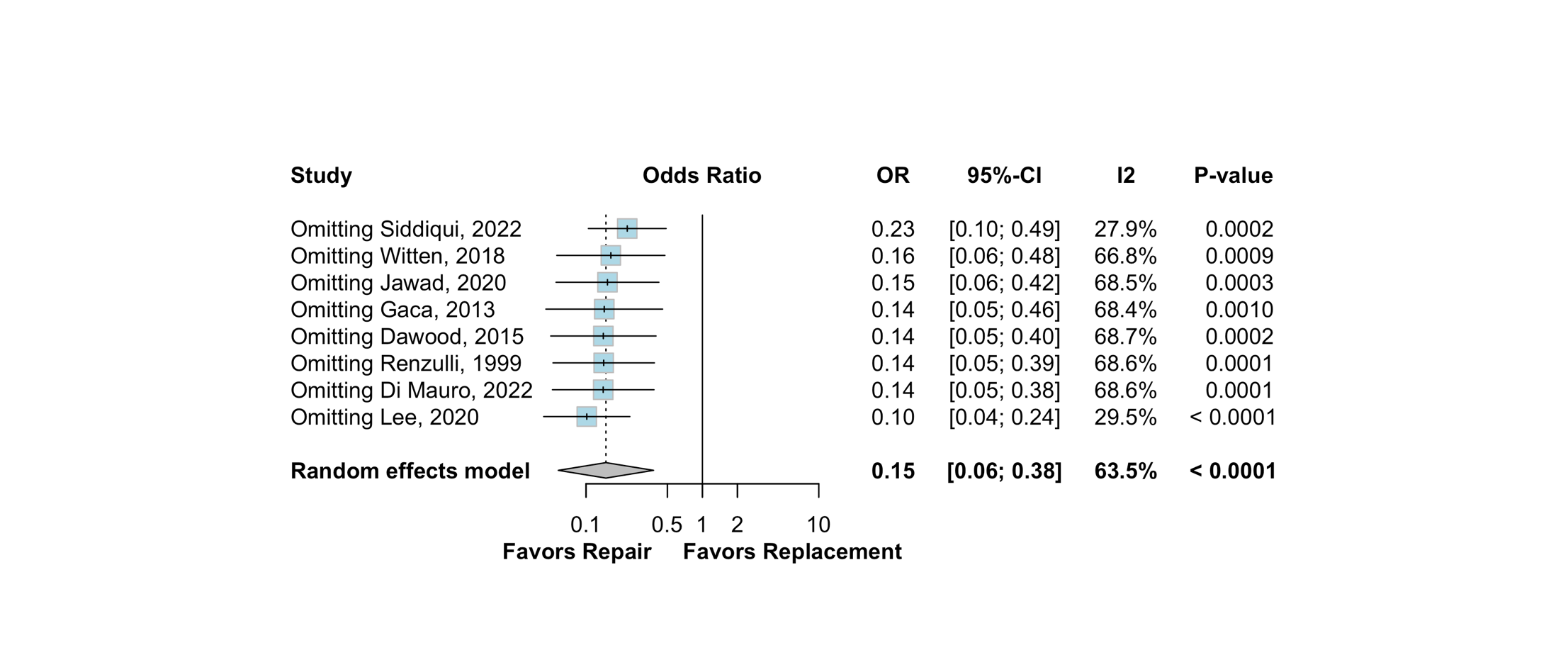


# **Supplementary Figure 6. Post hoc Subgroup Analyses**

## Supplementary Figure 6A. Post hoc subgroup analysis for Risk of Bias

**
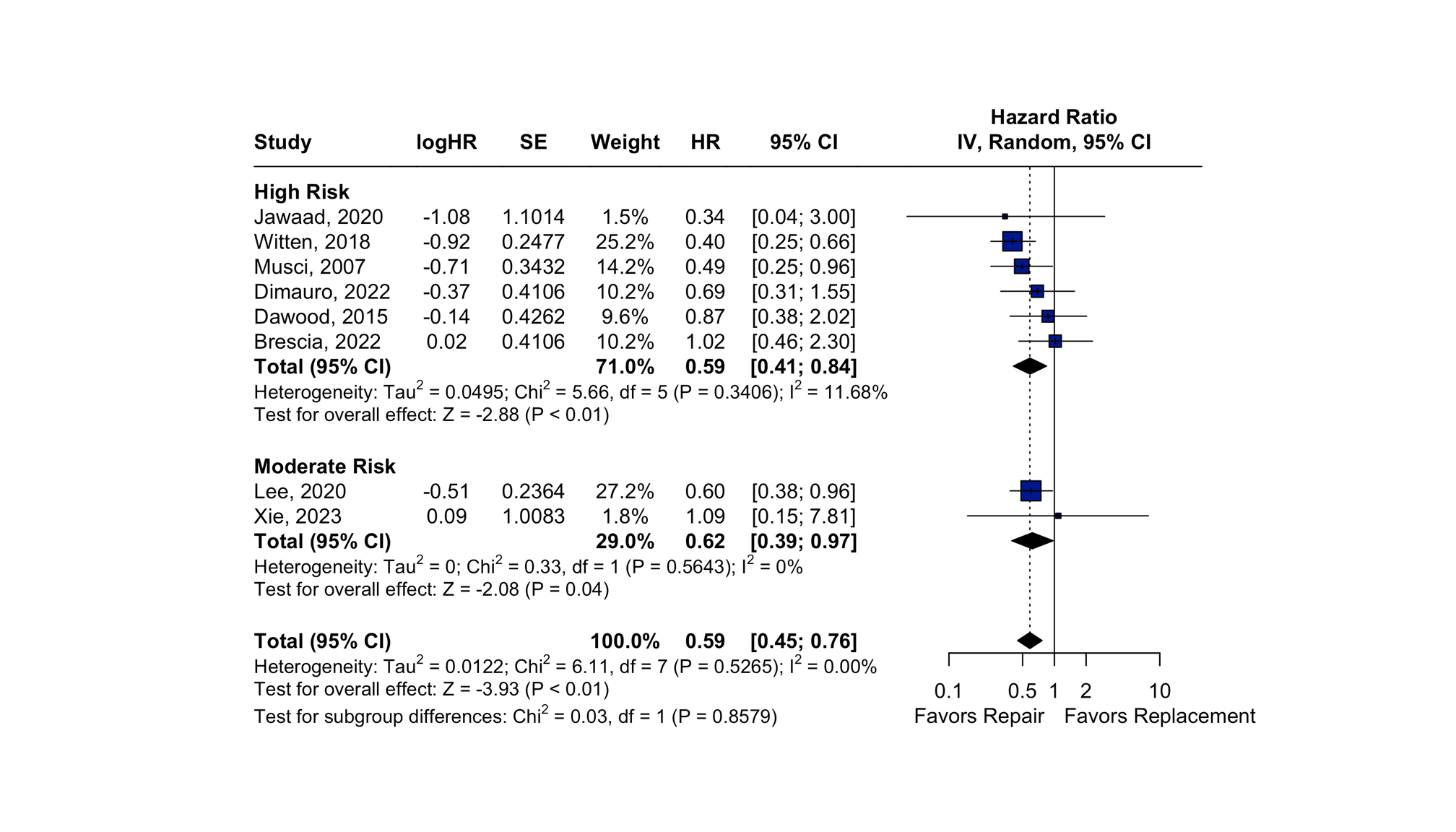
**

## Supplementary Figure 6B. Post hoc subgroup analysis for bioprosthetic versus mechanical valves

**
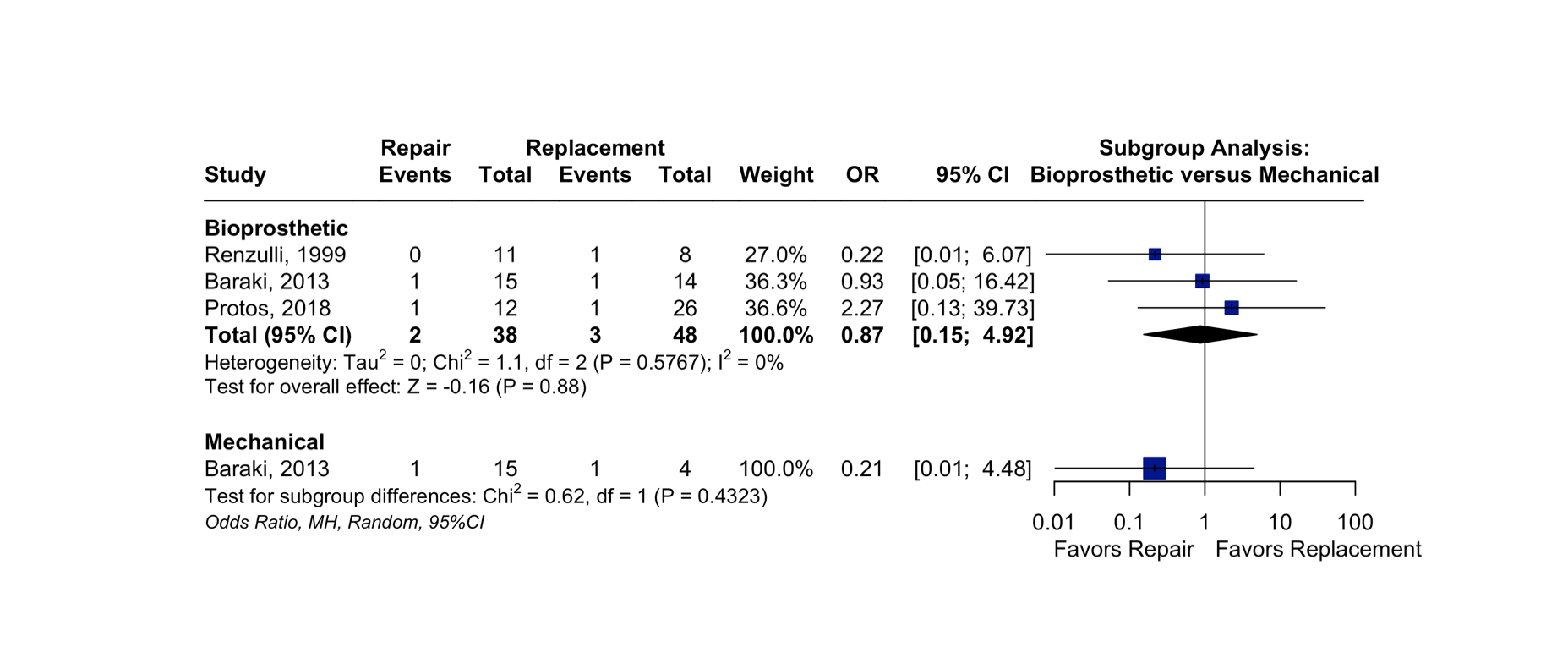
**

# **Supplementary Figure 7. Quality assessment using Risk Of Bias In Non-randomized Studies (ROBINS-I)**

## Supplementary Figure 7A. "Traffic light" plot of the domain-level judgments for each study


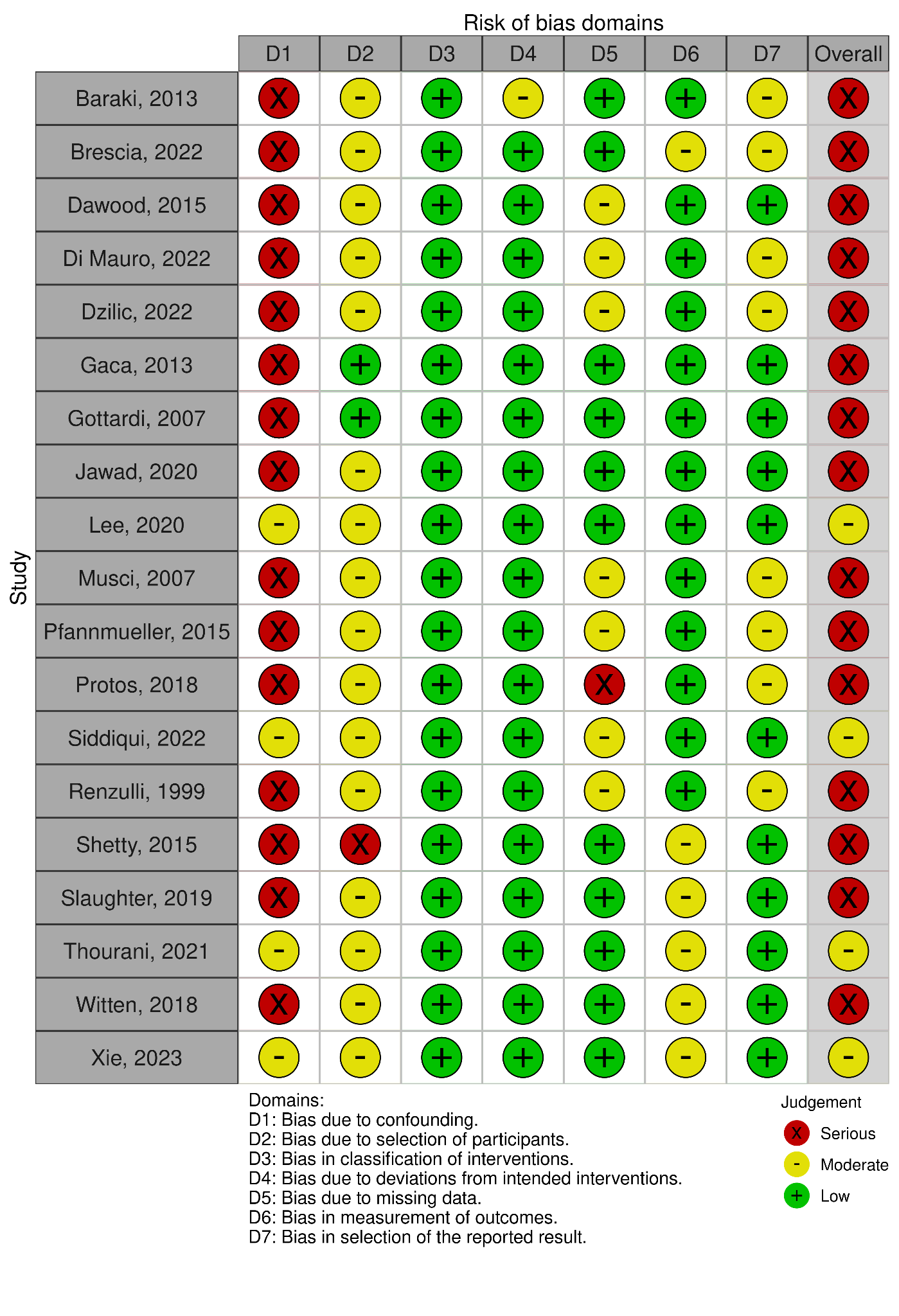


##

## Supplementary Figure 7B. Summary of overall weighted bar plot of risk-of-bias judgments within each bias domain.

##
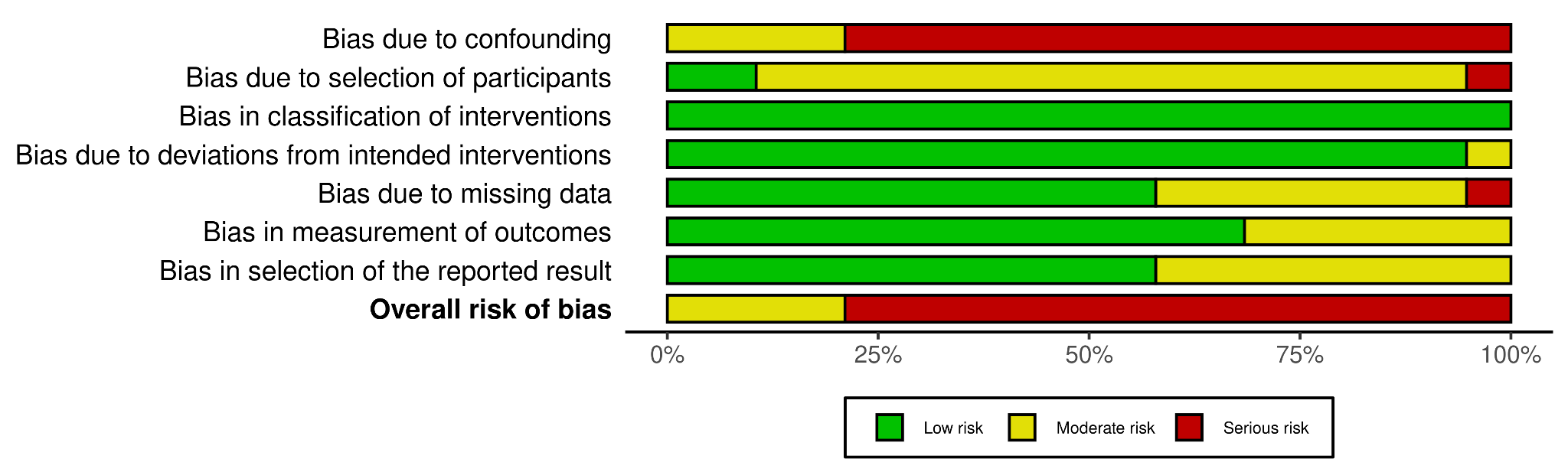


#

# **Supplementary Figure 8. Funnel plot and Egger’s test**

## Supplementary Figure 8A. Funnel plot for long-term all-cause mortality endpoint


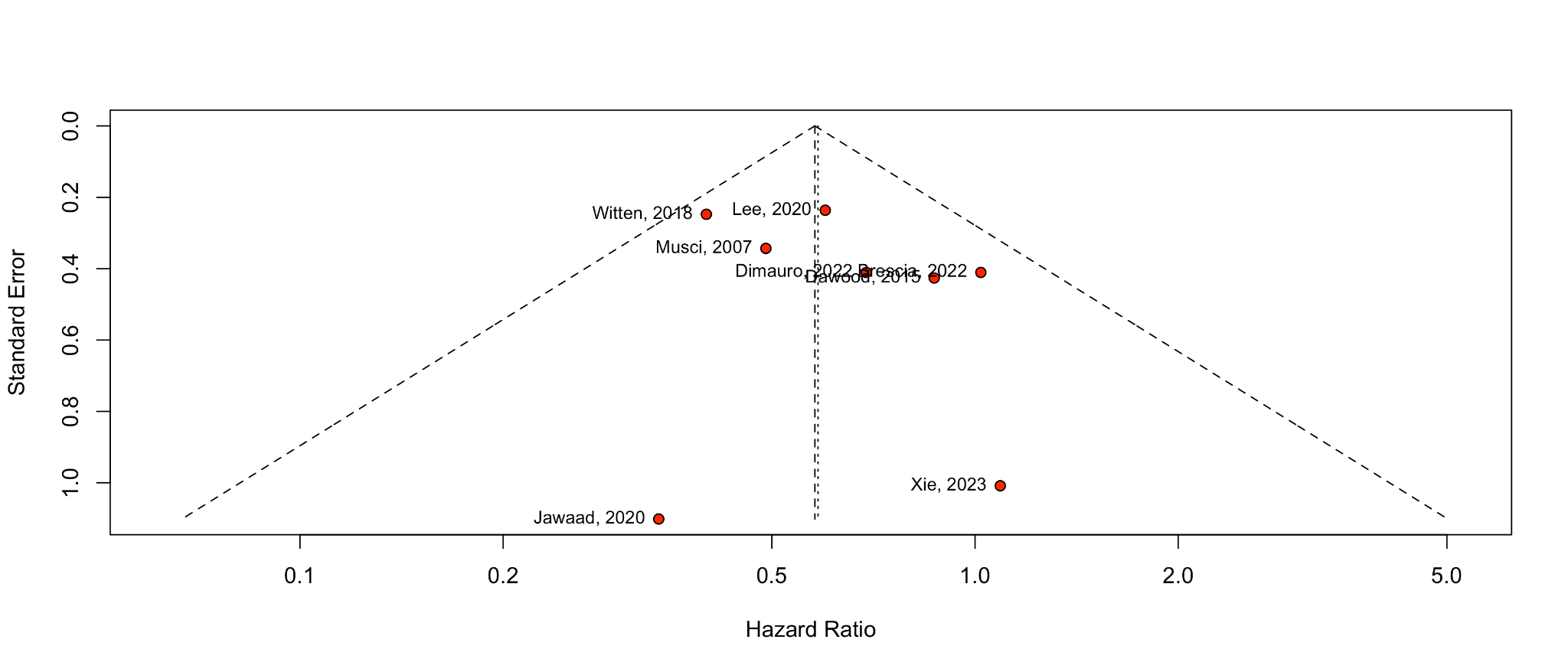


## Supplementary Figure 8B. Funnel plot for any reoperation endpoint


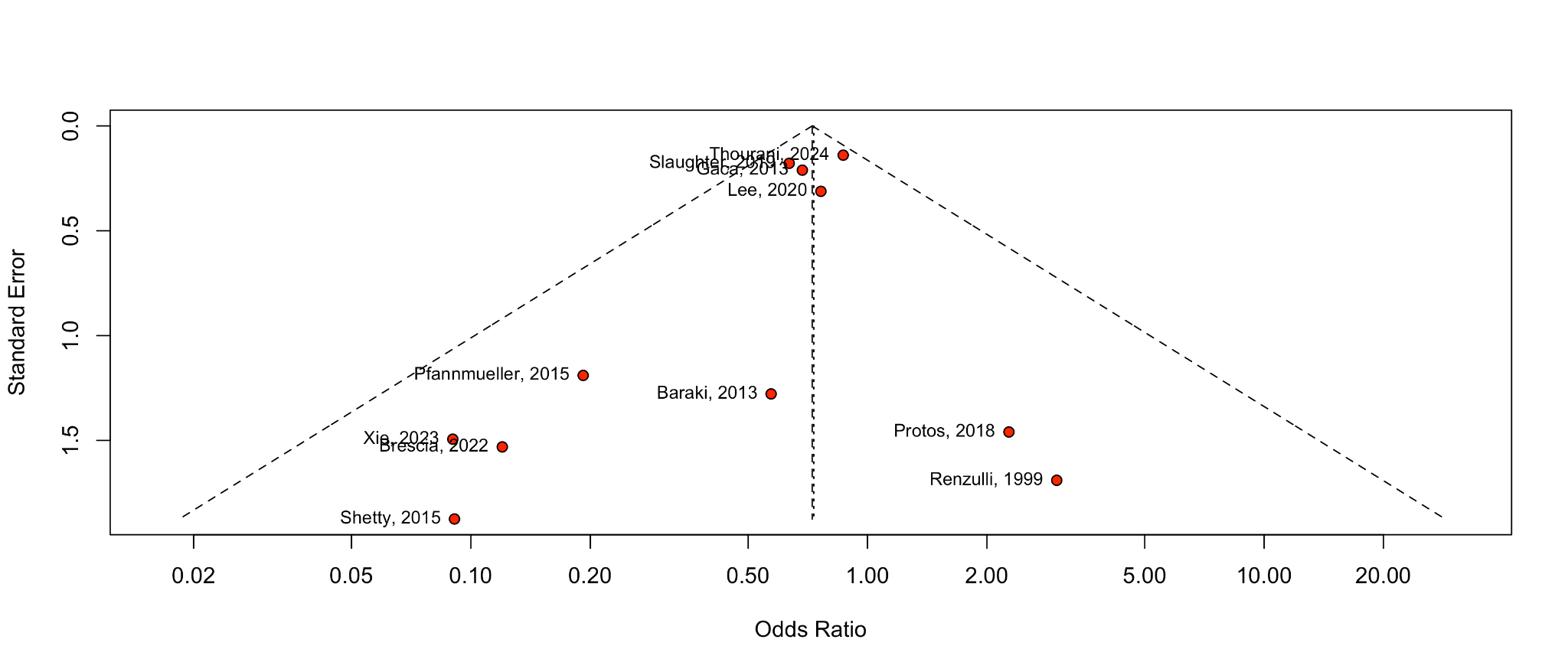


| **Egger’s Regression Test for any reoperation endpoint** | | | |
| --- | --- | --- | --- |
| **Intercept** | **95% CI** | **t** | **p-value** |
| -0.60 | -1.36 to -0.15 | -1.56 | 0.15 |

##

## Supplementary Figure 8C. Funnel plot for reinfection endpoint


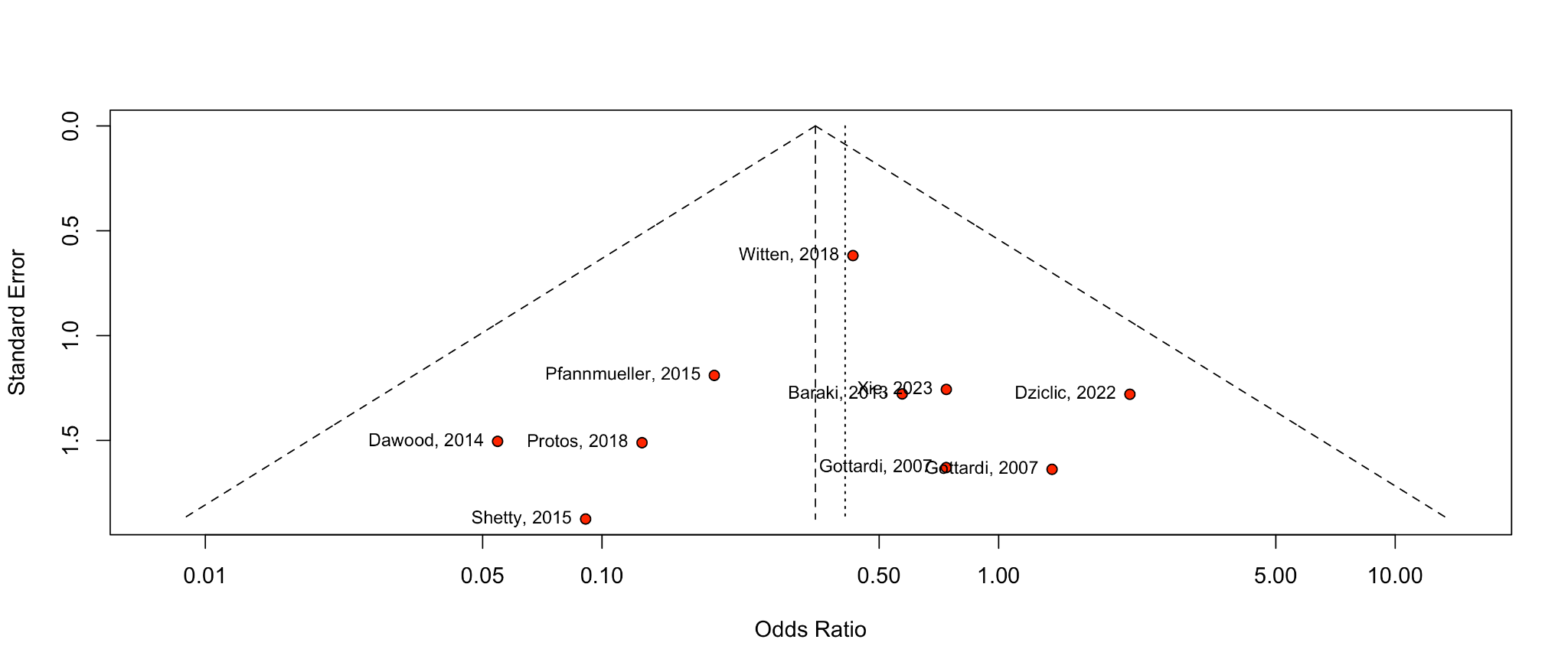


| **Egger’s Regression Test for reinfection endpoint** | | | |
| --- | --- | --- | --- |
| **Intercept** | **95% CI** | **t** | **p-value** |
| -0.32 | -1.86 to -1.23 | -0.40 | 0.70 |

##

## Supplementary Figure 8D. Funnel plot for early mortality endpoint


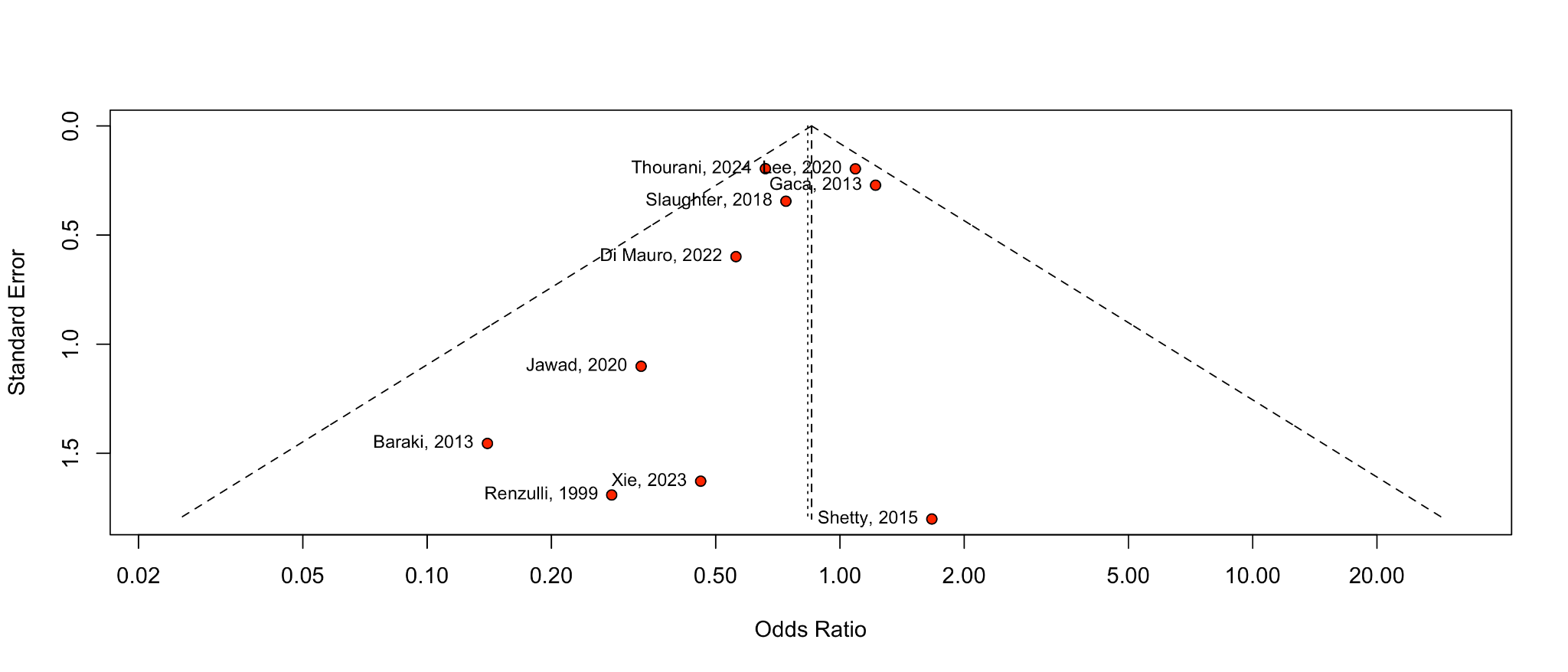


| **Egger’s Regression Test for early endpoint** | | | |
| --- | --- | --- | --- |
| **Intercept** | **95% CI** | **t** | **p-value** |
| -0.67 | -1.58 to -0.25 | -1.43 | 0.19 |

##

## Supplementary Figure 8E. Funnel plot for postoperative stroke endpoint


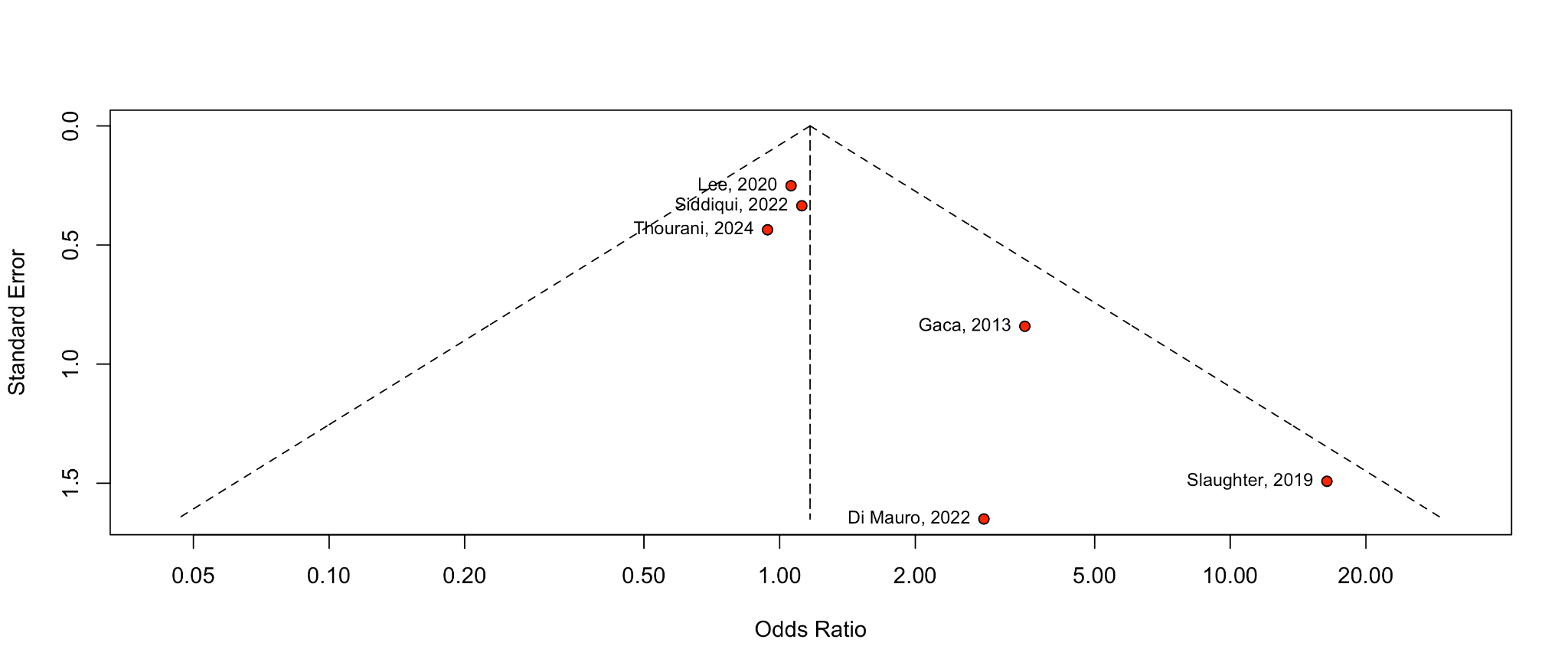


## Supplementary Figure 8F. Funnel plot for acute kidney injury endpoint


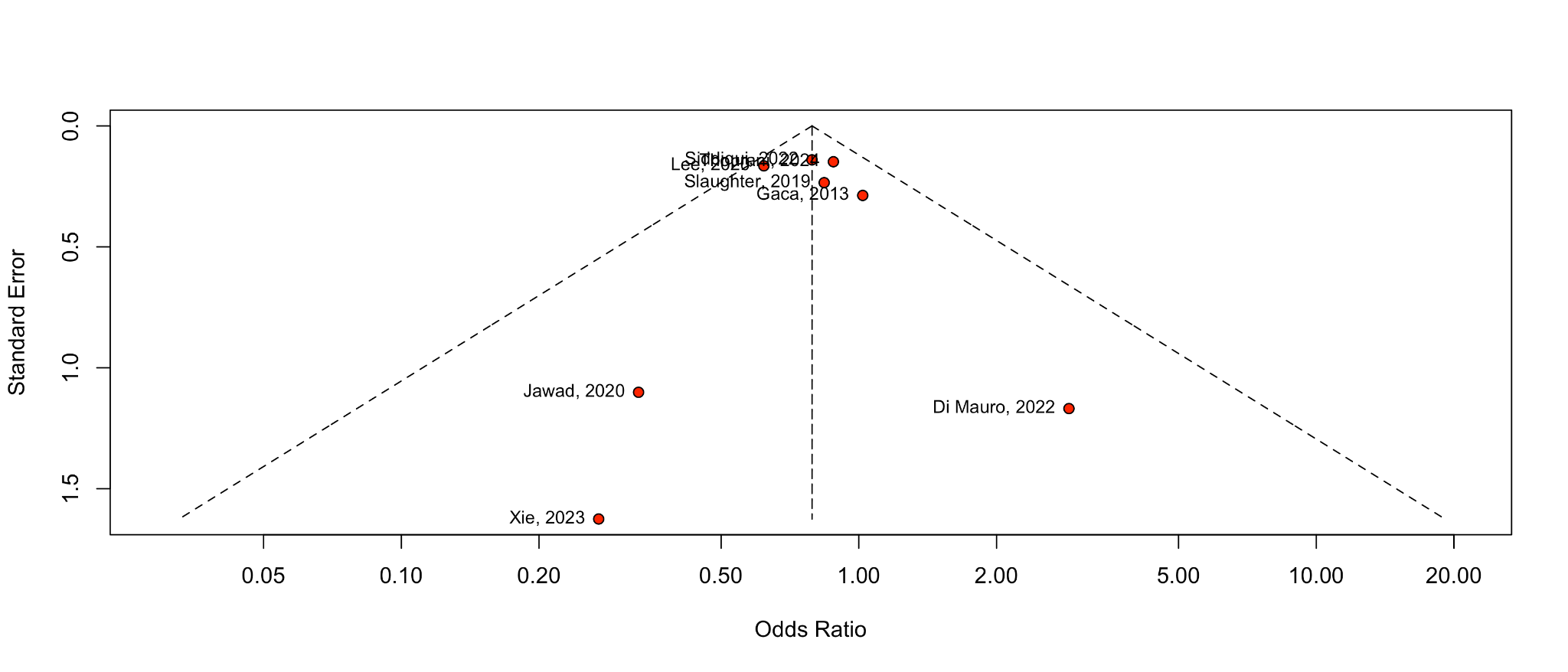


## Supplementary Figure 8G. Funnel plot for postoperative deep wound infection endpoint


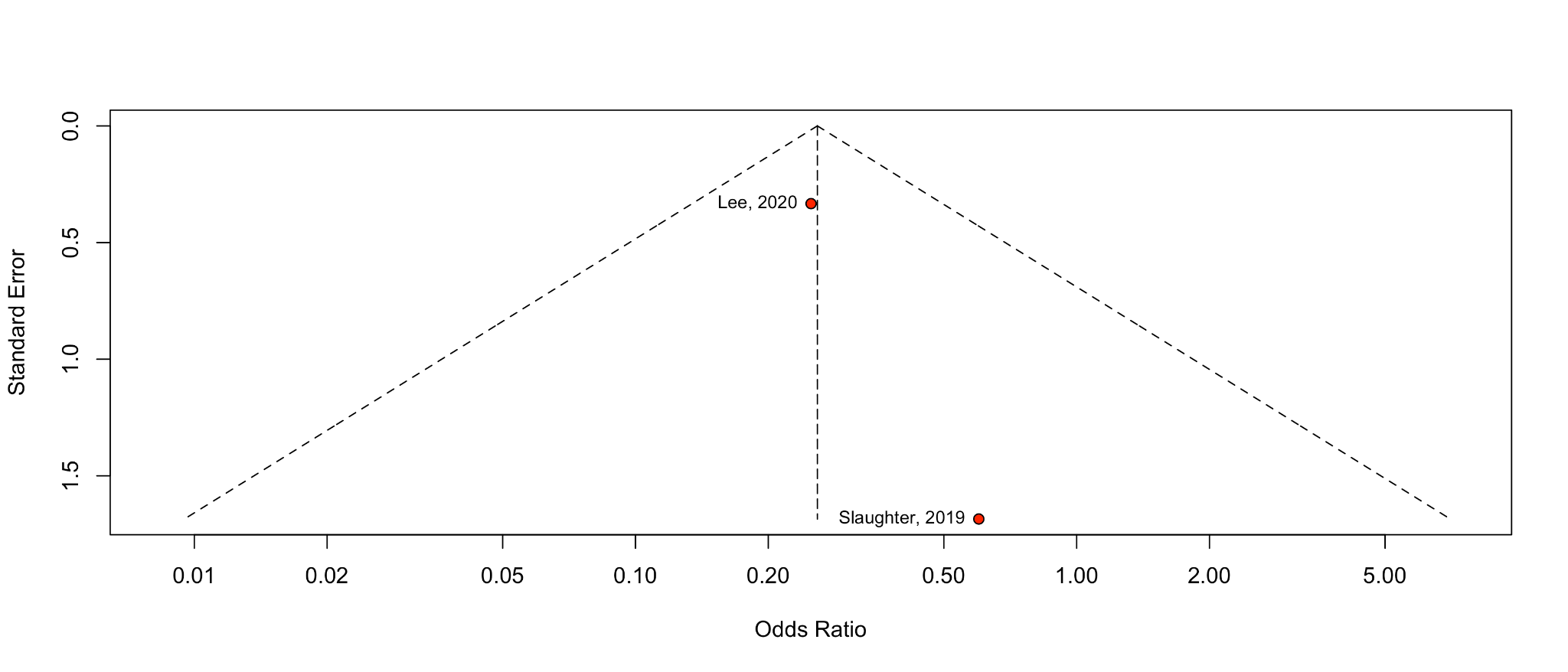


## Supplementary Figure 8H. Funnel plot for permanent pacemaker implantation


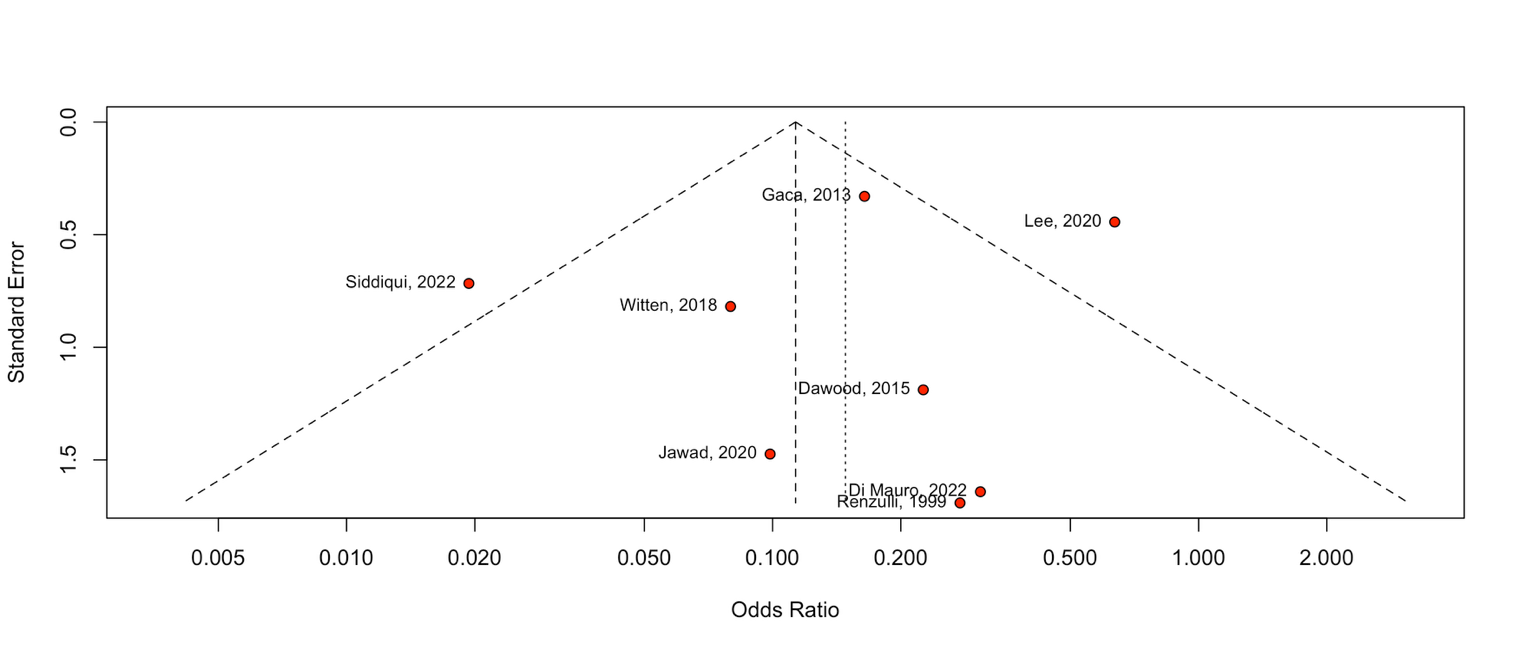


# **References**

1. Akinosoglou K, Apostolakis E, Marangos M, Pasvol G. Native valve right sided infective endocarditis. *Eur J Intern Med*. 2013;24:510–519.

2. Delgado V, Ajmone Marsan N, de Waha S, Bonaros N, Brida M, Burri H, Caselli S, Doenst T, Ederhy S, Erba PA, et al. 2023 ESC Guidelines for the management of endocarditis. *Eur Heart J*. 2023;44:3948–4042.

3. Akinosoglou K, Apostolakis E, Koutsogiannis N, Leivaditis V, Gogos CA. Right-sided infective endocarditis: surgical management. *Eur J Cardiothorac Surg*. 2012;42:470–479.

4. Vervoort D, An KR, Elbatarny M, Tam DY, Quastel A, Verma S, Connelly KA, Yanagawa B, Fremes SE. Dealing With the Epidemic of Endocarditis in People Who Inject Drugs. *Can J Cardiol*. 2022;38:1406–1417.

5. Geirsson A, Schranz A, Jawitz O, Mori M, Feng L, Zwischenberger BA, Iribarne A, Dearani J, Rushing G, Badhwar V, et al. The Evolving Burden of Drug Use Associated Infective Endocarditis in the United States. *Ann Thorac Surg*. 2020;110:1185–1192.

6. Iaccarino A, Barbone A, Basciu A, Cuko E, Droandi G, Galbiati D, Romano G, Citterio E, Fumero A, Scarfò I, et al. Surgical Challenges in Infective Endocarditis: State of the Art. *J Clin Med*. 2023;12:5891.

7. Galeone A, Gardellini J, Perrone F, Francica A, Mazzeo G, Lucchetti MR, Onorati F, Luciani GB. Tricuspid valve repair and replacement for infective endocarditis. *Indian J Thorac Cardiovasc Surg*. 2024;40:100–109.

8. Yanagawa B, Elbatarny M, Verma S, Hill S, Mazine A, Puskas JD, Friedrich JO. Surgical Management of Tricuspid Valve Infective Endocarditis: A Systematic Review and Meta-Analysis. *Ann Thorac Surg*. 2018;106:708–714.

9. Lee H-A, Chou A-H, Wu VC-C, Chan Y-S, Cheng Y-T, Chang C-H, Chang S-H, Hung K-C, Chu P-H, Chen S-W. Nationwide cohort study of tricuspid valve repair versus replacement for infective endocarditis. *Eur J Cardiothorac Surg*. 2021;59:878–886.

10. Witten JC, Hussain ST, Shrestha NK, Gordon SM, Houghtaling PL, Bakaeen FG, Griffin B, Blackstone EH, Pettersson GB. Surgical treatment of right-sided infective endocarditis. *J Thorac Cardiovasc Surg*. 2019;157:1418-1427.e14.

11. Xie L, Chen X, He J, Lin S, Chen X, Wu Q, Chen L, Zhuang J, Qiu Z, Chen L. Comparison of valvuloplasty and replacement for surgical treatment of tricuspid infective endocarditis. *BMC Cardiovasc Disord*. 2023;23:213.

12. Page MJ, McKenzie JE, Bossuyt PM, Boutron I, Hoffmann TC, Mulrow CD, Shamseer L, Tetzlaff JM, Akl EA, Brennan SE, et al. The PRISMA 2020 statement: an updated guideline for reporting systematic reviews. *BMJ*. 2021;n71.

13. Higgins, Julian P. T., Thomas, James, Chandler, Jackie, Cumpston, Miranda, Li, Tianjing, Page, Matthew J., Welch, Vivian A. Cochrane Handbook for Systematic Reviews of Interventions version 6.3 (updated February 2022) [Internet]. Cochrane; 2022. Available from: https://www.training.cochrane.org/handbook

14. Edmunds LH, Clark RE, Cohn LH, Grunkemeier GL, Miller DC, Weisel RD. Guidelines for reporting morbidity and mortality after cardiac valvular operations. *Eur J Cardiothorac Surg*. 1996;10:812–816.

15. Bowdish ME, D’Agostino RS, Thourani VH, Schwann TA, Krohn C, Desai N, Shahian DM, Fernandez FG, Badhwar V. STS Adult Cardiac Surgery Database: 2021 Update on Outcomes, Quality, and Research. *Ann Thorac Surg*. 2021;111:1770–1780.

16. Sterne JA, Hernán MA, Reeves BC, Savović J, Berkman ND, Viswanathan M, Henry D, Altman DG, Ansari MT, Boutron I, et al. ROBINS-I: a tool for assessing risk of bias in non-randomised studies of interventions. *BMJ*. 2016;355:i4919.

17. Guyatt GH, Oxman AD, Vist GE, Kunz R, Falck-Ytter Y, Alonso-Coello P, Schünemann HJ, GRADE Working Group. GRADE: an emerging consensus on rating quality of evidence and strength of recommendations. *BMJ*. 2008;336:924–926.

18. Sterne JAC, Sutton AJ, Ioannidis JPA, Terrin N, Jones DR, Lau J, Carpenter J, Rücker G, Harbord RM, Schmid CH, et al. Recommendations for examining and interpreting funnel plot asymmetry in meta-analyses of randomised controlled trials. *BMJ*. 2011;343:d4002.

19. Egger M, Davey Smith G, Schneider M, Minder C. Bias in meta-analysis detected by a simple, graphical test. *BMJ*. 1997;315:629–634.

20. Xu C, Furuya-Kanamori L, Zorzela L, Lin L, Vohra S. A proposed framework to guide evidence synthesis practice for meta-analysis with zero-events studies. *J Clin Epidemiol*. 2021;135:70–78.

21. Gewehr D, Carvalho PEP. How to perform meta-analysis with R - Exploring Heterogeneity: Leave-one-out Analysis [Internet]. 2023 [cited 2025 Feb 27];Available from: https://rgdoi.net/10.13140/RG.2.2.32130.04805

22. Wei Y, Royston P. Reconstructing time-to-event data from published Kaplan-Meier curves. *Stata J*. 2017;17:786–802.

23. Guyot P, Ades AE, Ouwens MJNM, Welton NJ. Enhanced secondary analysis of survival data: reconstructing the data from published Kaplan-Meier survival curves. *BMC Med Res Methodol*. 2012;12:9.

24. Balduzzi S, Rücker G, Schwarzer G. How to perform a meta-analysis with R: a practical tutorial. *Evid Based Ment Health*. 2019;22:153–160.

25. Baraki H, Saito S, Al Ahmad A, Fleischer B, Schmitto J, Haverich A, Kutschka I. Surgical treatment for isolated tricuspid valve endocarditis- long-term follow-up at a single institution. *Circ J*. 2013;77:2032–2037.

26. Brescia AA, Watt TMF, Rosenbloom LM, Williams AM, Bolling SF, Romano MA. Patient and Surgeon Predictors of Mitral and Tricuspid Valve Repair for Infective Endocarditis. *Semin Thorac Cardiovasc Surg*. 2022;34:67–77.

27. Dawood MY, Cheema FH, Ghoreishi M, Foster NW, Villanueva RM, Salenger R, Griffith BP, Gammie JS. Contemporary outcomes of operations for tricuspid valve infective endocarditis. *Ann Thorac Surg*. 2015;99:539–546.

28. Di Mauro M, Bonalumi G, Giambuzzi I, Dato GMA, Centofanti P, Corte AD, Ratta ED, Cugola D, Merlo M, Santini F, et al. Similar outcome of tricuspid valve repair and replacement for isolated tricuspid infective endocarditis. *J Cardiovasc Med (Hagerstown)*. 2022;23:406–413.

29. Dzilic E, Nöbauer C, Burri M, Voss S, Krane M, Lange R, Vitanova K. Surgical treatment of isolated tricuspid valve endocarditis: Midterm data. *J Card Surg*. 2022;37:2999–3005.

30. Gaca JG, Sheng S, Daneshmand M, Rankin JS, Williams ML, O’Brien SM, Gammie JS. Current outcomes for tricuspid valve infective endocarditis surgery in North America. *Ann Thorac Surg*. 2013;96:1374–1381.

31. Gottardi R, Bialy J, Devyatko E, Tschernich H, Czerny M, Wolner E, Seitelberger R. Midterm follow-up of tricuspid valve reconstruction due to active infective endocarditis. *Ann Thorac Surg*. 2007;84:1943–1948.

32. Abd Al Jawad M, Ammar A, Nahas Y, Ahmed A, Kilany I, El Kerdany A. Evaluation of Tricuspid Valve Repair Without Annuloplasty Ring in Intravenous Drug Abusers. *Ann Thorac Surg*. 2020;109:1217–1225.

33. Musci M, Siniawski H, Pasic M, Grauhan O, Weng Y, Meyer R, Yankah CA, Hetzer R. Surgical treatment of right-sided active infective endocarditis with or without involvement of the left heart: 20-year single center experience. *Eur J Cardiothorac Surg*. 2007;32:118–125.

34. Pfannmueller B, Kahmann M, Davierwala P, Misfeld M, Bakhtiary F, Binner C, Etz C, Mohr FW. Tricuspid Valve Surgery in Patients with Isolated Tricuspid Valve Endocarditis: Analysis of Perioperative Parameters and Long-Term Outcomes. *Thorac Cardiovasc Surg*. 2017;65:626–633.

35. Protos AN, Trivedi JR, Whited WM, Rogers MP, Owolabi U, Grubb KJ, Sell-Dottin K, Slaughter MS. Valvectomy Versus Replacement for the Surgical Treatment of Tricuspid Endocarditis. *Ann Thorac Surg*. 2018;106:664–669.

36. Renzulli A, De Feo M, Carozza A, Della Corte A, Gregorio R, Ismeno G, Cotrufo M. Surgery for tricuspid valve endocarditis: a selective approach. *Heart Vessels*. 1999;14:163–169.

37. Siddiqui E, Alviar CL, Ramachandran A, Flattery E, Bernard S, Xia Y, Nayar A, Keller N, Bangalore S. Outcomes After Tricuspid Valve Operations in Patients With Drug-Use Infective Endocarditis. *Am J Cardiol*. 2022;185:80–86.

38. Shetty N, Nagpal D, Koivu S, Mrkobrada M. Surgical and Medical Management of Isolated Tricuspid Valve Infective Endocarditis in Intravenous Drug Users. *J Card Surg*. 2016;31:83–88.

39. Slaughter MS, Badhwar V, Ising M, Ganzel BL, Sell-Dottin K, Jawitz OK, Zhang S, Trivedi JR. Optimum surgical treatment for tricuspid valve infective endocarditis: An analysis of the Society of Thoracic Surgeons national database. *J Thorac Cardiovasc Surg*. 2021;161:1227-1235.e1.

40. Thourani VH, Bonnell L, Wyler von Ballmoos MC, Mehaffey JH, Bowdish M, Kurlansky P, Jacobs JP, O’Brien S, Shahian DM, Badhwar V. Outcomes of Isolated Tricuspid Valve Surgery: A Society of Thoracic Surgeons Analysis and Risk Model. *Ann Thorac Surg*. 2024;118:873–881.

41. Huang X, Gu C, Men X, Zhang J, You B, Zhang H, Wei H, Li J. Repair of functional tricuspid regurgitation: comparison between suture annuloplasty and rings annuloplasty. *Ann Thorac Surg*. 2014;97:1286–1292.

42. Amedi A, Onohara D, Xu D, Suresh KS, Padala M. Hemodynamic outcomes after undersizing ring annuloplasty and focal suture annuloplasty for surgical repair of functional tricuspid regurgitation. *J Thorac Cardiovasc Surg*. 2022;164:76-87.e1.

43. Comentale G, Ahmadi-Hadad A, Moldon HJ, Carbone A, Manzo R, Macchio CC, Damiano A, Bossone E, Esposito G, Pilato E. Comparative Outcomes of Mitral Valve Repair versus Replacement in Infective Endocarditis: A 16-Year Meta-Analysis of Time-to-Event Data from Over 4000 Patients. *Am J Cardiol*. 2025;S0002-9149(25)00136–5.

44. Choi JW, Jang M-J, Kim KH, Hwang HY. Repair versus replacement for the surgical correction of tricuspid regurgitation: a meta-analysis. *Eur J Cardiothorac Surg*. 2018;53:748–755.

45. Doenst T, Caldonazo T, Mukharyamov M, Tasoudis P, Kirov H. Survival Correlates with Regurgitation Degree Before and After Invasive Atrioventricular Valve Treatment. *Thorac Cardiovasc Surg*. 2024;

46. Pettersson GB, Hussain ST. Current AATS guidelines on surgical treatment of infective endocarditis. *Ann Cardiothorac Surg*. 2019;8:630–644.

47. Truong S, Petersen J, Schmiegelow MDS, Due H, Havers-Borgersen E, Smerup M, Køber L, Fosbøl E, Østergaard L. Incidence and factors associated with mitral valve reoperation in patients undergoing surgery for mitral regurgitation: A nationwide cohort study. *Int J Cardiol*. 2025;418:132608.

48. Akins CW, Miller DC, Turina MI, Kouchoukos NT, Blackstone EH, Grunkemeier GL, Takkenberg JJM, David TE, Butchart EG, Adams DH, et al. Guidelines for reporting mortality and morbidity after cardiac valve interventions. *Ann Thorac Surg*. 2008;85:1490–1495.
